# Yeast elongation factor homolog New1 protects a subset of mRNAs from degradation by no-go decay

**DOI:** 10.1093/nar/gkag047

**Published:** 2026-01-29

**Authors:** Max Müller, Lena Sophie Tittel, Elisabeth Petfalski, Kaushik Viswanathan Iyer, Alina-Andrea Kraft, Stefan Pastore, Tamer Butto, Marie-Luise Winz

**Affiliations:** Institute of Pharmaceutical and Biomedical Sciences, Johannes Gutenberg-University Mainz, Staudingerweg 5, 5128 Mainz, Germany; Institute of Pharmaceutical and Biomedical Sciences, Johannes Gutenberg-University Mainz, Staudingerweg 5, 5128 Mainz, Germany; Centre for Cell Biology, University of Edinburgh, Edinburgh EH9 1BF, United Kingdom; Institute of Pharmaceutical and Biomedical Sciences, Johannes Gutenberg-University Mainz, Staudingerweg 5, 5128 Mainz, Germany; Institute of Pharmaceutical and Biomedical Sciences, Johannes Gutenberg-University Mainz, Staudingerweg 5, 5128 Mainz, Germany; Institute of Pharmaceutical and Biomedical Sciences, Johannes Gutenberg-University Mainz, Staudingerweg 5, 5128 Mainz, Germany; Institute of Pharmaceutical and Biomedical Sciences, Johannes Gutenberg-University Mainz, Staudingerweg 5, 5128 Mainz, Germany; Institute of Pharmaceutical and Biomedical Sciences, Johannes Gutenberg-University Mainz, Staudingerweg 5, 5128 Mainz, Germany

## Abstract

New1 is a homologue of the essential yeast translation elongation factor eEF3. Lack of New1 has been shown to induce ribosome queuing upstream of the stop codon on messenger RNAs (mRNAs) with specific C-terminal lysine and arginine codons. Here, we used ultraviolet crosslinking and analysis of complementary DNA (cDNA), long-read nanopore sequencing, and proteomics to address the consequences such queues have for the yeast cell. We show that these queues represent collisions, recognized by collision sensor Hel2, triggering mRNA degradation via canonical no-go decay (NGD). We identified 139 target mRNAs, on which decay is initiated by Cue2-mediated cleavage upstream of the stop codon. Compared to other collision-prone mRNAs, ending on the same C-terminal codons, these targets are characterized by stronger secondary structures upstream of the stop codon, longer queues, and stronger queuing signatures. Nanopore sequencing enabled characterization of NGD cleavage fragments across targets. Ultimately, NGD in the absence of New1 leads to downregulation of encoded proteins, including highly abundant and essential metabolic enzymes like Pgk1 and Gpm1, as well as translation elongation factors such as eEF1-alpha and eEF1-beta. We show that New1 protects such mRNAs from degradation by NGD and that NGD is a major determinant of the cold sensitive growth phenotype observed in *NEW1* deletants.

## Graphical Abstract

**Graphical Abstract:**
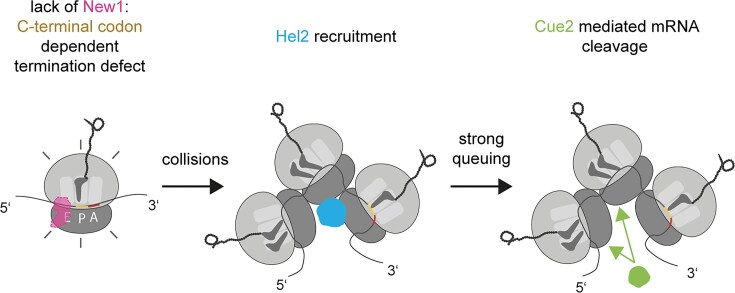

## Introduction

Ribosomal translation of genetic information from messenger RNA (mRNA) into proteins is a highly conserved key process in all living organisms. While translation normally proceeds from start to stop codon, many different problems can occur during translation elongation. Those potentially lead to ribosomal stalling and collision of subsequent ribosomes. This way collided di-ribosomes (disomes) or higher-order polysomes are formed [1–3]. Reasons for ribosome stalling and hence, collisions can be found at the levels of mRNAs, transfer RNAs (tRNAs), and ribosomes themselves. At the mRNA level, translation roadblocks can occur as stable secondary structures [4], chemical damage, such as oxidation and alkylation [5, 6], truncation [7], as well as poorly translatable codon sequences. For example, poly(A), coding for poly(lysine), is difficult to translate in both, yeast and mammals due to its structure during mRNA accommodation in the ribosomal A-site, which impedes binding of the aminoacylated lysine tRNA [8, 9]. On the other hand, in yeast, the arginine codon ‘CGA’ is decoded slowly due to poor base pairing and low abundance of its decoding tRNA-R(ICG) [9, 10]. In addition, lack of specific (aminoacylated) tRNAs, e.g. during amino acid starvation [11] and features of the ribosome, such as faulty ribosomal RNA (rRNA), as well as translation inhibitors like cycloheximide, anisomycin and other antibiotics can also cause stalling and collisions, triggering quality control [3, 12–14].

Ribosome stalling not only causes the production of potentially deleterious truncated proteins, but ribosome collisions can also induce frameshifting [15, 16]. Therefore, different translation quality control pathways have evolved, as reviewed in [17, 18]. While ribosome-associated quality control (RQC) degrades truncated nascent peptides [19], no-go decay (NGD) removes stalling-prone mRNAs [4]. The E3 ubiquitin ligase Hel2 (ZNF598 in mammals) is central to collision sensing and triggering of RQC and NGD [2, 16, 20, 21]). It binds to the disome interface formed by the small subunits of collided ribosomes and polyubiquitinates ribosomal proteins uS10 and uS3 (uS3, uS10, and eS10 in mammals [16, 22, 23]; in *Saccharomyces cerevisiae* the target lysines in eS10 are missing, contrary to other fungi such as *Schizosaccharomyces pombe* [21]). This ubiquitination mark helps recruit the RQC trigger (RQT) complex, consisting of Slh1 and ubiquitin binding proteins Cue3 and Rqt4 in yeast [20, 24, 25] (ASCC3, ASCC2 and TRIP-4/ASC-1in mammals [20, 26, 27]). The helicase Slh1 splits leading ribosomes in collisions in an ATP-dependent manner [20, 25, 27–29]. Mechanistically, Slh1 has been proposed to pull on the mRNA protruding from the leading ribosome, whereby the colliding ribosome acts as a wedge to split the small from the large subunit [25]. Both, Cue3 and Rqt4 bind to K63-linked polyubiquitin [30], thereby facilitating recruitment to collided ribosomes. They bind to and stabilize the Slh1 structure [25], but cause only mild defects in splitting ribosomes when lacking individually [20, 24, 26, 27]. However, lack of both at the same time causes defects resembling those seen upon *SLH1* deletion [20]. Since Slh1 requires an mRNA overhang for its activity, the RQT complex cannot split ribosomes that are stalled at the 3′-end of the transcript, where the entry channel is not occupied by mRNA. Splitting of such ribosomes requires Dom34 (PELOTA in mammals) and Hbs1, which structurally resemble the canonical release factors of translation termination, eRF1 and eRF3 [31–33]. For splitting of 3′-end stalled ribosomes, a third factor, Rli1 (ABCE1 in mammals) is required.

As previously reviewed [17, 18, 34–43], splitting yields a free small subunit, as well as a peptidyl–tRNA stuck in a large subunit. This peptide will be degraded by RQC and/or CAT-tailing, or may be released by peptide release factors [44–46], also in an RQC complex-independent manner [46]. At the same time, the mRNA in a collision can also be targeted for degradation by NGD. Here, several pathways have been described, where the canonical NGD pathway relies on ribosomal protein polyubiquitination by Hel2 [2, 21], and endonucleolytic cleavage by Cue2 [28]. An RQC-coupled, and an RQC-uncoupled pathway have been described [2, 47]. RQC-coupled NGD relies on ribosome splitting by Slh1, followed by endonucleolytic cleavage, which was first reported to take place in the A-site of the collided ribosome [2]. However, cleavage between A- and P-site, as well as in the E-site have been apparent in a more recent report [47]. On the other hand, splitting is dispensable for RQC-uncoupled NGD, where cleavage occurs upstream of the collided ribosome, and ribosomal protein eS7 is first monoubiquitinated by Not4, and then polyubiquitinated by Hel2 [2, 47]. Upon endonucleolytic cleavage, the resulting 3′-fragment is degraded 5′-to-3′ by exonuclease Xrn1, whereas the 5′-fragment is degraded by the RNA exosome, with help from the Ski complex [4]. While the original publication [4] described the splitting factor Dom34 [48] to be essential for canonical NGD, Cue2-dependent cleavage was shown to still occur in the absence of Dom34 in other reports [28, 48]. Although canonical, endonuclease-based NGD was described first, it appears that the majority of mRNAs bearing collided ribosomes are degraded 5′-to-3′ by Xrn1, without the need for Cue2 [28], but potentially with the help of two additional factors, Smy2 and Syh1 [49].

We were interested in identifying novel players in translation quality control. Inspecting genetic interactions with the genes encoding Hel2 and other NGD factors in yeast *S. cerevisiae*, we identified the *NEW1* gene. *NEW1* displayed positive genetic interactions (characterized by decreases in fitness that are lower in double deletants than expected, judging by the fitness of single deletants) with *HEL2, CUE2, DOM34, XRN1*, and *SKI3* (encoding part of the Ski complex, needed for exosome function). Indeed, for *HEL2, NEW1* displayed the highest positive genetic interaction of all genes in the dataset [50]. On the other hand, *NEW1* displayed negative genetic interactions (fitness loss greater than expected) with all three genes encoding yeast RQT factors (Supplementary Fig. S1). New1 is an ABCF protein [51] with close homology to fungal specific and essential translation elongation factor eEF3, encoded by the *YEF3* gene, but which contains an additional N-terminal prion domain in saccharomycetal yeasts [52–54]. New1 and eEF3 are broadly distributed across fungi [54]. eEF3 has been shown to be involved in ribosome translocation, where it facilitates dissociation of the E-site tRNA [55], and in A-site tRNA selection [56, 57]. At this site, eEF3 was also shown to physically interact with eEF1A, thereby stimulating eEF1A’s function of loading aminoacyl-tRNAs within the ribosomal A-site [56, 58, 59].

Furthermore, eEF3 stimulates translation termination by eRF1/eRF3 [60], participates in the release of mRNA and tRNA from the ribosome in post-termination complexes [61] and its lack was reported to cause stop codon readthrough [60]. Besides roles in translation, eEF3 was also shown to associate with mRNA independently from ribosomes [62]. In comparison, less is known about the roles of the eEF3 homolog, New1. New1 was shown to associate with mRNA [62], with polysomes [63] and with ribosomes throughout the elongation cycle, including terminating ribosomes [64]. Lack of New1 had been shown to cause a cold sensitivity phenotype, as well as a ribosome biogenesis defect [65], but has also been reported to cause ribosomal queuing on C-terminal arginine (R) or lysine (K) codons, suggesting a role in translation termination and/or ribosome recycling [63], possibly similar to eEF3. A more recent follow-up study, published during writing of our initial manuscript, found that, in addition to the reported amino-acid specificity, queuing depended on the specific codon, rather than amino acid identity and showed an influence of the decoding tRNA [64]. Interestingly, overexpression of New1 can rescue the growth defect caused by eEF3 depletion to some extent [63]. Conversely, overexpression of eEF3 cannot rescue defects caused by lack of New1 [63], suggesting that New1 can fulfil several functions, at least one of which is not shared with eEF3. Noteworthy, the chromo domain of New1 is truncated, compared to that of eEF3 [63]. For eEF3, this domain has been shown to interact with the ribosomal protein L1, thereby opening the L1 stalk, to facilitate the release of the E-site tRNA [55]. Mutations in eEF3’s chromo domain impaired translation elongation and reduced ATPase activity, highlighting the importance of this domain also in translation [66]. Kasari and colleagues [63] hypothesized that, due to the truncation of this structure in New1, its overexpression cannot fully compensate for the lack of eEF3.

Depletion of eEF3 leads to the accumulation of ribosomes with tRNAs occupying the A-, P-, and E-sites [55]. Interestingly, in addition to ribosomes with an open L1 stalk and tRNAs in the P- and E-sites, New1 was also visualized on a subpopulation of ribosomes harbouring tRNAs in all three binding sites with the L1 stalk in a closed conformation [64].

Taking into account the genetic interactions with translation quality control factors, in this work, we use a combination of biochemical experiments, ultraviolet (UV) crosslinking and analysis of complementary DNA (cDNA; CRAC) [67], (long-read) nanopore sequencing, and proteomics to study effects of *NEW1* deletion on translation quality control. We discover that ribosomal queues in the absence of New1 represent ribosome collisions that are sensed by the translation quality control trigger factor Hel2. As recently observed [64], we also find that collisions occur at specific C-terminal codons, rather than being defined by the identity of the encoded amino acid. Further, we show that these collisions trigger canonical NGD for a subset of affected mRNAs, which is initiated by Cue2-mediated cleavage upstream of the stop codon, and is stimulated by cleavage factor Dom34. NGD is aggravated when the RQT complex is dysfunctional. NGD targets exhibit more frequent and longer queues, and are defined by a stronger mRNA folding propensity at several nucleotide positions in proximity of the stop codon. Several of these mRNAs encode essential metabolic enzymes including phosphoglycerate kinase 1 (Pgk1) and various factors of the translation machinery. Consequently, we demonstrate that the gene products of mRNAs targeted for NGD also tend to be reduced at the protein level. Inhibiting canonical NGD by deletion of the *CUE2* gene rescues the severe growth phenotype observed at cold temperatures for *NEW1* deletants to almost wildtype level, suggesting that NGD is the main driver of cold sensitivity previously observed [63–65] in yeast lacking New1. Finally, we confirm that New1 associates not only with ribosomal RNA, but also with tRNA and mRNA. For the latter, we show a preference for 3′-ends. Our results indicate that New1 protects specific mRNAs from ribosomal collisions and ensuing NGD, with crucial effects on yeast proteostasis.

## Materials and methods

### Strains, oligonucleotides, plasmids, and other materials

All yeast strains, oligonucleotides, plasmids, general materials, media, buffers, polyacrylamide gel components, and devices utilized in this project are listed in Supplementary Tables S1–S8, respectively. Construction of plasmids and strains is described in Supplementary Methods.

### Crosslinking and analysis of cDNA

CRAC was performed as previously described [21] with the following differences: For culturing at 20°C, -URA-TRP media (lacking uracil and tryptophan) was used, whereas for culturing at 30°C, -TRP media was used. All samples were crosslinked at an optical density (OD_600_) of ∼0.5, in a Vari-X-Link crosslinker (UVO3), with a UV 254 nm dose of 120 mJ/cm^2^, using a total of 0.7 l culture for experiments at 30°C, or 1.0 l culture for experiments at 20°C. For 30°C samples, all further steps were performed as previously described [21]. For 20°C samples, the following differences in library preparation applied: For purification of tagged proteins, magnetic beads were used. Magnetic immunoglobulin G (IgG) beads were prepared from 2 ml tosyl activated M280 Dynabeads, by removing supernatant on the magnet, washing three times with 2 ml 0.1 M sodium phosphate, pH 7.4, mixing by vortexing and removing supernatant. Then, 200 µl IgG from rabbit serum (14 mg/ml in milliQ water) and 800 µl 0.1 M sodium phosphate, pH 7.4 were mixed with the beads by vortexing, then 700 µl of 3 M ammonium sulfate were added, mixed by vortexing, and incubated overnight at 37°C. Upon removal of supernatant, 2 ml phosphate buffered saline (PBS), 0.5% Tween 20, pH 7.4, and 200 µl bovine serum albumin (10 mg/ml in milliQ water) were mixed with the beads and incubated for 1 h at 37°C. Supernatant was removed and 2 ml PBS, 0.1% Tween 20, pH 7.4 were added and washed (rotating) three times for 5 min. Beads were resuspended in 1.2 ml PBS, 0.1% Tween 20. 100 µl of beads were used for Hel2-HTP samples, 150 µl of beads were used for New1-HTP and untagged samples. Magnetic IgG beads (prepared as described above) were washed twice prior to immobilization with 2 bead volumes of lysis buffer. RNase digestion was performed after on-bead tobacco etch virus protease (TEV) cleavage (for elution from IgG beads) in 730 µl volume of TEV cleavage solution with 1:100 diluted RNaceIT for 5 min (1.3 µl RNaceIT: all 30°C samples), 4.5 min (1 µl RNaceIT: 20°C, New1-HTP samples and untagged controls), or 4 min (1 µl RNaceIT: 20°C, Hel2-HTP samples). For 20°C samples, additional changes were applied: Following sodium dodecyl sulfate gel electrophoresis on NuPAGE^TM^ Bis–Tris 4%–12% gels, instead of transferring protein complexes from gel to a membrane, gel pieces were excised, and protease digestion was performed with crushed gel pieces, pooling several samples in one tube. For all samples, reverse transcription (RT) was performed with Superscript III, with indexed RT primer (30°C samples) or non-indexed RT primer (20°C samples). For 20°C samples, RNA:cDNA hybrids after RT were additionally subjected to RNaseH cleavage for 30 min at 37°C, whereas RTs with indexed RT primer (30°C samples) were treated with Exonuclease I. The products of polymerase chain reaction (PCR) reactions (performed with LATaq and PCR primers as mentioned in Supplementary Table S2) were either purified with Qiagen PCR purification kit (30°C samples) or remaining primers were removed from PCR products by digestion with Exonuclease I (20°C samples; adding 1–1.5 µl Exonuclease I to 150 µl PCR mixture) and DNA was phenol extracted and ethanol precipitated, instead of kit-based purification. Then, 20°C sample PCR products were size-selected on Tris-Borate-ethylenediaminetetraacetic acid (TBE) polyacrylamide gels and eluted in milliQ water and ethanol precipitated, whereas 30°C samples were size selected on Metaphor agarose (Lonza), as previously described [21]. All samples (30°C, two biological replicates, one sequencing run per replicate; 20°C, two biological replicates, one sequencing run per replicate) were sequenced on a MiniSeq system (Illumina), using 75 bp single-end sequencing. For 30°C samples, index reads were also sequenced. Barcodes are given in Supplementary Table S9.

### Sample preparation for western blot and northern blot

For western and northern blot experiments, cultures (25 ml) were grown at 20°C and 220 rpm shaking in synthetic defined (SD) drop out medium corresponding to the respective selection marker for plasmid-containing strains, or in SD complete media. Secondary cultures were inoculated at OD_600_ 0.05 (10 ml) and grown until log phase, reaching an OD_600_ of 0.5–0.9, harvested by centrifugation, including an additional washing step with ultrapure water. For western blot, pellets were stored at −20°C and lysed by a chemical lysis approach [68], including an additional centrifugation step (18,620 × *g*, 5 min) before loading 10 µl of the lysate. For northen blot pellets were resuspended in 750 µl TRIzol™ Reagent, snap frozen in liquid nitrogen and stored at −80°C. Further details of sample processing are given in Supplementary Methods.

### Long-read sequencing

Strains were grown, harvested and RNA was prepared as described before (see the “northern blot” section), with the exception, that cultures were grown in SD complete media. After measuring RNA concentration, RNA was stored at −80°C. For strains lacking *SKI2*, end healing was conducted with T4 polynucleotide kinase (PNK) to remove 2′,3′-cyclic phosphate ends (which are the likely product of NGD mediated endonucleolytic cleavage [7]). For this, total RNA was used in a reaction volume of 60 µl total (1× T4 PNK reaction buffer B, 12 µg RNA, 30 U T4 PNK) and incubated for 30 min at 37°C. Afterwards, acidic phenol/chloroform extraction was performed, followed by precipitation as described before (see the “northern blot” section in Supplementary Methods). Next, poly(A) tailing was conducted according to the manual “Poly(A) Tailing of RNA using *E. coli* Poly(A) Polymerase” (NEB #M0276). After 30 min of incubation at 37°C, ethylenediaminetetraacetic acid was added to stop the reaction and RNA clean-up was performed with RNAClean XP beads at a concentration of 2×. Library preparation and sequencing was performed as described previously [69, 70]. An overview of libraries is given in Supplementary Table S10. Samples were sequenced in (biological) duplicate or triplicate per condition.

### Mass spectrometry

#### Growth and lysis

Cultures were grown (20 ml) and harvested as described before (see “Long-read sequencing” section). After harvesting, 150 µl radioimmunoprecipitation assay (RIPA) buffer and 150 µl zirconia beads were added to each cell pellet, vortexed at 3,000 rpm for 30 s for a total of ten rounds, cooling on ice for at least 30 s per round. Supernatant was transferred to a fresh tube and centrifuged at 18,620 × *g* at 4°C for 10 min, then transferred to a fresh tube and sonicated in an ice cold sonicator water bath (Sonorex Super RK 31) for 5 min to shear genomic DNA. The protein concentration was measured with ROTI^®^Quant universal according to manufacturer’s protocol, using the plate reader FlexStation (Molecular Devices).

#### Enzymatic protein digestion

All samples were processed using the SP3 approach [71]. The proteins were then digested using trypsin overnight at 37°C. The resultant peptide solution was purified by solid phase extraction in C18 StageTips [72].

#### Liquid chromatography tandem mass spectrometry

Peptides were separated via an in-house packed 45-cm analytical column (inner diameter: 75 μm; ReproSil-Pur 120 C18-AQ 1.9-μm silica particles, Dr Maisch GmbH) on a Vanquish Neo UHPLC system (Thermo Fisher Scientific). The online reversed-phase chromatography separation was conducted through a 100-min nonlinear gradient of 1.6%–32% acetonitrile in 0.1% formic acid at a nanoflow rate of 300 nl/min. The eluted peptides were sprayed directly by electrospray ionization into an Orbitrap Astral mass spectrometer (Thermo Fisher Scientific). Mass spectrometry (MS) was conducted in data-dependent acquisition mode using a top 50 method with one full scan in the Orbitrap analyzer (scan range: 325–1300 m/z; resolution: 120 000, target value: 3 × 10^6^, maximum injection time: 20 ms) followed by 50 fragment scans in the Astral analyzer via higher energy collision dissociation (HCD; normalized collision energy: 26%, scan range: 150–2000 m/z, target value: 1 × 10^4^, maximum injection time: 5 ms, isolation window: 1.4 m/z). Precursor ions of unassigned, +1 or higher than +6 charge state were rejected. Additionally, precursor ions already isolated for fragmentation were dynamically excluded for 20 s.

### Computational analysis

#### Mass spectrometry data processing

Raw data files were processed by MaxQuant software (version 2.1.3.0) [73] using its built-in Andromeda search engine [74]. MS/MS spectra were searched against a target-decoy database containing the forward and reverse protein sequences of UniProt *S. cerevisiae* reference proteome (release 2023_05; 6091 entries) and a default list of common contaminants. Trypsin/P specificity was assigned. Carbamidomethylation of cysteine was set as fixed modification. Methionine oxidation and protein N-terminal acetylation were chosen as variable modifications. A maximum of two missed cleavages were allowed. The “second peptides” options were switched on. “Match between runs” was activated. The minimum peptide length was set to 7 amino acids. False discovery rate was set to 1% at both peptide and protein levels. For label-free quantification (LFQ) of proteins, the MaxLFQ algorithm [75] was employed using its default normalization option. Minimum LFQ ratio count was set to one. Both the unique and razor peptides were used for quantification. Differential expression analysis was performed in R statistical environment. Reverse hits, potential contaminants and “only identified by site” protein groups were first filtered out. Proteins were further filtered to retain only those detected in all three replicates in either the wildtype or knockout group. Following imputation of the missing LFQ intensity values, a linear model was fitted using the limma package [76] to assess the difference between the wildtype and knockout groups for each protein, with adjustment for multiple testing using the Benjamini–Hochberg approach [77].

#### CRAC data analysis

CRAC data were analyzed, primarily employing the pyCRAC package [78] as previously described [21] for analysis of total hit distributions, tRNA hit distributions, rRNA read and single nucleotide deletion coverage and plotting of mRNA metagene plots (with deeptools [79]; mRNA metagene analysis was done as previously described for metagene plots that were scaled over part of the gene body [21]), always without scaling to library size. For 3′-untranslated region (3′-UTR) binding analysis, deeptools [79] module computeMatrix was used on the same BIGWIG files used for analysis of mRNA coverage, but employing a reference GTF file containing 3′-UTR coordinates instead of open reading frames (ORF) coordinates, and using the 100 nt upstream and downstream unscaled option, 10 nt bins, and ‘gene-body’ size 50 (thus five bins, representing the annotated 3′-UTR). Resulting values for the 5 bins of each individual 3′-UTR were summed up for each sample, and a pseudo-count of 1 was added, to avoid division by zero for cases where no reads were obtained for a 3′-UTR in one of the samples. Then, the ratio of 3′-UTR-sums was calculated for each pair of Hel2-HTP, *new1∆*, and Hel2-HTP, wildtype sample (replicates 1 and 2 at 30°C, and replicates 1 and 2 at 20°C). For pairs where no 3′-UTR reads were recovered in either sample, the value was set to N/A. Ratios were then normalized to the median of all ratios calculated for a pair of samples, to avoid the influence of factors that affected mRNA read values (e.g. higher or lower recovery of rRNA *versus* mRNA, leading to higher or lower reads per million values for mRNA, including 3′-UTR in some samples). Data were then plotted using boxplotR [80], separating mRNAs into groups according to either the C-terminally encoded amino acid, the C-terminal codon, or by whether or not they were collision-prone (‘strong’, ‘mild’, ‘not’) and/or targeted by NGD (‘strong NGD’, ‘strong other’).

#### Stop codon context analysis

The sequence surrounding the stop codon, including the stop codon itself of all ORFs of all coding sequences (CDS) (excluding ORFs classified as “dubious” or “pseudogene”) were extracted from “orf_coding.fasta”, downloaded from SGD (http://sgd-archive.yeastgenome.org/sequence/S288C_reference/orf_dna/; accessed on 22 July 2024) and used for various analyses, unless mentioned otherwise.

#### Analysis of long-read sequencing (via Oxford Nanopore Technologies native barcoding)

To explore endonucleolytic cleavage sites, a pipeline was established to extract read ends from long-read sequencing data. Based on FASTQ output files from the Oxford Nanopore MinKnow sequencing software, sequences were subjected to adapter trimming (porechop_abi –no_split –extra_–end_trim 0 –min_trim_size 8 –end_treshold 85) [81]. For datasets which were used to quantify transcript abundance (in the presence of Ski2 and Xrn1), poly(A) tail trimming was carried out using cutadapt [82] with the “–poly-a” option. Reads were aligned to the *S. cerevisiae* reference genome (Saccharomyces_cerevisiae.R64-1) using minimap2 (minimap2 -G3000 –MD -ax map-ont -t 40) [83]. Alignments were filtered using the SAMtools option (-F 3844) to exclude unmapped reads, low-quality and nonprimary alignments, duplicates, and supplementary alignments. Prior to read end analysis, the BAM files were converted into BED files using BEDtools [84, 85]. According to the reference genome, the corresponding annotation file from SGD (https://downloads.yeastgenome.org/latest/saccharomyces_cerevisiae.gff.gz; accessed on 15 May 2024; also available from the authors upon request) was used to generate a BED gene reference file containing all coding sequences (available on GitHub https://github.com/WinzLab/Yeast-elongation-factor-homolog-New1-protects-a-subset-of-mRNAs-from-degradation-by-no-go-decay) [86]. For analysis of endonucleolytic fragments, coverage and read ends within the last 300 nt of the coding region (including the stop codon) were extracted. To capture the relevant read ends, different read ends were analyzed depending on the genetic background. In *SKI2* deletion strains, where 5′-fragments resulting from endonucleolytic cleavage are stabilized, 3′-termini of those fragments were obtained by extracting the 3′-ends of reads aligned in the sense orientation (resulting from second strand cDNA) and the 5′-ends of reads aligned in the antisense orientation (from first strand cDNA). Conversely, in strains lacking *XRN1*, where 3′-fragments accumulate, the 5′-ends of those fragments were obtained by extracting the 5′-ends of reads aligned in the sense orientation (from second strand cDNA) and the 3′-ends of reads aligned in the antisense orientation (from first strand cDNA). This was necessary as both, first and second strand of cDNAs were sequenced.

Using PyRanges [87], only read ends falling within the last 300 nt of each gene’s coding sequence were retained. For each gene, coverage of the start codon, stop codon and the CDS was calculated using BEDTools [84, 85]. The number of read ends per position was then normalized to the coverage at the start codon in strains lacking *SKI2* or at the stop codon in strains lacking *XRN1*, which represents an approximation of the total number of mRNAs for each gene, including both, full-length (FL) transcripts and cleavage fragments. To find whether more read ends, belonging to endonucleolytic fragments, were generated in the absence of New1, we introduced a cleavage score, which was applied to each mRNA. To calculate this cleavage score for each mRNA, normalized read end counts from a *new1Δ* sample were divided by the sum of counts from its matched control sample (which differed only by the presence of the *NEW1* gene), while accounting for additional genetic backgrounds (e.g. *ski2Δ, xrn1Δ*, or additional deletions of RQT or NGD factors). The documentation of the pipeline and code is available on Github (https://github.com/WinzLab/Yeast-elongation-factor-homolog-New1-protects-a-subset-of-mRNAs-from-degradation-by-no-go-decay) and archived in Zenodo [86].

To identify genes targeted by endonucleolytic cleavage, while minimizing background noise, we used the following three criteria: (i) average cleavage score over all replicates per pair of strains ≥0.75, (ii) at least 10 read ends occurred within the 300 nt window in *NEW1*-*SKI2*–deficient strains and at least 15 reads in *NEW1*-*XRN1*–deficient strains (average), and (iii) these read end counts represented ≥20% of the coverage at the start codon in the *ski2Δ* background or at the stop codon in the *xrn1Δ* background, respectively, meaning that at least ∼20% of reads aligned to a gene represented cleavage fragments.

#### Computing the coverage along the last 300 nt

BEDTools was used to compute the coverage along the last 300 nt upstream of the 3′-UTR per position, based on the same gene list used for read end analysis (“compute_last_300nt_coverage.py” provided on GitHub as mentioned above).

#### Generation of metaplots for long-read sequencing coverages and read ends for NGD targets

To generate metaplots for nanopore long-read sequencing coverages and read ends, mRNAs identified as NGD targets in *new1Δ* background (without additional deletion of RQT components), or mRNAs identified as targets in *new1Δ* background with additional deletion of one or more RQT components, or the top 10% most abundant mRNAs of the ‘strongly affected’ group (as determined by CDS coverage in the *new1Δ, ski2Δ* background, or in the *new1Δ, xrn1Δ* background), but not identified as NGD target via the analysis described above, were analyzed. For the *SKI2*-deletion and *XRN1*-deletion backgrounds, only targets identified in the respective background were taken into account. For coverage metaplots, for each mRNA, the minimal sequencing depth was set to 0 by subtracting the actual value for minimal sequencing depth of the respective mRNA from all values. Following this, all values were divided by the difference between highest and lowest value (amplitude) over the 300 nt region, thereby setting this amplitude to 1. Then, averages were calculated over all mRNAs for each sample, and consecutively, averages were calculated over all replicates for each strain background, or additionally over all different samples containing an additional deletion of a gene encoding a member of the RQT complex. Before plotting, maxima were again set to 1 and minima to 0, to ease comparison between different samples.

For read end metaplots, for each mRNA, all read end values were divided by the maximum value, thereby setting the maximum for each mRNA to 1, then averages were calculated as described for coverage metaplots, additionally also calculating an average over all samples in which an NGD factor was deleted. Before plotting, instead of setting maxima to 1 and minima to 0, average values were multiplied with the average total number of read ends within the last 300 nt, calculated for all mRNAs from all replicates for each strain or group of strains.

#### 5PSeq analysis

Published 5PSeq WIG files [64] for wildtype and *new1∆* were downloaded and 5′-coordinates were extracted by using BEDTools [84, 85] intersect and a modified gene list which defines the last 300 nt of each gene, normalized as counts per million, pooled, and relative values per position were calculated as described above.

#### Generation of metaplots (5PSeq)

To generate metaplots of 5PSeq read ends within the last 300 nt of mRNA coding sequences, relative values per position were summed for each group of mRNAs (all strongly affected, mildly affected, unaffected; strongly affected NGD targets identified in *new1Δ* background; strongly affected NGD targets only identified in *new1Δ,RQT-KO* background; strongly affected other). To allow comparison between different groups, summed values were then divided by the number of mRNAs in each category. Optionally, data were smoothed by applying a 5 nt rolling-window average filter.

#### RiboSeq analysis

Queuing scores of the published RiboSeq data for wildtype and *new1∆* strains [63] were computed according to Kasari *et al.*, 2019 (https://github.com/GCA-VH-lab/2019-NAR) [63].

#### Readthrough analysis of 5PSeq and RiboSeq

5′-coordinates of the 5PSeq WIG files [64] for wildtype and *new1∆* were extracted within a window of 200 nucleotides upstream and downstream of the stop codon. Extracted values were normalized to counts per million, pooled, and further normalized to the total number of extracted 5′-coordinates per gene.

For RiboSeq, 20°C *new1∆* and wildtype raw data from Kasari *et al.* 2019 were reprocessed following the described pipeline [63] to reproduce P-site coordinates, which were subsequently analyzed in the same way as the 5PSeq data.

For each gene parallel analysis of RNA structure (PARS) scores [88] were extracted along the CDS and within the last 300 nt upstream of the 3′-UTR based on the corresponding annotation file [88]. Confidence intervals were determined via Microsoft excel choosing a confidence level of 95%.

PARS score analysis

#### Generation of boxplots

Boxplots were generated using the boxplotR tool [80] with default settings, except for showing data points (jittered), and, in some cases, variable width boxes, logarithmic scaling of y-axis, and adjusting the plot size. For all boxplots center lines show the medians, box limits indicate the 25^th^ and 75^th^ percentiles, as determined by R software; whiskers extend 1.5 times the interquartile range from the 25^th^ and 75^th^ percentiles; 20°C samples are usually shown with blue boxes, 30°C samples with red boxes. When other color codes apply, they are described in the caption.

#### Generation of Venn diagrams

Venn diagrams were generated using InteractiVenn tool [89].

#### Generation of Sequence logos

Sequence logos were generated using WebLogo 3 [90, 91] (https://weblogo.threeplusone.com/; accessed in September 2025).

### Western and northern blot analysis

Western and northern blot signals were quantified with ImageJ [92]. Rectangle width was adjusted to average signal size and height of the membrane. Tangents were fitted to the signal specific graphs to determine the area under the curve. Based on the loading control (*TDH3* mRNA for northern blots; α-Tubulin for western blots), a normalization factor was computed to adjust the signal of interest and to determine the relative fold change, as well as the log_2_ fold change.

### Spot assay

Cultures were grown at 20°C with shaking at 220 rpm in yeast extract peptone dextrose (YPD) media. Serial dilutions were prepared starting from an OD_600_ of 1, followed by four consecutive 1:10 dilutions and 10 µl of each dilution were spotted on YPD plates. Plates were incubated at either 20°C or 30°C (control) and imaged after 3 days.

### Statistical analysis

Unless mentioned otherwise, Student’s t-test was performed via Microsoft excel, choosing two tailed distributions with an unequal sample variance (heteroscedastic).

## Results

### The absence of New1 causes recruitment of Hel2 to mRNA 3′-UTRs, dependent on the C-terminal codon

**Figure 1.**
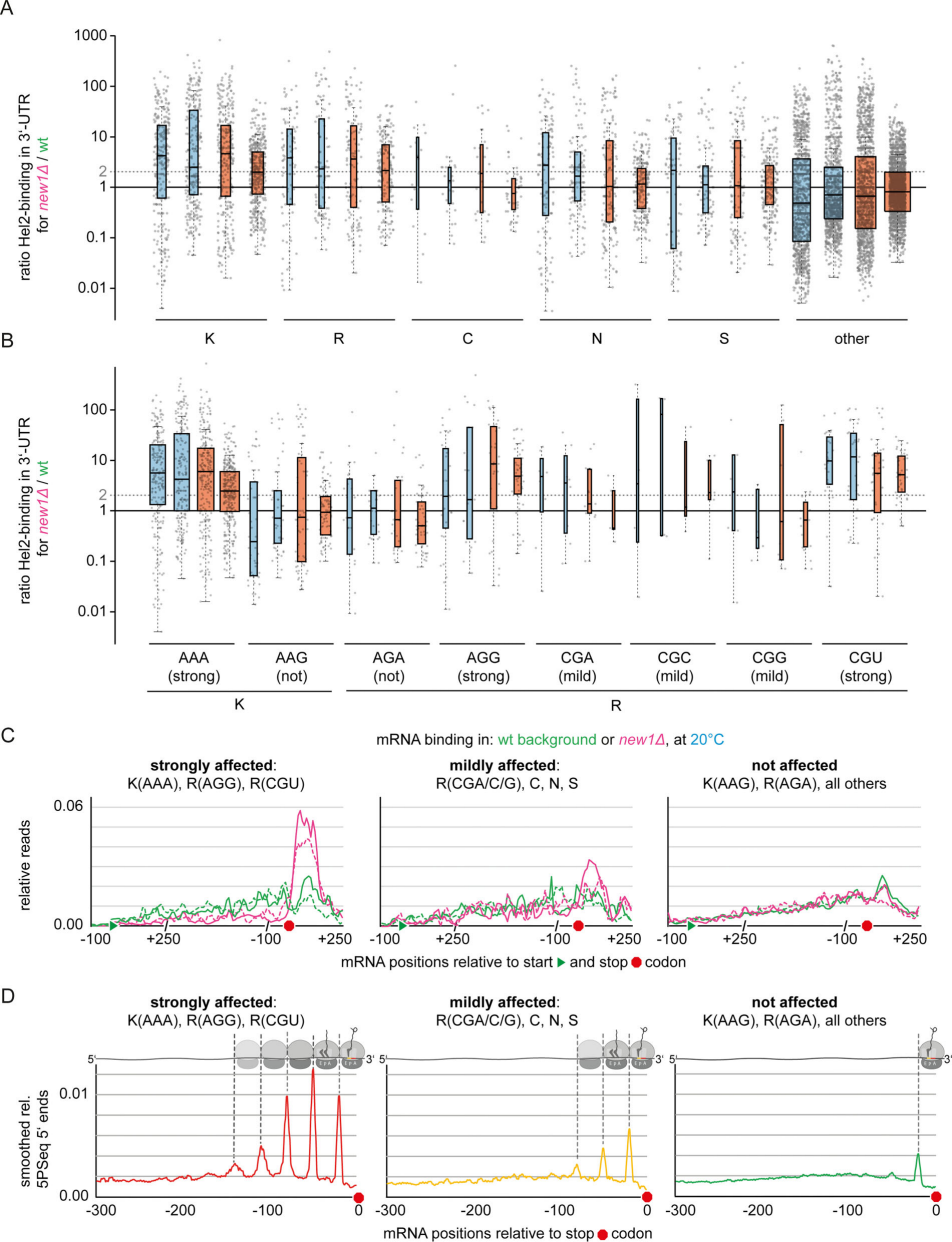
Ratio of Hel2 binding in 3′-UTR for *new1Δ*/wildtype at 20°C (blue, two biological replicates), and 30°C (red, two biological replicates) **(A)** for mRNAs encoding C-terminal amino acids lysine (K), arginine (R), cysteine (C), asparagine (N), serine (S), or other (excluding K, R, C, N, S), **(B)** for all lysine and arginine codons. Boxplots (**A,B**): Center lines: medians, box limits: 25^th^ and 75^th^ percentiles, whiskers: 1.5× interquartile range from 25^th^ to 75^th^ percentile. All data points are represented by dots. Width of the boxes is proportional to the square root of the sample size. **(C)** Metaplots showing Hel2 binding (relative hits) in either wildtype (wt, green, 2 replicates) or *new1Δ* (magenta, two replicates) background, at 20°C (analogous plots for analysis at 30°C shown in Supplementary Fig. S3B), for those mRNAs within the group of previously identified top 1,000 highest Hel2-bound mRNAs [21] with C-terminal codons being either strongly affected, mildly affected, or not affected by lack of New1. Green triangle indicates the position of the start codon and red octagon indicates the position of the stop codon. **(D)** Re-analyzed published 5PSeq data [64], showing smoothed metaplots of normalized, relative 5PSeq 5′-ends in *new1Δ*, compared to wildtype for strongly affected (left), mildly affected (middle) and unaffected (right) mRNAs. Corresponding ribosome footprints are indicated above each plot.

Since absence of New1 was reported to cause ribosome queuing on mRNAs encoding specific C-terminal amino acids, we wondered whether these queues are equivalent to collided ribosomes and might elicit collision-specific downstream responses, including NGD. This does not need to be the case, as ribosomes can occur as collided di- and higher-order polysomes without eliciting quality control [93–96]. We thus tested if the collision sensor Hel2 is recruited preferentially to mRNAs affected by ribosomal queuing in the absence of New1, using the crosslinking and immunoprecipitation (CLIP)-like approach CRAC [67], in either wildtype or *new1Δ* strains. Yeast were cultured at 30°C and 20°C, since lack of New1 increases cold sensitivity [65]. For a subset of mRNAs, Hel2 binding to the 3′-UTR was drastically increased in the absence of New1, at both temperatures (Fig. 1A–C, and Supplementary Figs S2 and S3). This affected mRNAs encoding C-terminal lysine (K) or arginine (R) and to a lesser extent cysteine (C), asparagine (N), or serine (S), but not other amino acids (Fig. 1A and Supplementary Fig. S2), in agreement with queues specifically on such mRNAs [63, 64]. Only specific lysine and arginine codons mediated this effect, the most strongly affected being K(AAA), R(AGG), and R(CGU), but not, e.g. K(AAG) or R(AGA) (Fig. 1B and C). For the mildly affected cysteine, asparagine, and serine, the codon specificity appeared less pronounced but was also more difficult to judge due to lower numbers of observations, especially for cysteine (Supplementary Fig. S3A). For C-terminal asparagine codons, the increase in Hel2 recruitment to 3′-UTRs was more pronounced at 20°C than 30°C, in contrast to all other codons (Fig. 1A, and Supplementary Figs S2 and S3A). Our findings are in agreement with a recent publication [64] showing ribosomal queuing for specific C-terminal lysine, arginine, asparagine and serine codons. We therefore conclude that queues indeed represent ribosome collisions that cause recruitment of collision sensor Hel2. For all following analyses, codons K(AAA), R(AGG), and R(CGU) and mRNAs ending in them, will be referred to as ‘strongly affected’ or ‘strong’, all cysteine (C), asparagine (N), and serine (S) codons, as well as arginine codons R(CGA), R(CGC), and R(CGG) and mRNAs ending in them, will be referred to as ‘mildly affected’ or ‘mild’, and all remaining codons and mRNAs ending in them, as ‘not affected’ or ‘not’. In the following, unless mentioned otherwise, experiments were conducted at 20°C, as reported phenotypes [63–65] were stronger at that temperature. We further validated the three groups (strong, mild, not) against published 5PSeq data [64] and generated metaplots for the abundance of 5′-ends within the last 300 nt upstream the 3′-UTR in *new1Δ* strains (Fig. 1D). Strongly affected sequences revealed a strong queuing signature, with signals consistent with terminating ribosomes, as well as collided di-, tri-, tetra-, and pentasomes (Fig. 1D). In contrast, mildly affected mRNAs showed comparably weaker signals, consistent with up to collided trisomes. Unaffected mRNAs only exhibited slightly elevated 5′-end counts near the stop codon, corresponding to the terminating ribosome. The strength of queuing in the absence of New1 thus correlates with recruitment of Hel2.

#### Hel2 recruitment to mRNA 3′-UTRs upon lack of New1 is not generally due to stop codon readthrough

Next, we asked why Hel2 binds 3′-UTRs. We argued that this could be due to stop codon readthrough, as reported for lack of eEF3 [60], which could lead to the need of translation quality control, e.g. due to ribosome stalling on poly(A) and other unfavorable sequences [97, 98]. Unfavorable sequences would be more likely to be present in 3′-UTRs compared to coding sequences, due to lack of evolutionary pressure for codon optimization in UTRs. Ribosome densities had been shown to be slightly increased in 3′-UTRs of mRNAs affected by the lack of New1, but stop codon readthrough had previously not been observed using a dual-luciferase readthrough reporter system [63]. However, that reporter construct had only been tested in the ‘in-frame’ reading frame. Since ribosome collisions can cause frameshifting [15], we argued that readthrough itself might be caused by frameshifting, as this would lead to part of the stop codon being recognized as a sense codon. In addition, eEF3, the homologue of New1 has been shown to be actively involved in frameshifting at collisions [99]. As a consequence, readthrough could occur in a different reading frame. We thus designed a readthrough reporter, to assess potential readthrough in all reading frames (Supplementary Text S1, Supplementary Fig. S4), but did not observe any significant differences in readthrough between a reporter ending with the strongly affected codon K(AAA), compared to one ending with the unaffected codon K(AAG), both encoding lysine, nor did we observe differences between readthrough in *new1Δ* or wildtype strains. While writing our initial manuscript, a report was published [64] showing that readthrough may occur in the absence of New1, but only under very specific conditions, where the affected C-terminal codon is followed by the weak stop codon ‘UGA’ and an additional nucleotide ‘C’, but not for other combinations. However, this sequence pattern occurs in only 20 out of the >600 strongly affected mRNAs, and those 20 mRNAs do not exhibit higher Hel2 recruitment to 3′-UTRs compared to others (Supplementary Fig. S5, discussed in-depth in Supplementary Text S1). We also re-analyzed the previously published RiboSeq [63] (Supplementary Fig. S6, S7) and 5PSeq data [64] (Supplementary Fig. S8) and did not find clear evidence for generalized stop codon readthrough in 3′-UTRs of strongly affected mRNAs as the major cause of Hel2 binding in these regions; however, considering resemblances to known stop codon readthrough scenarios [100, 101], we also cannot fully exclude this possibility (see also Supplementary Text S1). Taken together, we conclude that ribosome collisions upstream of stop codons do not generally lead to stop codon readthrough, and that Hel2 mRNA binding in 3′-UTRs is more likely not due to translation in this region, which could also be caused by re-initiation, as previously observed in similar contexts [93]. Instead, we favor the hypothesis that Hel2 crosslinking in 3′-UTRs in our system is mainly driven by Hel2 binding to collided ribosomes at the stop codon, and making contacts with downstream 3′-UTR. We further discuss additional hints for this in Supplementary Text S1 and Supplementary Fig. S9.

### Hel2 recruitment upon lack of New1 causes degradation of affected mRNAs by NGD

**Figure 2.**
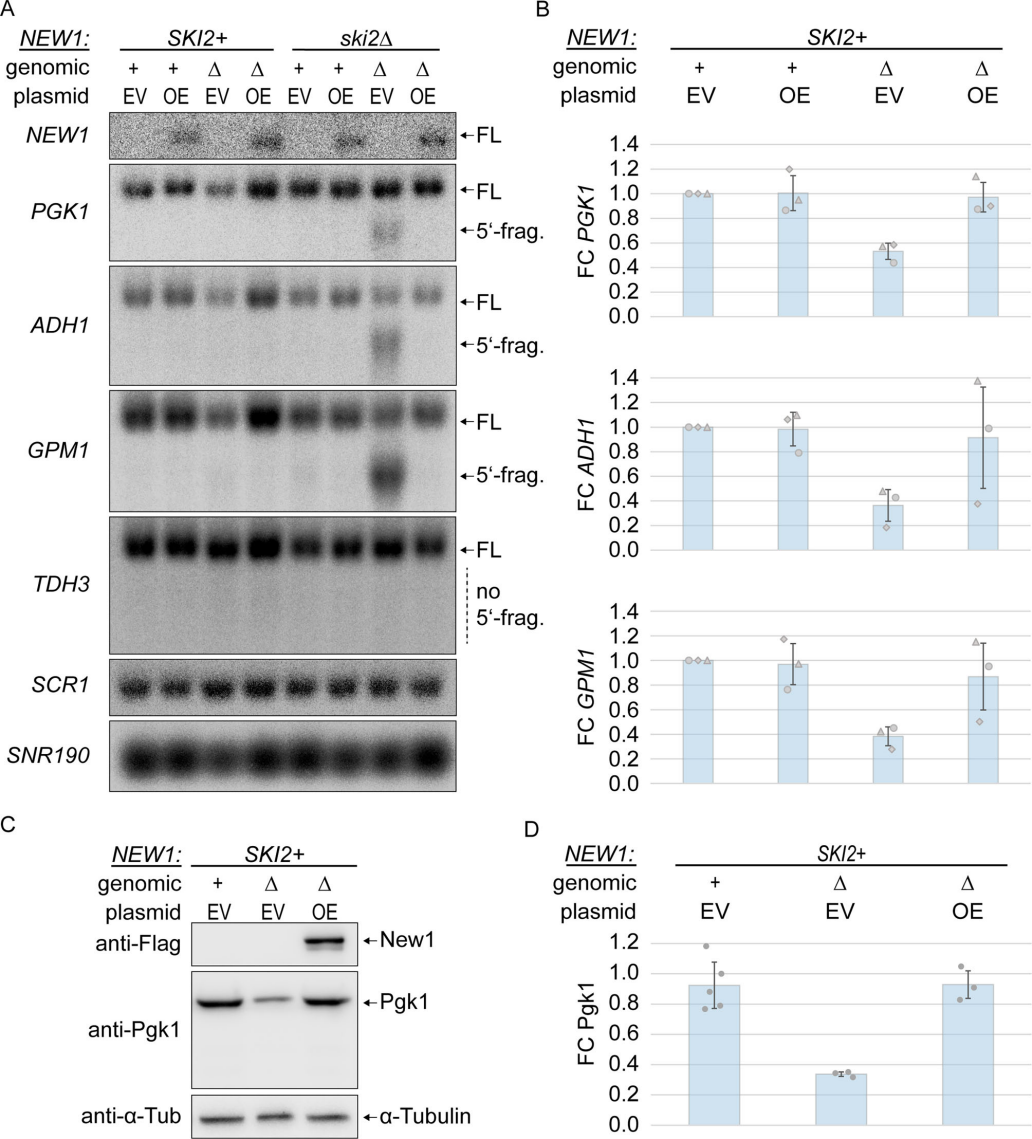
**(A)** Northern blot analysis of FL mRNA levels or endonucleolytic 5′-fragment (5′-frag.) for *NEW1-FLAG, PGK1, ADH1, GPM1, TDH3, SCR1*, and *SNR190* in *new1Δ* (genomic Δ), *NEW1*-positive (genomic +) strains, containing an empty vector (EV) (plasmid EV) or New1-FLAG overexpression (OE) vector (plasmid OE), and containing or lacking the *SKI2* gene. Note that endogenous *NEW1* mRNA is not detected, as a probe against FLAG was used to visualize *NEW1*-*FLAG* mRNA. **(B)** Quantification of FL mRNA levels for *PGK1, ADH1* and *GPM1*, relative to *TDH3* (control), compared to wildtype strains with empty vector (genomic +, plasmid EV), related to panel (A), n = 3 biological replicates. Data were normalized to *SCR1* signals. **(C)** Western blot analysis of Pgk1 levels in *new1Δ* (genomic Δ, plasmid EV), and New1-overexpressing (genomic Δ, plasmid OE) strains, compared to wildtype strains containing an empty vector (genomic +, plasmid EV). **(D)** Quantification of protein levels for Pgk1, relative to α-Tubulin, related to panel (C), *n* ≥ 3 biological replicates. Data were normalized to α-Tubulin signals. Error bars in panels (B) and (D) represent 1 standard deviation. Additional replicates and quantification shown in Supplementary Fig. S10.

One possible downstream consequence of Hel2-catalyzed ribosomal protein polyubiquitination is NGD. We therefore asked whether mRNAs that exhibit increased ribosomal queuing and increased Hel2 recruitment upon deletion of *NEW1* are targeted by NGD. Canonical NGD involves endonucleolytic cleavage by Cue2, followed by exonucleolytic degradation of cleavage fragments. We decided to probe for both, total mRNA levels and endonucleolytic cleavage fragments. To enable detection of cleavage fragments by northern blot, we used strains lacking Ski2, a member of the Ski complex, which is a necessary co-factor for the cytosolic RNA exosome [102]. As a result, the 5′-fragment resulting from endonucleolytic cleavage is stabilized [4], whereas the 3′-fragment is still degraded by Xrn1. As potentially affected target mRNAs, we chose the highly expressed *PGK1, ADH1*, and *GPM1* transcripts, all ending on the K(AAA) codon and exhibiting both, ribosomal queuing and Hel2 3′-UTR recruitment. These mRNAs all belong to the top seven most highly expressed, strongly affected mRNAs, according to our recent nanopore sequencing data [69], and were deemed well-suited for northern blotting due to sufficiently long, yet different size. All three genes encode important metabolic enzymes, involved in glycolysis/gluconeogenesis (*PGK1* and *GPM1*), or alcoholic fermentation (*ADH1*). As a control, we visualized mRNA for another metabolic gene: *TDH3*, a traditional house-keeping gene which ends on the unaffected alanine (A) amino acid, encoded by the A(GCU) codon. We found that lack of New1 leads to a strong decrease in total mRNA levels (in *SKI2 *+ background) of all three affected mRNAs by ∼40%–60%, but not the unaffected *TDH3* mRNA (Fig. 2A and Supplementary Fig. S10A and B; comparing lanes 1,2 to lane 3; quantification Fig. 2B). This effect is fully rescued by plasmid-based overexpression of C-terminally FLAG tagged New1 (Fig. 2A, lane 4, and Fig. 2B). In the absence of Ski2 and New1, but not Ski2 alone, we observed the appearance of shorter fragments, which we interpret as endonucleolytic 5′-fragments, for all three affected mRNAs, but not the control mRNA of *TDH3*. Endonucleolytic cleavage was suppressed by overexpression of FLAG tagged New1 (Fig. 2A, lanes 5–8). For Pgk1, we further tested whether lower mRNA levels translate to decreased protein levels in affected strains. Here, we indeed observed a decrease in Pgk1 protein by ∼63%, but not in α-Tubulin, which ends with the unaffected amino acid phenylalanine (F), encoded by F(UUU): Tub1; or F(UUC): Tub3 (Fig. 2C and D). We thus showed for several examples that, in the absence of New1, mRNAs ending with strongly affected codons [here: K(AAA)] are cleaved to form shorter fragments, leading to decreased FL mRNA levels, supporting the notion that they become targets of NGD. Additionally, our data on Pgk1 suggest that levels of the encoded proteins are also decreased.

#### NGD of mRNAs upon lack of New1 is initiated by endonucleolytic cleavages upstream of the stop codon

**Figure 3.**
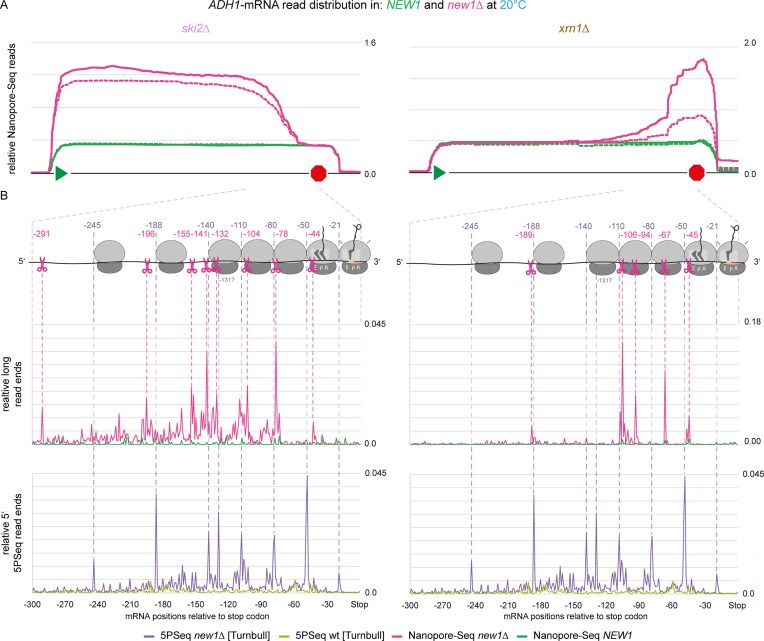
Analysis of endonucleolytic fragments via nanopore sequencing in *new1Δ, ski2Δ* or *new1Δ, xrn1Δ* double knockout *versus ski2Δ* and *xrn1Δ* single knockout yeast strains. **(A)** Comparison of relative nanopore sequencing read distribution along the *ADH1* transcript. Start codon is indicated by green triangle and stop codon by red octagon. *n* = 2 biological replicates, each. **(B)** 3′-end analysis of nanopore sequencing reads from *new1Δ, ski2Δ* double knockout, as well as 5′-end analysis from *new1Δ, xrn1Δ* (magenta, *n* = 2 biological replicates pooled) and *ski2Δ* single knockout (green, *n* = 2 biological replicates pooled) yeast strains for the last 300 nt of the *ADH1* transcript. Data were compared to 5′-ends from 5PSeq reads for *new1Δ* (purple) and wildtype (wt) (light green) representing the position of ribosomes along the mRNA published by Turnbull *et al.* [64]. Ribosome positions are schematically shown, based on 5PSeq 5′-ends, and endonucleolytic cleavage sites are indicated by scissors. A-site of first ribosome is at pos. −1 to −3 (stop codon).

To obtain high-resolution data on cleavage sites, we performed long-read nanopore sequencing (direct cDNA). In our *SKI2*-deletion strains, the 5′-fragment is stabilized. This fragment would lack the poly(A) tail of the FL mRNA (Supplementary Fig. S11). Standard direct cDNA library preparation for nanopore sequencing relies on the use of poly(dT) primers to perform first-strand synthesis. Thus, we employed *in vitro* polyadenylation to add poly(A) tails also to non-polyadenylated species. However, endonucleolytic cleavage in NGD had been reported to yield 2′,3′-cyclic phosphates at the 3′-terminus of the 5′-fragment [7], which inhibit polyadenylation. We therefore dephosphorylated RNA with PNK, prior to *in vitro* polyadenylation. In addition, to also sequence 3′-fragments, we generated *xrn1Δ* strains, in which the 3′-fragment is stabilized. Since 3′-fragments naturally contain poly(A) tails, here, we performed standard sequencing library preparations. We then compared sequencing data for *new1Δ, ski2Δ* double knockout to *ski2Δ* single knockout, and for *new1Δ, xrn1Δ* double knockout to *xrn1Δ* single knockout. As a first analysis, we plotted sequence coverage for one of our NGD candidates: *ADH1*. Here, we observed the appearance of shorter species only for the double knockout conditions, but not the single knockouts, representing the expected 5′-fragments in *new1Δ, ski2Δ* and 3′-fragments in *new1Δ, xrn1Δ* (Fig. 3A). We then also mapped 3′-ends of 5′-fragments, as well as 5′-ends of 3′-fragments, to pinpoint possible Cue2 cleavage sites (Fig. 3B). For *ADH1*, we observed very heterogeneous fragment lengths, suggesting multiple cleavage sites, between ∼40 and ∼300 nt upstream of the stop codon (Fig. 3B). This size range is similar to previous observations made on stalling reporters [1, 47] and gene-internal poly(A) [103], and fragment lengths are also in good agreement with previous reports on both RQC-coupled and RQC-uncoupled NGD [47]. An ∼30 nt periodicity (Fig. 3B) suggests, that these cleavages result from multiple collisions, also reminiscent of previous reporter data [1]. This also matches the published 5PSeq data for *new1Δ* and wildtype strains [64], suggesting the existence of heterogeneous length collisions of at least up to pentamers (Fig. 3B, bottom). For the *ADH1* cleavage fragments, read ends of 5′-fragments and 3′-fragments do not fully match each other but are in similar ranges. However, for 5′-fragments, we find additional cleavage sites, further apart from the stop codon, e.g. at −155 and −291 nt, which do not have matching 3′-fragments.

There are different possible explanations for this: (i) it is possible that these cleavages are due to collisions that are part of the original queue at the affected C-terminal codons. In the presence of ribosomal splitting activity by the RQT complex, the initial cleavages preferentially occur at or close to the original, stalled ribosome. This first cleavage would generate a 3′- and a 5′-fragment both captured by our methodology. However, additional cleavages further within the coding sequence would generate 3′-fragments that lack poly(A), and would thus not be captured by our 3′-fragment sequencing approach. Conversely, further shortened 5′-fragments could still be captured via *in vitro* polyadenylation. As the patterns of 5′-fragments we observed closely match the 5PSeq 5′-ends, we believe this hypothesis might be valid. (ii) In addition, we are aware that the 5′-fragment is stabilized in strains that lack Ski2. Hence, translation could be initiated on these fragments, leading to further collisions and additional cleavages. The Cue2-cleaved mRNA would be similar to reporter mRNAs that contain a self-cleaving ribozyme and, post-cleavage, lack both, stop codon and poly(A) tail, but are still translated [7]. Such additional cleavages would, again, only be captured for the 5′-fragments, due to the lack of poly(A) in the newly generated 3′-fragments.

Furthermore, taking into account 5PSeq 5′-termini to approximate (collided) ribosome positions, our data (Fig. 3B) suggest that Cue2-cleavage in the first collided ribosome occurs mainly few nucleotides upstream of the E-site. This is similar to recent findings [47] for RQC-coupled NGD on an exogenous reporter mRNA, whereas cleavages within further trailing ribosomes occur close to the 5′-end of ribosome footprints, similar to what is reported for RQC-uncoupled NGD. In contrast, we hardly observed any cleavage sites within the first, stalled ribosome.

#### Nanopore sequencing reveals multiple NGD targets in New1-lacking yeast

We now aimed to identify additional targets of endonucleolytic cleavage in our sequencing data. Based on observations on *ADH1* (Fig. 3), and after inspection of our other candidates *PGK1* and *GPM1* (sequencing data not shown), we quantified the number of read ends (3′-ends for 5′-fragments, 5′-ends for 3′-fragments) within the last 300 nt including the stop codon, for each mRNA. This count was normalized to read coverage at the start codon (for 3′-fragments) or stop codon (for 5′-fragments), to approximate the proportion of overall transcripts (FL transcripts + cleavage fragments). A ratio of normalized read ends in New1-lacking double knockouts over total read ends in both, the New1-lacking and corresponding New1-containing strain was calculated, which we denominated as cleavage score (Formula 1), where nt denominates the nucleotide position relative to the 3′-nucleotide of the stop codon:


\begin{eqnarray*}
&&\textit{cleavage}\ \textit{score}\\&=& \ \frac{{\mathop \sum \nolimits_{nt = - 300}^{nt = - 1} \textit{norm}.\ \textit{read}\ \textit{ends}\ \left( {new1\Delta } \right)}}{{\mathop \sum \nolimits_{nt = - 300}^{nt = - 1} \textit{norm}.\ \textit{read}\ \textit{ends}\ \left( {new1\Delta } \right) + \ \mathop \sum \nolimits_{nt = - 300}^{nt = - 1} \textit{norm}.\ \textit{read}\ \textit{ends}\ \left( {\textit{control}} \right)\ \ }}\\
\end{eqnarray*}

**Figure 4.**
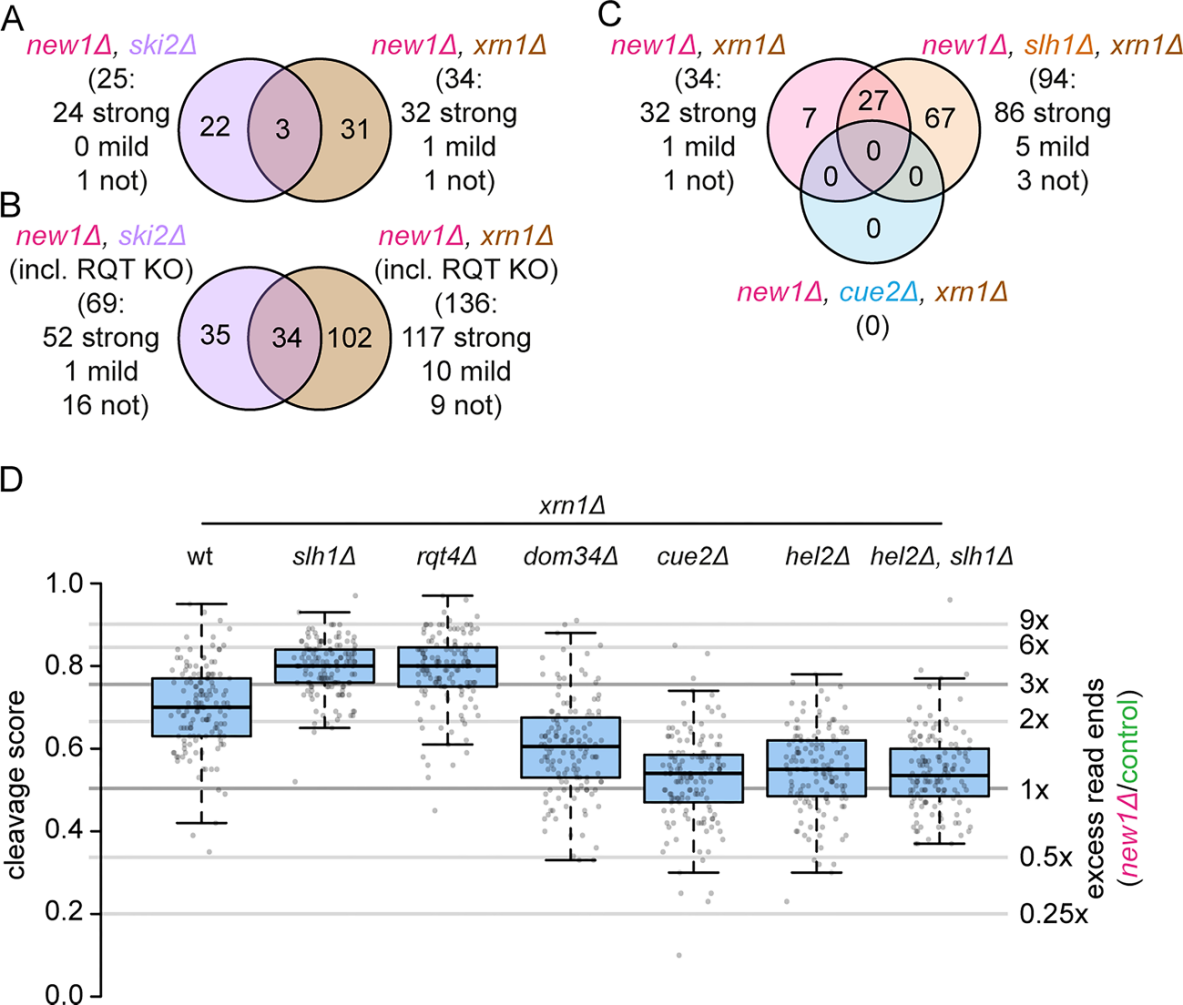
Nanopore sequencing identifies additional NGD targets and supports a canonical NGD mechanism. **(A)** NGD targets identified from sequencing data of double deletants lacking *NEW1* and either *SKI2* or *XRN1*. **(B)** NGD targets identified from data sets including double deletants (see A) and triple and quadruple deletants, additionally lacking *SLH1, RQT4*, and/or *CUE3*. **(C)** Comparison of NGD targets identified in *XRN1*-lacking strains deleted only for *NEW1*, or for *NEW1* and *SLH1*, or (as a control) *NEW1* and *CUE2*. NGD targets are classified by whether they belong to the strongly, mildly, or unaffected group of mRNAs, according to their C-terminal codons. **(D)** Cleavage scores of all NGD targets in *XRN1*-deleted strains lacking either *NEW1* alone, or in combination with RQT and/or NGD factors compared to *NEW1* expressing strains (denoted as ‘control’). Cleavage scores are increased upon lack of RQT factors and decreased to a median of close to 0.5 (no effect) upon lack of *CUE2* or *HEL2*, whereas the deletion of *DOM34* has a slightly less pronounced effect. Deletants lacking both, *HEL2* and *SLH1* behave like *HEL2* deletants. Center lines: medians, box limits: 25^th^ and 75^th^ percentiles, whiskers: 1.5× interquartile range from 25^th^ to 75^th^ percentile. All data points are represented by dots.

A cleavage score of 0.5 indicates equal (normalized) numbers of read ends in strains lacking or not lacking New1, ratios >0.5 indicate more read ends in New1-lacking strains, and cleavage scores <0.5 indicate less read ends in New1-lacking strains. To filter out noise, we applied additional thresholds to abundance and relative frequency of read ends (see the ‘Materials and methods’ section for more details). We determined as NGD targets only mRNAs with average (over all replicates) cleavages scores ≥0.75, corresponding to 3× more read ends in New1-lacking strains than New1-containing strains. Although we may thus miss some *bona fide* NGD targets, these thresholds ensure that mRNAs passing them are likely to indeed represent NGD targets. This initial analysis identified 56 NGD candidates (Fig. 4A): 25 from the Ski2-deficient strain and 34 from the Xrn1-deficient strain, with an overlap of only three candidates (*ADH1, GPM1* and *TEF1*; Fig. 4A and Supplementary Table S11). *PGK1* mRNA was only identified in the Ski2- but not the Xrn1-deficient strain. We hypothesize that 3′- and 5′-fragments from the same mRNA might not be (i) stabilized intracellularly and (ii) captured with the same efficiency; e.g. shorter 3′-UTRs would lead to short 3′-fragments, which may be difficult to sequence due to biases against short sequences in our library preparation and in nanopore sequencing. In both strains, the vast majority of NGD candidate mRNAs (∼94% and 96%, respectively; Fig. 4A) belonged to the strongly affected class, supporting the hypothesis that cleavage is caused by C-terminal ribosome collisions.

*The absence of RQT complex factors increases NGD on target mRNA molecules, allowing identification of 139 NGD targets*.

We next sought to determine how NGD varies when genes encoding components of the RQT complex or known NGD factors are deleted in addition to *NEW1* and *SKI2* or *XRN1*. We therefore deleted *SLH1, RQT4, CUE3*, or both, *RQT4* and *CUE3* in *new1∆, ski2∆* and *SLH1, RQT4, DOM34, HEL2, CUE2*, or both *HEL2* and *SLH1* in *new1∆, xrn1∆*. We conducted nanopore sequencing by using these strains as described above (Supplementary Fig. S11). A first observation was that we were able to identify more NGD targets from strains that lacked RQT factors in addition to *NEW1* (Fig. 4B  Supplementary Table S11). Targets identified from RQT-factor lacking strains included most targets identified from strains with intact RQT (Fig. 4C and Supplementary Table S11). In addition to this, the overlap of targets identified from Ski2-lacking and Xrn1-lacking strains was increased from 3/56 (∼5%; Fig. 4A) to 34/171 (∼20%, Fig. 4B). To the contrary, only one NGD target was identified in strains lacking NGD factors Hel2 or Cue2 (*NPL3* in the quadruple deletion of: *NEW1, SLH1, HEL2, XRN1*; Supplementary Table S11). Cleavage scores were increased in Xrn1-lacking strains in the absence of RQT components for NGD targets (Fig. 4D) and for the class of strongly affected mRNAs (Supplementary Fig. S12A). In contrast, they approached 0.5 (equal distribution of read ends in New1-lacking and New1-containing strains) in the absence of factors known to be needed for NGD (endonuclease Cue2, collision sensor Hel2, or splitting factor Dom34; Fig. 4D). In those backgrounds, strongly, mildly and unaffected mRNAs also exhibited cleavage scores at comparable levels (Supplementary Fig. S12A), further supporting that mRNA ends were indeed generated by (canonical) NGD. In contrast, in strains lacking Ski2, cleavage scores remained at similar (high) levels, in the presence and absence of RQT complex members for both, NGD targets (Supplementary Fig. S12B) and strongly affected mRNAs in general (Supplementary Fig. S12C). However, probing *PGK1* mRNA levels by northern blot in strains lacking New1, Ski2 and either Rqt4 or Cue3 (Supplementary Fig. S13), we observed increased formation of cleavage fragments, whereas in strains lacking one or several NGD factors (Hel2, Cue2, Dom34), cleavage was reduced, matching our sequencing-based observations in Xrn1-lacking strains (Fig. 4D). We also note that NGD targets identified from samples lacking RQT factors included mRNAs from the mildly affected and unaffected groups (∼21% and ∼14% in Ski2- and Xrn1-lacking strains, respectively; Fig. 4B). As it is unclear whether those candidates are indeed NGD targets, potentially due to less common collision events, or whether they represent background, in the following analyses, we only focused on the 139 NGD targets from the strongly affected group.

### NGD targets tend to exhibit increased Hel2 recruitment and more frequent and longer ribosome queues/collisions compared to nontarget mRNAs

**Figure 5.**
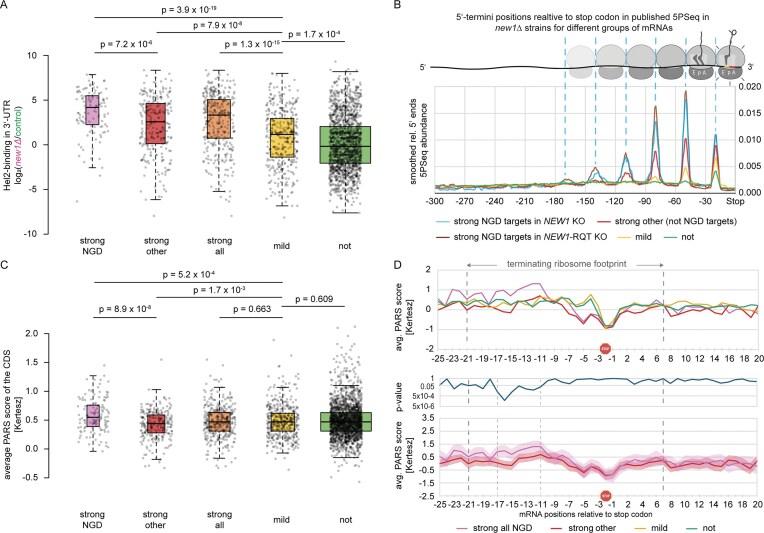
Boxplot analysis of NGD targets detected in the group of strongly affected mRNAs, in comparison to different groups. **(A)** Enrichment of Hel2-binding in the 3′-UTR from CRAC data (20°C), comparing New1-lacking to wildtype yeast **(B)** Comparison of metaplots from 5PSeq [64] re-analysis, comparing NGD targets from the group of strongly affected mRNAs, identified in strains lacking New1 with or without additional lack of RQT factors, other mRNAs from the strongly affected group not identified as NGD targets, mildly and unaffected mRNAs; see Supplementary Fig. S14 for unsmoothed data. **(C)** Boxplot analysis of CDS-wide average PARS scores [88] for groups as in panel (A). **(D)** Position-specific average PARS scores per group, comparing all NGD targets from the strongly affected group of mRNAs to other strongly affected mRNAs not identified as NGD targets, mildly and unaffected mRNAs within the last 25 nt of the coding sequence and the first 20 nt of the 3′-UTR (mean ± 95% confidence interval). See Supplementary Fig. S16A–C for larger sequence window. Boxplot description: Center lines: medians, box limits: 25^th^ and 75^th^ percentiles, whiskers: 1.5× interquartile range from 25^th^ to 75^th^. All data points are represented by dots. Width of the boxes is proportional to the square root of the sample size.

To find whether NGD targets differed from strongly-affected mRNAs not identified as NGD targets, we compared Hel2 crosslinking in their 3′-UTRs. Indeed, 3′-UTR crosslinking was higher for NGD targets compared to other strongly affected mRNAs, which still exhibited higher 3′-UTR crosslinking than mildly and unaffected mRNAs (Fig. 5A). To find whether this might be due to increased collision, we compared 5′-read ends from 5PSeq data within the last 300 nt (see above) between NGD targets identified in presence of RQT or only in the absence of one or several RQT factors, as well as the remaining strongly affected mRNAs not identified as NGD targets at all (Fig. 5B and Supplementary Fig. S14). Here, we found that NGD targets tended to have stronger queuing signals (also seen in queuing scores calculated from 5PSeq data; Supplementary Fig. S15). Targets also form longer queues than non-targets, with even hexasomes distinguishable. However, we did not observe strong differences between targets identified in presence or absence of RQT. This suggests that for those targets, collisions are already strong and trigger NGD in presence of RQT. The vast majority of NGD targets, including those only identified in absence of RQT components, already exhibit cleavage scores >0.5 even in their presence (Fig. 4D). This indicates that the absence of RQT components increases NGD on the most queuing-prone mRNAs, so targets become identifiable, rather than causing NGD on mRNAs not targeted by NGD in presence of RQT activity. In all, this suggests that, in New1-lacking yeast, ribosome collisions need to pass a certain threshold for mRNAs to be targeted by NGD. Previously, it has been suggested that ≥3 collided ribosomes would be necessary to trigger NGD [7]. In line with this, and to our surprise, even in the absence of RQT-catalysed ribosome splitting as a potential alternative rescue mechanism, many mRNAs were still not identified as NGD targets, despite the presence of (somewhat weaker) collisions on that subset of mRNAs. This suggests that other rescue mechanisms exist for those mRNAs. One such mechanism could be translation termination itself, which may succeed eventually, even after ribosome collisions have happened, possibly despite Hel2-dependent ribosome ubiquitination. An alternative could be deubiquitination of Hel2-ubiquitinated ribosomes, as recently described [104], which may allow for efficient termination.

### NGD targets tend to be more strongly folded upstream of the stop codon

Having found that NGD only targets a subgroup of mRNAs, and that this subgroup is defined by more frequent and longer collisions, we sought to find what makes this subgroup different from collision-prone strongly affected mRNAs that are not NGD targets. Here, we compared a number of mRNA features, including, among others, mRNA folding propensity, previously quantified for the yeast transcriptome *in vitro* (resulting in ‘PARS scores’ at nucleotide resolution of each yeast mRNA [88]). Here, we found that NGD targets tended to exhibit higher folding propensities in the coding sequence (Fig. 5C). Focusing on the region around the stop codon (Fig. 5D and Supplementary Fig. S16A–C), we then found significant position-specific differences in PARS scores upstream of stop codons, at positions −17 to −11, relative to the 3′-UTR (Fig. 5D). This region is within the ribosomal footprint of the terminating/stalled ribosome. We hypothesize that this stronger folding propensity might cause additional strain on translation, even before ribosomes reach the problematic C-terminal codon, potentially increasing the risk of ribosomal collisions. Alternatively, this folding propensity could facilitate Cue2-cleavage, in a manner that would yet have to be determined. Finally, it is also possible that an increased folding propensity makes alternative rescue mechanisms, e.g. termination itself, or ribosome splitting by other mechanisms, more difficult to proceed, preventing rescue of collisions, and hence favoring NGD. Interestingly, although folding propensity significantly differed in this region, we were unable to determine any nucleotide or amino acid sequence pattern, apart from a slight underrepresentation of the codon Lys(AAA) at the C-terminal position in NGD targets (Supplementary Fig. S16D–G), suggesting that structure, not sequence is the determining feature here.

#### mRNA cleavage during NGD occurs at multiple positions across NGD targets, is altered upon RQT dysfunction and abolished upon lack of NGD factors

**Figure 6.**
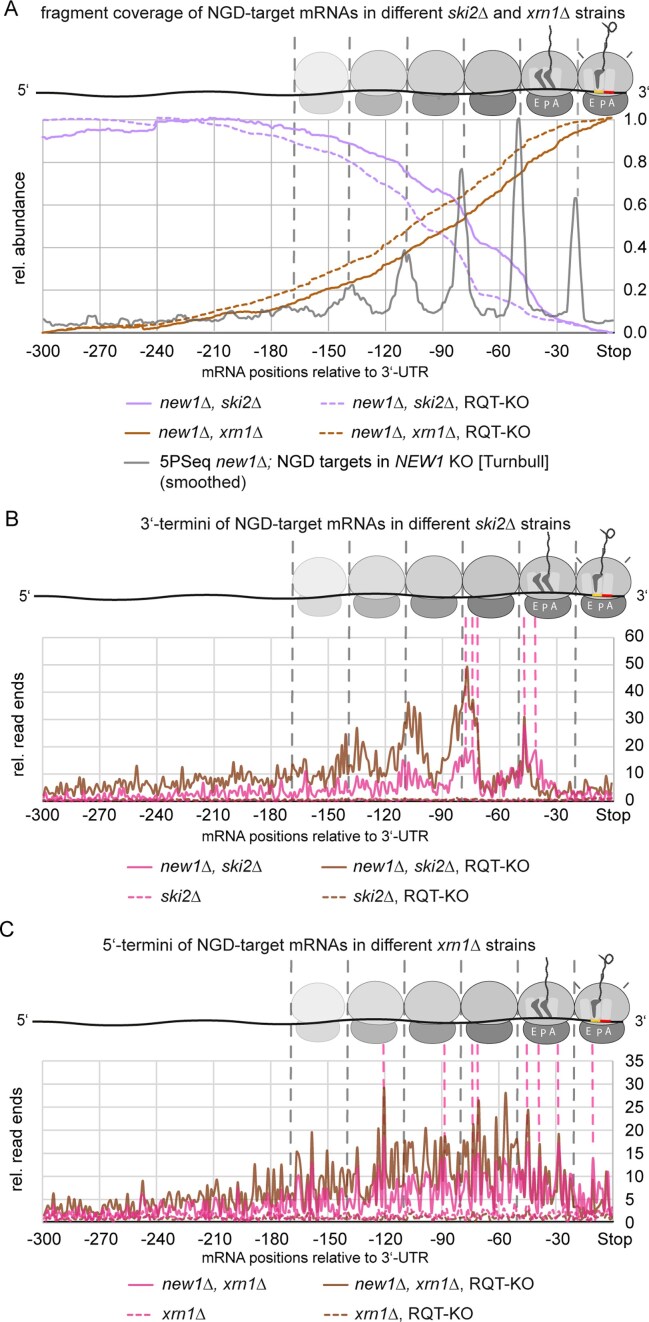
Analysis of endonucleolytic fragments by nanopore sequencing. **(A)** Relative coverage of nanopore sequencing data (from fragments only) across the last 300 nt of the CDS (including stop codon) for mRNAs targeted by NGD in the respective strains lacking *NEW1* and either *SKI2* or *XRN1* without additional loss of RQT factors. Data are shown for strains lacking *NEW1* and either *SKI2* or *XRN1*, and for strains additionally lacking RQT factors. The class of RQT-knockouts (RQT-KO) includes deletions of *SLH1, RQT4* (for *XRN1*-lacking and *SKI2*-lacking strains) and additionally deletions of *CUE3* and *RQT4 *+ *CUE3* (only for *SKI2*-lacking strains). Additionally, the relative, smoothed abundance of 5′-termini from the re-analysis of 5PSeq data [64] in the absence of *NEW1* is shown for NGD targets detected in *NEW1* deletants, with indicated ribosome positions (gray dashed lines). **(B)** Relative distribution of nanopore 3′-read ends across the last 300 nt upstream of the 3′-UTR for NGD targeted genes, in strains lacking *SKI2*. Dashed pink lines indicate cleavage sites enriched without RQT-KO. **(C)** Relative distribution of nanopore 5′-read ends for strains lacking *XRN1*. Additional, related data shown in Supplementary Figs S17–S20. Strains lacking *SKI2 n* = 2 per strain, strains lacking *XRN1 n* = 3 per strain, except *new1Δ, xrn1Δ n* = 2. Data from several strains combined for the RQT-KO group.

Having identified multiple NGD targets, we now examined whether they all are processed similarly to our example mRNA, *ADH1*, representing the first direct observation of NGD processing fragments of endogenous target mRNAs on a larger scale. Contrary to the common stalling scenario, e.g. on poly(A) tracts, where stalling can occur over a range of positions, in our case, the stall position is clearly defined, with the stop codon positioned in the A-site of the stalled ribosome [63, 64]. In turn, this allows very precise localization of cleavage sites with respect to the positions of stalled and collided ribosomes. To analyze cleavage, for Ski2- as well as Xrn1-lacking strains, we initially focused on the group of targets identified in New1-lacking strains (without further deletion of RQT factors). We first compared sequencing coverage of cleavage fragments over the last 300 nt in front of the 3′-UTR, including the stop codon (positions −1 to −3) (Fig. 6A, see the ‘Materials and methods’ section for details). Here, we found that 5′-fragments in the *SKI2*-deleted and 3′-fragments in the *XRN1*-deleted strains roughly complemented each other. In case of Ski2-lacking strains, they were shifted by about one ribosome footprint (∼30 nt), for Xrn1-lacking strains, the shift was smaller. To gain deeper insights into the actual cleavage pattern, we now analysed read ends across the target mRNAs (Fig. 6B and C). Metaplots were generally very similar for strains lacking different RQT components (but containing Hel2) (Supplementary Fig. S17A–D), allowing to average over all replicates of RQT-deleted variants, among the Ski2- (Fig. 6B and Supplementary Fig. S18A and B) and among the Xrn1-lacking strains (Fig. 6C and Supplementary Fig. S18C and D), revealing increased cleavage upon loss of RQT-factors. For Ski2-lacking strains, we observed a strong ∼30 nt periodicity of cleavage signals, both in presence and absence of functional RQT complex (Supplementary Fig. S20A and B).

In the presence of intact RQT, the majority of cleavages occurred close to the E-site, as well as closer to the 5′-end of the ribosome footprint. In contrast, in the absence of RQT components, maximal cleavage occurred only near the 5′-end of the footprint. This is consistent with the previous suggestion [47] that full RQT-based ribosome splitting activity is required for Cue2-mediated cleavage to occur within ribosomes, whereas cleavages upstream of collided ribosomes can occur without RQT activity. Signals generated from Xrn1-lacking strains were more disperse (Fig. 6C) and did not exhibit any clear periodicity (Supplementary Fig. S20C and D). Previously, variable lengths of 3′-fragments have been observed in Xrn1-lacking yeast strains, which were attributed to the activity of the 5′-to-3′-exonuclease Dxo1 [7]. It is thus likely that Cue2 dependent 3′-fragments can be trimmed to variable, shorter lengths via Dxo1 activity. Despite differences in periodicity, *SKI2*- and *XRN1*-deleted strains exhibited a similar distribution of cleavage signals to the different ribosomes’ footprints, with maximal cleavage being reached within the footprints of the first and second collided ribosomes (in presence of all RQT factors) or the second to fourth collided ribosomes (in the absence of RQT factors; Fig. 6B and C). The near-complete absence of cleavage in the stalled or first collided ribosome in the absence of RQT factors further supports the hypothesis that NGD in a context in which RQT cannot act (in that case due to a self-cleaving reporter in which ribosomes stall at the very 3′-end of the mRNA, prohibiting Slh1 binding [7]) requires at least a collided trisome [7].

Comparing now NGD targets identified in presence of intact RQT to those only identified upon deletion of RQT factors (Supplementary Fig. S18C and D), we observed that the increase in cleavage signal upon deletion of RQT factors was exacerbated for targets only identified in RQT deletion strains. In comparison, the top abundant non-targets among ‘strongly affected’ mRNAs revealed much lower cleavage signals and only minor differences upon deletion of RQT factors (Supplementary Fig. S18B and D). In strains lacking Xrn1, signals for this group did not exceed background seen in New1-containing controls. However, in the case of Ski2-lacking strains, patterns resembled those found in NGD targets, though at much lower amplitude. This might imply that NGD happens, sporadically, on those sequences, but at much lower frequency.

Focusing on strains lacking known NGD factors (Supplementary Fig. S19A–F), we observed strongly reduced cleavage signals (Supplementary Fig. S19A and B). Strains lacking Hel2, Cue2, or a combination of Hel2 and Slh1 did not exhibit cleavage beyond background in New1-containing controls (Supplementary Fig. S19C–F), further validating that target mRNAs are subject to canonical NGD, requiring Hel2 and endonucleolytic cleavage by Cue2.

Interestingly, in Dom34-lacking strains, minor cleavage signals beyond control were observable, suggesting that NGD activity is potently reduced, but not fully inhibited upon lack of Dom34 (Supplementary Fig. S19C).

All changes observed on the metagene level were also observable when analyzing the *ADH1* mRNA alone (Supplementary Figs S21–S24), although we note that here, cleavage within the first collided ribosomes was already lower, compared to other mRNAs, even in the presence of all RQT components. As the *ADH1* mRNA appears to be particularly prone to queuing and NGD (Fig. 3B), it is possible that other cleavage sites are preferred, due to long queues. On the other hand, for *ADH1*, the increase in cleavage score upon loss of RQT components was extremely modest in the *XRN1* deletion background, suggesting that RQT may not have been very active on ribosomes stalled on the *ADH1* mRNA in the first place, possibly due to features specific to this sequence. As one example, according to PARS score [88] analysis, *ADH1* features among the most folded mRNAs. This may also explain the particularly strong NGD signature, with *ADH1* mRNA exhibiting one of the highest cleavage scores of the whole dataset, as well as the low occurrence of cleavage within the first collided ribosomes, which we had interpreted as RQC-coupled NGD. Compared to average, we also still observe considerable cleavage upon loss of Dom34. This shows that Dom34 is likely not essential for NGD on at least some targets but does promote NGD activity.

#### NGD targets are downregulated at the mRNA and protein levels

**Figure 7.**
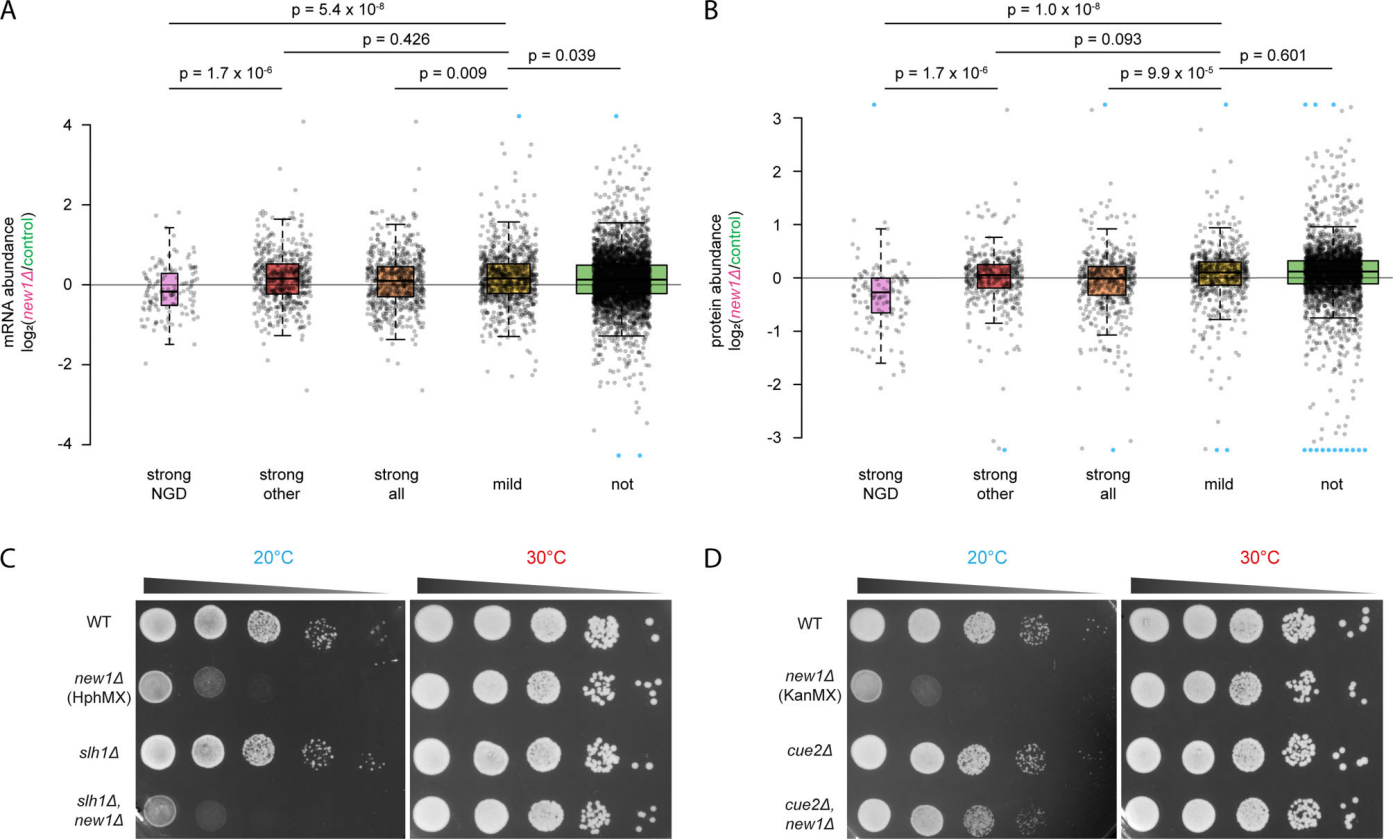
Consequences of NGD for yeast cells lacking New1. Boxplot analysis of fold-changes in mRNA, measured by nanopore sequencing **(A)** and protein abundance, measured by proteomics **(B)** between *new1Δ* and wildtype, both grown at 20°C. NGD targets from the strongly affected group are compared to non-targets (strong other) from the strongly affected group of mRNAs, and to all strongly, mildly, and unaffected ones. Boxplot description: Center lines: medians, box limits: 25^th^ and 75^th^ percentiles, whiskers: 1.5× interquartile range from 25^th^ to 75^th^. All data points are represented by dots. Extreme outliers are represented below minimum or above maximum in blue. (**C, D**) Cold sensitivity of different mutants analysed by spot assay: Serial dilutions were prepared starting from an OD_600_ of 1 (four consecutive 1:10 dilutions) and 10 µl per dilution spotted on YPD plates. Plates were incubated at either 20°C or 30°C (control), as indicated and imaged after 3 days. **(C)** Lack of RQT factor Slh1 aggravates cold sensitivity of New1-lacking yeast. **(D)** Lack of NGD endonuclease Cue2 rescues cold sensitivity of New1-lacking yeast.

To assess changes in mRNA abundance upon deletion of *NEW1*, we performed nanopore sequencing employing the standard library preparation protocol without *in vitro* polyadenylation on RNA samples from *new1Δ* strains (containing the *SKI2* and *XRN1* gene), and compared RNA levels to wildtype. Here, levels of mRNAs that we identified as NGD targets were (on average) downregulated upon lack of New1. To the contrary, levels of other mRNAs from the strongly affected class that were not identified as NGD targets, mildly affected or unaffected mRNAs were not (Fig. 7A). Some of the most downregulated mRNAs are *ADH1, PGK1*, and *GPM1*. Protein levels showed a very similar, though somewhat stronger trend (Fig. 7B). The fact that fold-changes in the levels of non-NGD targets from the strongly affected class of mRNAs did not differ significantly from changes in the mildly affected group, on both, mRNA and protein level suggests that the strongly affected, non-NGD target mRNAs are effectively rescued, and that our approach has indeed identified the majority of NGD targets. The fact that the regulation appears weak could be due to additional perturbations, especially at the mRNA level, where specific genes could be transcriptionally upregulated, e.g. to compensate for NGD-based downregulation. This appears especially probable since many important genes from metabolism and from the translation machinery are targets of NGD, thus causing major perturbations for the cells. As mentioned, downregulation of NGD targets appears stronger at the protein than at the than at the mRNA level. One possible reason for this is the fact that collision frequency would be highest on the most translated mRNAs. Thus, the mRNA molecules that should produce the highest protein output would be most prone to degradation, while less translated individual molecules would be less likely to be degraded, thereby affecting protein output more strongly than template abundance. In addition to this, besides NGD, another possible outcome of ribosome collisions is the degradation of nascent peptides via RQC. This can happen independently from NGD, but would require prior ribosome splitting, e.g. by the RQT complex. Although beyond the scope of this work, it is possible that proteins are downregulated not only as a consequence of mRNA degradation due to NGD, but also by RQC.

### NGD is a major determinant of cold sensitivity in New1-lacking yeast

Having identified 139 NGD targets, featuring prominent metabolic genes, and considering positive genetic interactions between the *NEW1* gene and several genes encoding NGD factors [50], we asked whether NGD is the cause for cold sensitivity [63–65] reported for New1-lacking yeast. To this end, we performed spot assays to compare the viability and growth of wildtype yeast, single deletants lacking New1, Slh1 or Cue2, and of double deletants lacking New1 in combination with Slh1 or Cue2 (Fig. 7C and D). While no growth differences were observable at 30°C, at 20°C lack of New1 alone caused a severe delay in growth, as previously reported [63–65], whereas lack of Slh1 in addition to New1 even slightly aggravated this phenotype (Fig. 7C). Contrary to this, lack of Cue2 in addition to lack of New1 partially rescued growth, almost to wildtype levels (Fig. 7D), indicating that NGD is a major determinant of cold sensitivity in New1-lacking yeast. Whether remaining growth differences are due to minor NGD pathways still active in the absence of Cue2, or rather due to other effects is unclear. We conclude that, for yeast cells, the presence of New1 represents a selective benefit, as the factor prevents mRNA degradation by NGD for an important subset of mRNAs.

### UV crosslinking and analysis of cDNA reveals New1 interactions with transfer, ribosomal, and messenger RNA

Finally, to address how New1 might protect mRNA from C-terminal ribosome collisions, we performed CRAC using strains in which New1 was (HTP) tagged, and in which collision sensor Hel2 was either present or absent. We compared these data to complementary CRAC data for Hel2 in presence and absence of New1. Here, we found that New1 binds mRNA, rRNA, and tRNA, consistent with a role in translation (elongation and/or termination; Supplementary Table S12). However, we did not see major differences depending on presence or absence of Hel2. A detailed description is given in Supplementary Text S2, Supplementary Figs S25 and S26. tRNA binding was especially high for tR(CCU), the tRNA that decodes the queue-inducing codon R(AGG), but not other tRNAs decoding other queue-inducing codons (Supplementary Table S13). Several of New1’s major crosslink sites on 18S rRNA (Supplementary Fig. S25A and B) coincided with crosslink sites of Hel2, suggesting both factors’ binding may be mutually exclusive, and potentially explaining changes in Hel2 crosslinking upon loss of New1 (Supplementary Fig. S25A). Moreover, various major New1-rRNA crosslink sites also closely matched published cryo-electron microscopy (cryo-EM) structures (Supplementary Fig. S25B). Nevertheless, additional ones may be explained by either flexible regions of New1 not resolved in the cryo-EM structure, or by potential additional, transient interactions. New1’s mRNA binding (Supplementary Fig. S26) revealed a preference for binding to the 3′-UTR, similar to Hel2-binding. Interestingly though, we did not observe any binding preference for mRNAs containing queue-inducing C-terminal codons. In conclusion, our crosslinking data show rRNA interactions of New1 which are in line with published cryo-EM data [63, 64], as well as additional interactions with rRNA, mRNA and tRNA which have not been described before. These results suggest that New1 is not exclusively engaging terminating ribosomes on queuing-prone mRNA C-termini, but ribosomes at all positions of all mRNAs. This would be in line with structural data showing New1 bound to ribosomes in different translation states [64].

## Discussion

**Figure 8.**
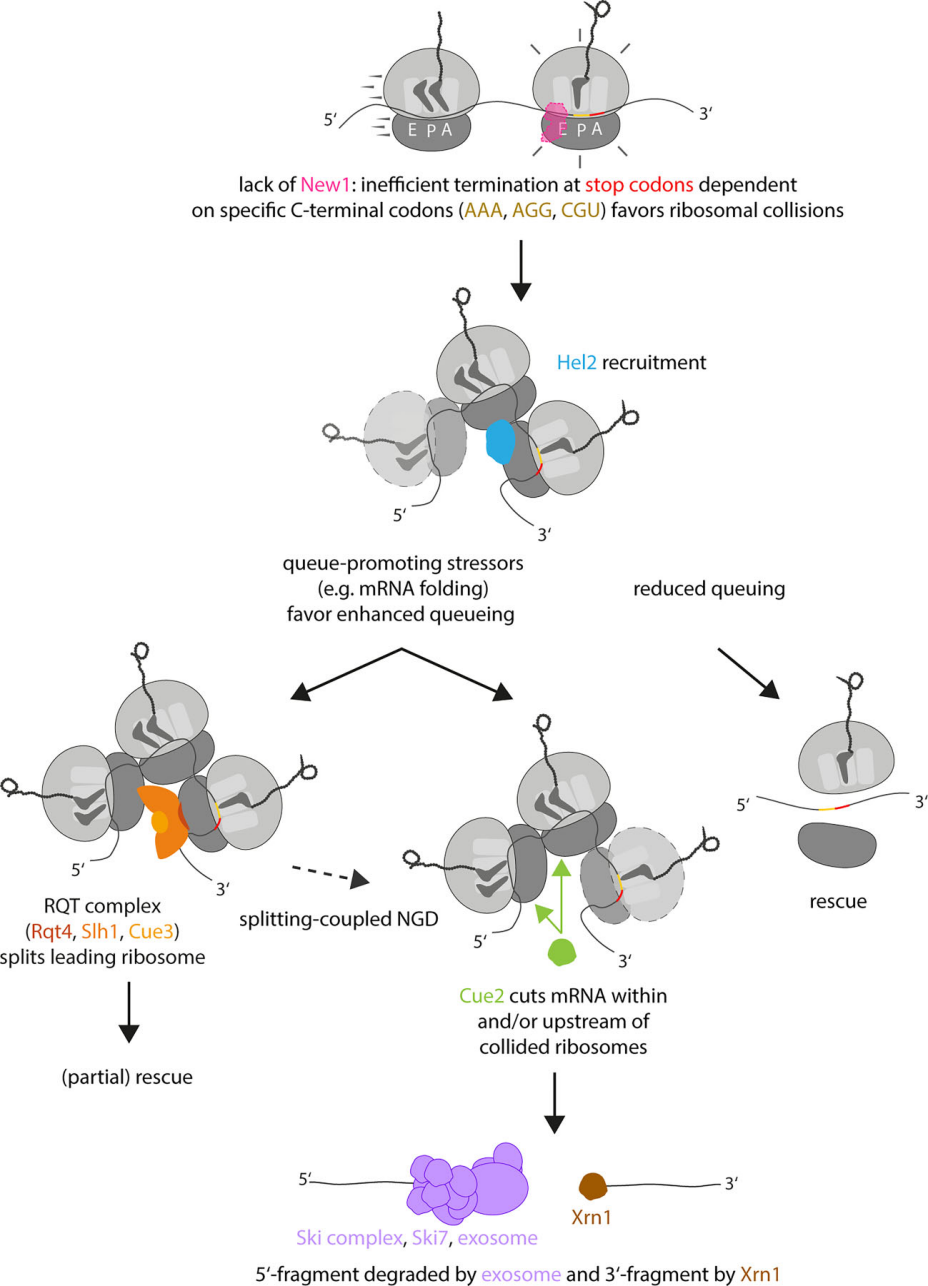
Proposed order of events in the absence of New1. The dashed arrow represent RQC-coupled NGD, where Cue2 cleaves within ribosomes, requiring RQT-catalyzed splitting. The translucent ribosome would only be present under queue-promoting conditions (second row) or in the RQC-uncoupled pathway (third row, middle).

We propose the following order of events related to translation, translation termination and surveillance upon New1 deficiency (main steps summarized in Fig. 8): Lack of New1 leads to inefficient termination at stop codons, dependent on specific C-terminal codons (most prominently: K(AAA), R(AGG), R(CGU)), and favors ribosomal collisions. On all or most of the mRNAs, collisions are sensed by Hel2, which ubiquitinates uS10 of affected ribosomes. For ∼140 of the >600 strongly affected mRNAs, NGD occurs, with Cue2-mediated endonucleolytic cleavage occurring in part within or upstream of the collided or following ribosomes (in presence of functional RQT). Split 60S:peptide complexes are possibly targeted to RQC (not further investigated in our study). Alternatively, Cue2 can also cleave mRNA upstream of collided ribosomes, without prior ribosome splitting via the RQC-uncoupled NGD pathway. Indeed, we show that upon lack of New1, NGD becomes more prominent on NGD targets in the absence of functional RQT complex. This shows that while the RQT complex enables RQC-coupled NGD, it is also able to rescue some mRNA molecules that would otherwise be targeted to Cue2-mediated NGD by resolving queues, as previously described [28]. Cleavage fragments are then degraded exonucleolytically by the exosome with the help of the Ski complex, and by Xrn1 (as is consensus in the literature). The degradation of NGD targets (complete list of targets identified in Supplementary Table S11) further propagates to the reductions in levels of corresponding proteins, including several essential metabolic enzymes (e.g. Pgk1 and Gpm1) and translation components, which are among the most abundant yeast proteins (e.g. eEF-1 alpha and eEF-1 beta). The remaining mRNAs from the strongly affected class were not identified as NGD targets. Although in some cases this could be due to lack of sensitivity of our method, changes in the levels of mRNAs and of proteins translated from those did not differ significantly from changes in the mildly or unaffected classes of mRNAs. This suggests that they are indeed not targeted to NGD, or if so, only to a low degree. In turn, this implies that despite recruitment of Hel2, those mRNAs are effectively rescued, potentially by deubiquitination via Ubp2 and/or Ubp3, as recently described [104, 105]. Another possibility is that ribosomes eventually undergo termination, thereby relieving the ribosome collision. It is thus far unclear whether termination of the leading ribosome is still possible if stalling and collision happen at the stop codon, or whether ribosomes would then be trapped in the collided state, especially upon Hel2-mediated ubiquitination.

One factor that differed between mRNAs that were identified as NGD targets and those that were not was the degree of folding in the coding sequence, where we detected increased propensity for being structured few codons upstream of the stop codon. If structure is a determinant, this can conceivably explain the difference in growth phenotype observed between 20°C and 30°C (Fig. 7C and D) although neither queuing [63, 64], nor Hel2 recruitment (Fig. 1A–C and Supplementary Figs S2, S3, and S5) varied considerably between the temperatures. Structure formation would be expected to vary in a temperature-dependent manner and could impede alternative rescue mechanisms in the absence of New1.

Concerning the effect of lack of New1, the average decrease in protein levels observed for NGD-targets in the strongly affected group of mRNAs was greater than that of corresponding mRNA levels (Fig. 7A and B). Two possible explanations we suggest are that either mRNA templates that are more strongly translated would be more prone to collision and would therefore be more strongly depleted from the pool than other, less translated molecules, thereby affecting protein output more strongly than template abundance. Alternatively, or in addition, it is possible that the decrease in protein levels is more strongly influenced by the RQC pathway, which can occur in parallel to or independently of NGD. Although we did not investigate the effect of New1 on RQC in this work, we note that the *NEW1* gene exhibits positive genetic interactions with the *RKR1/LTN1* gene [50], which encodes the central RQC factor Ltn1, responsible for ubiquitination of nascent peptides on stalled ribosomes [106]. RQC-based degradation of peptides from collided ribosomes may thus also be triggered in the absence of New1.

Interestingly, we observe an over-representation of strongly affected lysine and arginine codons at C-termini, compared to their frequency in gene bodies in *S. cerevisiae*, whereas alternative, unaffected codons encoding the same amino acid are under-represented at C-termini (Table 1). This is despite the fact that several important cellular proteins are, as a consequence, strongly reduced upon lack of New1. This striking over-representation may suggest a potential fitness benefit by retaining, or even favoring queue-inducing C-terminal codons over iso-coding non-queue-inducing C-terminal codons [e.g. favoring K(AAA) over K(AAG)], when New1 is available. The over-representation of queuing-prone C-terminal codons, which requires New1 to avoid fitness losses, also suggests that New1 could have regulatory potential. As an example, New1 expression was found to be strongly decreased in stationary phase [107], where this may contribute to downregulation of New1’s target genes. As another hypothesis, the highly affected C-terminal codons may allow longer time for termination, e.g. to allow proper folding or binding of co-factors/interactors, before peptide release, while still maintaining proper termination in the presence of New1.

**Table 1.** C-terminally stalling codons are overrepresented at the C-terminus in *S. cerevisiae* (from our own reference files, see the ‘Materials and methods’ section)

	Codon	% CDS (total count)	% C-terminus (total count)	Ratio % C-terminal/% CDS (total C-terminal/total CDS)
K	**AAA** (strong)	59 (126 170)	76 (552)	1.29 (0.44%)
(AAR)	**AAG** (not)	41 (89 468)	24 (172)	0.59 (0.19%)
	**AGG** (strong)	21 (27 768)	24 (69)	1.14 (0.25%)
R	**AGA** (not)	47 (61 905)	33 (95)	0.70 (0.15%)
(AGR/CGN)	**CGU** (strong)	14 (18 452)	24 (71)	1.71 (0.38%)
	**CG(A/C/G)** (mild)	17 (22 342)	19 (55)	1.12 (0.25%)

Percentage of specific lysine and arginine codons of total lysine or arginine codons at C-termini and in CDS are given, as well as ratio of percentage at C-terminus and CDS.

Although we are beginning to better understand the consequences of lack of New1, what exactly the protein does is still unclear. Cryo-EM studies [63, 64] suggest that its binding mode is similar to that of its better characterized homologue eEF3. Our crosslinking data also support that New1 interacts with ribosomes *in vivo*, and that it does so along the whole mRNA; however, with a preference for the 3′-terminus, suggesting a preference for terminating ribosomes. Although the absence of New1 only induces ribosome collisions and related downstream consequences on specific mRNAs, New1 itself is apparently able to interact with terminating ribosomes on any mRNA.

Why does lack of New1 not affect all mRNAs and terminating ribosomes equally, in terms of causing collisions? We suggest that another, additional factor, e.g. eEF3, which has also been implicated in translation termination [60], is able to fulfil the same role as New1 on most, but not all mRNAs/tRNAs. This would be in line with published findings that New1 overexpression was able to compensate to some degree for eEF3 deficiency, but presence of eEF3 was not able to compensate for *NEW1* deletion [63]. Therefore, specifically in the context of translation termination, New1 seems to be required only for a subset of mRNAs/tRNAs. On the structural level, this differential requirement could be caused by the chromodomain, which is shorter for New1 than for eEF3 [63]. At least in the case of eEF3, this domain interacts with the ribosome’s L1 stalk [55]. Alternatively, New1 could be probing all terminating ribosomes, but only act on a subset of them that exhibits problems in termination. Such problems could be caused, e.g. by the local structure of mRNA, tRNA and/or termination factors within the ribosome. Indeed, it is highly likely that the tRNAs that decode affected codons are major determinants of the problems in termination and the queuing phenotype. tRNA specificity probably explains the codon dependence we and others observe [64]. It has been convincingly demonstrated that deletion of the main tRNA decoding the R(AGG) codon [*HSX1*/tR(CCU)] inhibited C-terminal ribosome queuing upon deletion of *NEW1* [64] on the subset of mRNAs ending in R(AGG), followed by weak stop codon ‘UGA’ and the nucleotide cytosine. In addition, specific tRNAs have recently been shown to recruit the CCR4-NOT complex to human translating ribosomes, triggering mRNA decay, whereas structural properties of other tRNAs prevent this recruitment [108]. In a similar fashion, tRNA structure could hamper proper recruitment of termination factors to the terminating ribosome, as suggested by Turnbull *et al.* [64]. As an additional feature, tRNA modification could be a determinant of termination problems [64]. However, arginine codons R(CGA), R(CGC), and R(CGU) are all decoded by the same tRNA but are not affected by lack of New1 to the same extent according to our data (Fig. 1B: R(CGA), R(CGC) – mildly affected; R(CGU) – strongly affected). This suggests that it is not the tRNA alone that determines the extent of problems in termination. A role for mRNA structure has previously been demonstrated for difficult-to-decode codon stretches, where the conformation of mRNA within the ribosome interferes with tRNA binding [8, 9]. Finally, we could also envision that the local structure of the ribosome itself could play a role, where, e.g. the ribosomal L1 stalk might be in different conformations, depending on the P/E-site or E-site tRNA.

New1 is not the only ABCF protein involved in resolving problematic translation events. Multiple bacterial ABCF proteins have been described to resolve translational stalls caused by antibiotics [54, 109–111] or difficult-to-decode codon stretches, including both, polyproline and charged amino acid stretches [112–115]. The bacterial ABCF proteins share the function of relieving translational difficulties with elongation factor EF-P. Interestingly, EF-P's yeast ortholog, eIF5A, has been implicated with resolving similar difficulties, in addition to resolving or preventing termination deficits and ribosomal queueing at stop codons [101]. This further demonstrates functional similarities in the resolution of yeast and bacterial translation difficulties.

Like New1 [63, 64], those ABCF proteins interact with the E-site; however, they contain a P-site tRNA-interacting domain not present in New1, which is important for their activity [111]. How New1 exerts an activity that depends on the P-site tRNA, without this domain remains to be elucidated. It is possible that the N- and C-terminal extensions of the New1 structure that have not been resolved yet (Supplementary Fig. S25B) are required for this. Although ribosome collision at the stop codon is an important phenotype in *new1Δ*, the structure of the collided ribosomes from such strains has not been determined yet. Since these collisions initiate with ribosomes containing the stop codon in the A-site, it remains to be seen whether these ribosomes already contain termination factors, or whether the A-site remains vacant, e.g. due to structure of the P-site tRNA. At least in presence of New1, ribosomes containing both, New1 and eRF1 have been observed [64]. Such variations may differentiate C-terminal collided ribosomes from ribosomes collided on other stall-inducing sequences, and may also have consequences on downstream surveillance pathways.

Beyond giving insights into the consequences of New1 dysfunction, our work exemplifies, how (in the absence of New1) highly abundant proteins are mRNA-specifically downregulated by the NGD pathway, under physiological conditions, in the absence of translation inhibitors or mRNA damage. NGD upon lack of New1 and NGD on other endogenous substrates under different conditions might differ to some degree. However, the clearly determined position of the stalled ribosome with the stop codon in the A-site represents a highly defined system, which has allowed for unprecedented insights into NGD endonucleolytic cleavage across a whole variety of targets, in presence and absence of RQT function. Having been able to confirm observations previously made on exogenously expressed reporters, now on a whole set of endogenous targets, we firmly believe that our findings will also be applicable to other scenarios in which NGD occurs.

Collectively, our work demonstrates that New1 represents a protective factor that is needed in yeast to avoid NGD. Presence of New1, on the other hand, enables budding yeast to be enriched for specific lysine and arginine codons in the stop codon context, which appear to confer selective advantages as long as potentially deleterious consequences, including NGD, are avoided.

## Supplementary Material

gkag047_Supplemental_File

## Data Availability

CRAC data are available at GEO, under the accession number GSE275093. Nanopore sequencing data are available at Bioproject (Project Number: PRJNA1291453). The documentation of the computational pipeline used for nanopore sequencing analysis, including the source code is available in Zenodo [86]. The mass spectrometry proteomics data have been deposited to the ProteomeXchange Consortium via the PRIDE [116] partner repository with the dataset identifier PXD055983.
