# Supplementary material for: Yeast elongation factor homolog New1 protects a subset of mRNAs from degradation by no-go decay
Source: Nucleic Acids Res. 2026 Jan 29;54(3):gkag047. doi: 10.1093/nar/gkag047 (PMC12852954; doi:10.1093/nar/gkag047)
Supplement: gkag047_Supplemental_File [file gkag047_supplemental_file.pdf]

Supplementary Information for

Yeast elongation factor homolog New1 protects a subset of mRNAs from degradation by no-go decay

Max Müller[1], Lena Sophie Tittel[1], Elisabeth Petfalski[2], Kaushik Viswanathan Iyer[1], Alina-Andrea Kraft[1], Stefan Pastore[1], Tamer Butto[1], Marie-Luise Winz[1]

[1] Institute of Pharmaceutical and Biomedical Sciences, Johannes Gutenberg-University Mainz, Mainz, 55128, Germany
[2] Centre for Cell Biology, University of Edinburgh, Edinburgh, EH9 1BF, United Kingdom

* To whom correspondence should be addressed. Tel: +49-6131-3922482;
Email: mwinz@uni-mainz.de

Content:

Supplementary Figure S1 **p. 3**

Supplementary Figure S2 **p. 4**

Supplementary Figure S3 **p. 5**

Supplementary Figure S4 **p. 6**

Supplementary Figure S5 **p. 7**

Supplementary Figure S6 **p. 8**

Supplementary Figure S7 **p. 9**

Supplementary Figure S8 **p. 10**

Supplementary Figure S9 **p. 11**

Supplementary Figure S10 **p. 12**

Supplementary Figure S11 **p. 13**

Supplementary Figure S12 **p. 14**

Supplementary Figure S13 **p. 15**

Supplementary Figure S14 **p. 16**

Supplementary Figure S15 **p. 17**

Supplementary Figure S16 **p. 18**

Supplementary Figure S17 **p. 19**

Supplementary Figure S18 **p. 20**

Supplementary Figure S19 **p. 21**

**Supplementary Figure S20**	**p. 22**
**Supplementary Figure S21**	**p. 23**
**Supplementary Figure S22**	**p. 24**
**Supplementary Figure S23**	**p. 25**
**Supplementary Figure S24**	**p. 26**
**Supplementary Figure S25**	**p. 27**
**Supplementary Figure S26**	**p. 28**
**Supplementary Table S1**	**p. 29**
**Supplementary Table S2**	**p. 35**
**Supplementary Table S3**	**p. 38**
**Supplementary Table S4**	**p. 39**
**Supplementary Table S5**	**p. 40**
**Supplementary Table S6**	**p. 41**
**Supplementary Table S7**	**p. 42**
**Supplementary Table S8**	**p. 43**
**Supplementary Table S9**	**p. 44**
**Supplementary Table S10**	**p. 47**
**Supplementary Table S11**	**p. 49**
**Supplementary Table S12**	**p. 52**
**Supplementary Table S13**	**p. 53**
**Supplementary Methods**	**p. 54**
**Supplementary Text S1**	**p. 59**
**Supplementary Text S2**	**p. 62**
**Supplementary References**	**p. 66**

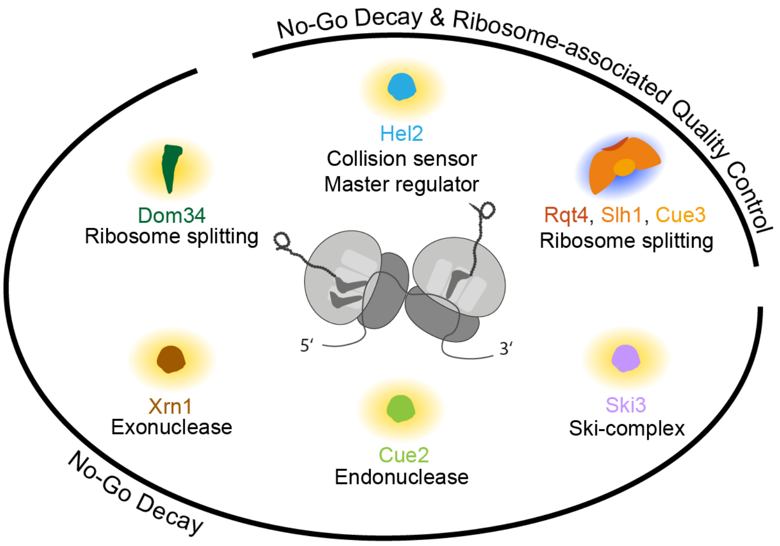

Supplementary Figure S1: No-go decay (and ribosome-associated quality control) factors encoded by genes that exhibit positive (yellow halo) or negative (blue halo) genetic interactions with *NEW1*, and their roles in no-go decay.

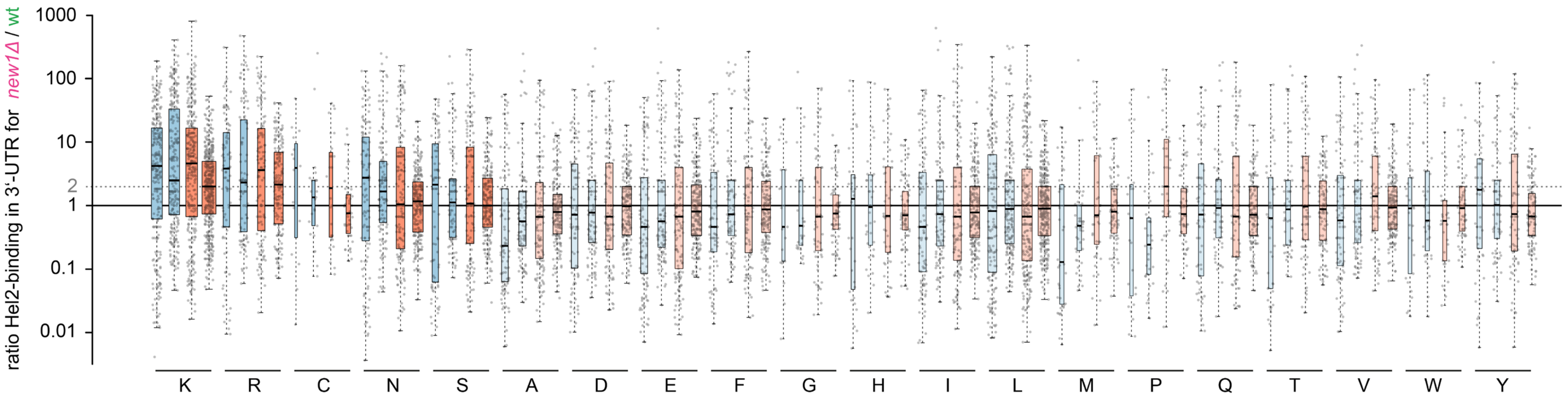

Supplementary Figure S2. Ratio of Hel2 binding in 3′-UTR for *new1Δ*/wildtype at 20°C (blue, 2 replicates), and 30°C (red, 2 replicates) for mRNAs, plotted by C-terminally encoded amino acids. Non-affected amino acids are shown with higher transparency. Center lines show the medians, box limits indicate the 25th and 75th percentiles, as determined by R software; whiskers extend 1.5 times the interquartile range from the 25th and 75th percentiles. All data points are represented by dots. Width of the boxes is proportional to the square root of the sample size. Related to **Figure 1**.

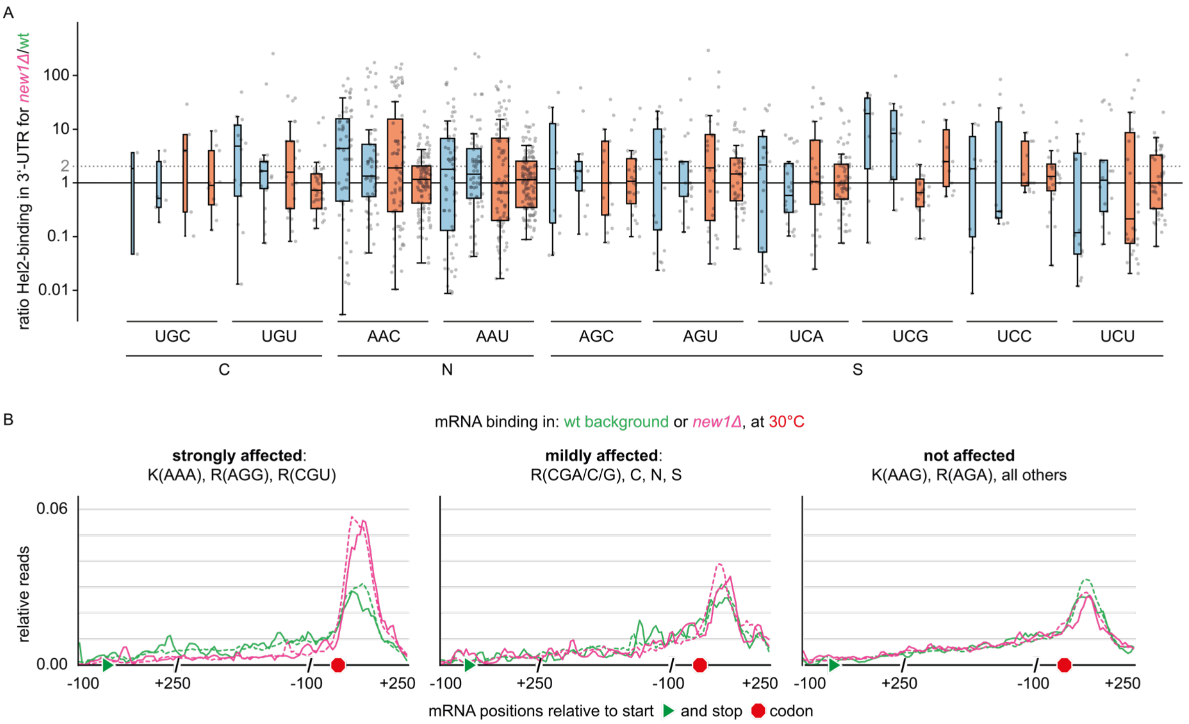

Supplementary Figure S3. Related to **Figure 1.** (**A**) Ratio of Hel2 binding in 3'-UTR for *new1Δ*/wildtype for all cysteine, asparagine, and serine codons at 20°C (blue, 2 replicates), and 30°C (red, 2 replicates). Boxplot description: Center lines: medians, box limits: 25th and 75th percentiles, whiskers: 1.5x interquartile range from 25th - 75th percentile. All data points are represented by dots. Width of the boxes is proportional to the square root of the sample size. (**B**) Metaplots showing Hel2 binding (relative hits) in either wildtype (wt, green, 2 biological replicates) or *new1Δ* (magenta, 2 biological replicates) background, at 30°C, for those mRNAs within the group of top 1000 highest Hel2-bound mRNAs (1) with C-terminal codons being either strongly affected, mildly affected or not affected by lack of New1.

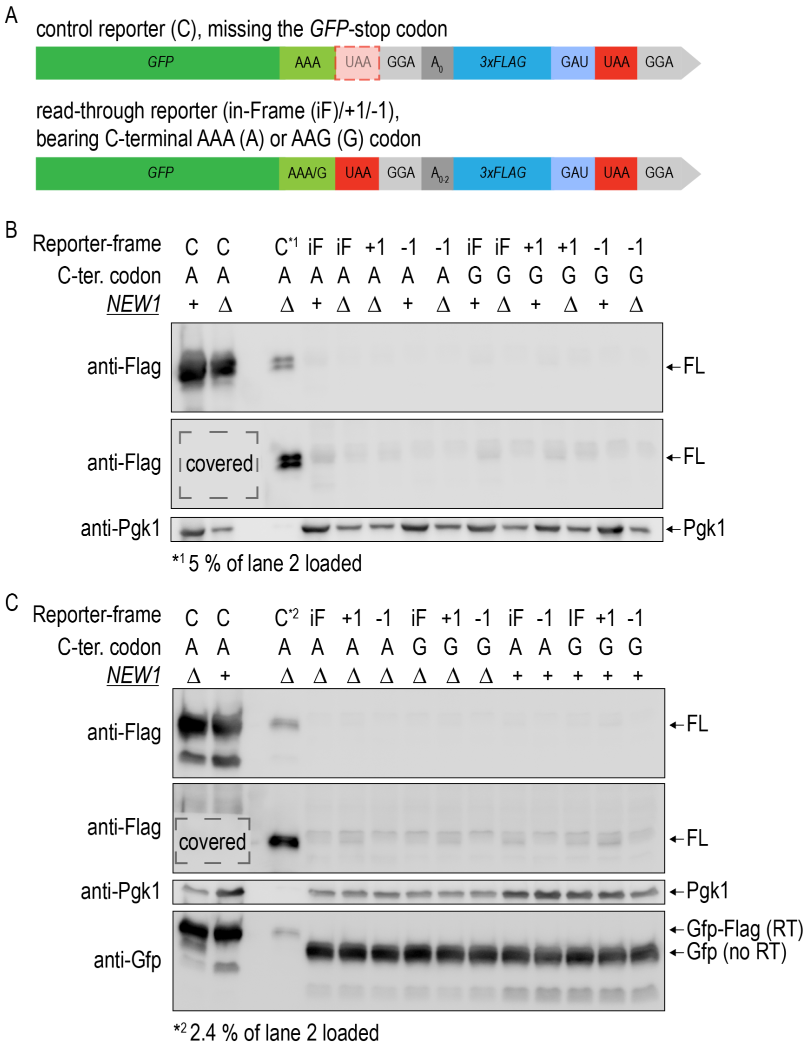

Supplementary Figure S4. Readthrough reporter assay. (**A**) The readthrough reporter consist of the coding sequence of GFP, with either the native lysine codon 'AAA', found to induce queuing upon *new1Δ*, or the non-queuing C-terminal lysine codon 'AAG', followed by the strong stop codon 'UAA'. To avoid sequence variation downstream of the stop codon, a glycine spacer ('GGA') was added. To allow tagging of readthrough products with a 3xFLAG-tag in all three reading frames, three constructs were designed, containing either no, one or two additional nucleotides 'A' for in-frame (iF), +1 or -1 reading frame, respectively. Since the 3xFLAG sequence would end with a lysine codon ('AAG'), an additional aspartic acid ('GAU') spacer was introduced in front of the 3xFLAG stop codon (UAA), followed by a glycine codon ('GGA'). The control construct is identical to the described reporters, except that it misses the GFP stop codon. Consequently, each ribosome is capable of carrying out translation to the end of the 3xFLAG-tag. (**B**) Western blot analysis of GFP-3xFLAG-tagged readthrough reporter protein in *new1Δ* (*NEW1*: Δ) and wildtype (*NEW1*: +) strains harbouring either the control construct ('C') or constructs with the 3xFLAG-tag in-frame (iF), in the +1 shifted frame (+1) or in the -1 shifted frame (-1), with a C-terminal lysine codon 'AAA' ('A') or 'AAG' ('G'). Protein extract from equal amounts of cells was loaded per lane. In the second panel, the control samples were covered during visualization. Indices show the relative loading compared to lane 2 (**B**) or lane 1 (**C**). In (**C**) the Gfp signal is visualized in addition and sizes of Gfp-Flag with readthrough (RT) or without readthrough (no-RT) are indicated.

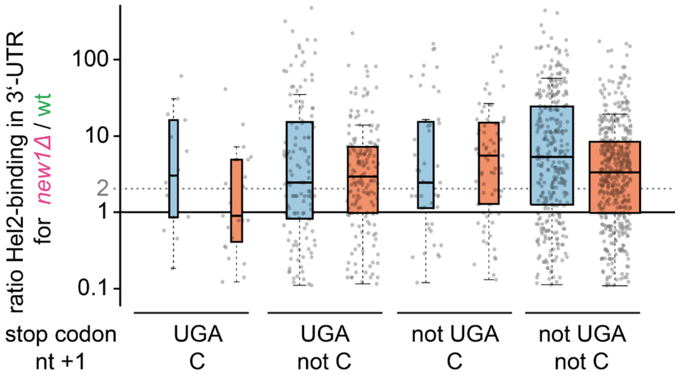

Supplementary Figure S5. Boxplot analysis of average Hel2 3'-UTR enrichment for mRNAs, containing strongly affected C-terminal codons at 20°C (blue, data from 2 biological replicates combined) and 30°C (red, data from 2 biological replicates combined), depending on stop codon context. mRNAs were sorted into the following groups: (1) weak stop codon 'UGA', followed by nucleotide 'C' at position +1; (2) weak stop codon 'UGA' and a nucleotide 'A', 'G', or 'U' a position +1, (3) stop codon 'UAA' or 'UAG', followed by nucleotide 'C' at position +1; (4) stop codon 'UAA' or 'UAG' and a nucleotide 'A', 'G', or 'U' a position +1. Boxplot description: Center lines: medians, box limits: 25th and 75th percentiles, whiskers: 1.5x interquartile range from 25th - 75th percentile. All data points are represented by dots. Width of the boxes is proportional to the square root of the sample size.

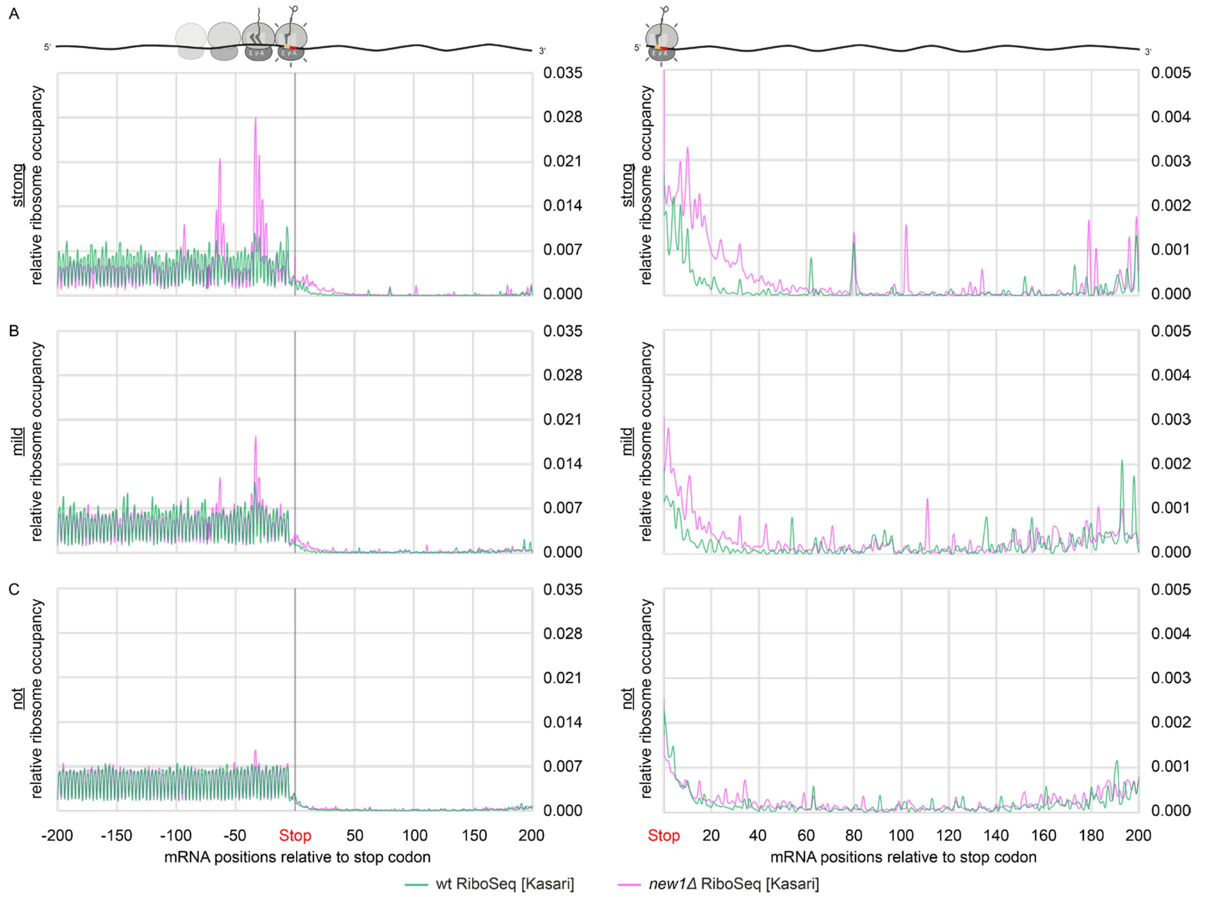

Supplementary Figure S6. Re-analysis of published RiboSeq data from *new1Δ*, comparing to wildtype (2), focusing on 200 nt upstream and downstream of the stop codon. P-site positions are shown. Metagene plots are shown for the (**A**) strongly affected, (**B**) mildly affected, and (**C**) non-affected groups of mRNAs. Full metaplots are shown on the left and only 3′-UTR on the right. Metaplots do show a relative increase in occupancy within the 3′-UTR for New1-lacking strains in the strongly and mildly affected groups of mRNAs, but did not reveal any convincing 3-nt periodicity within the 3′-UTR.

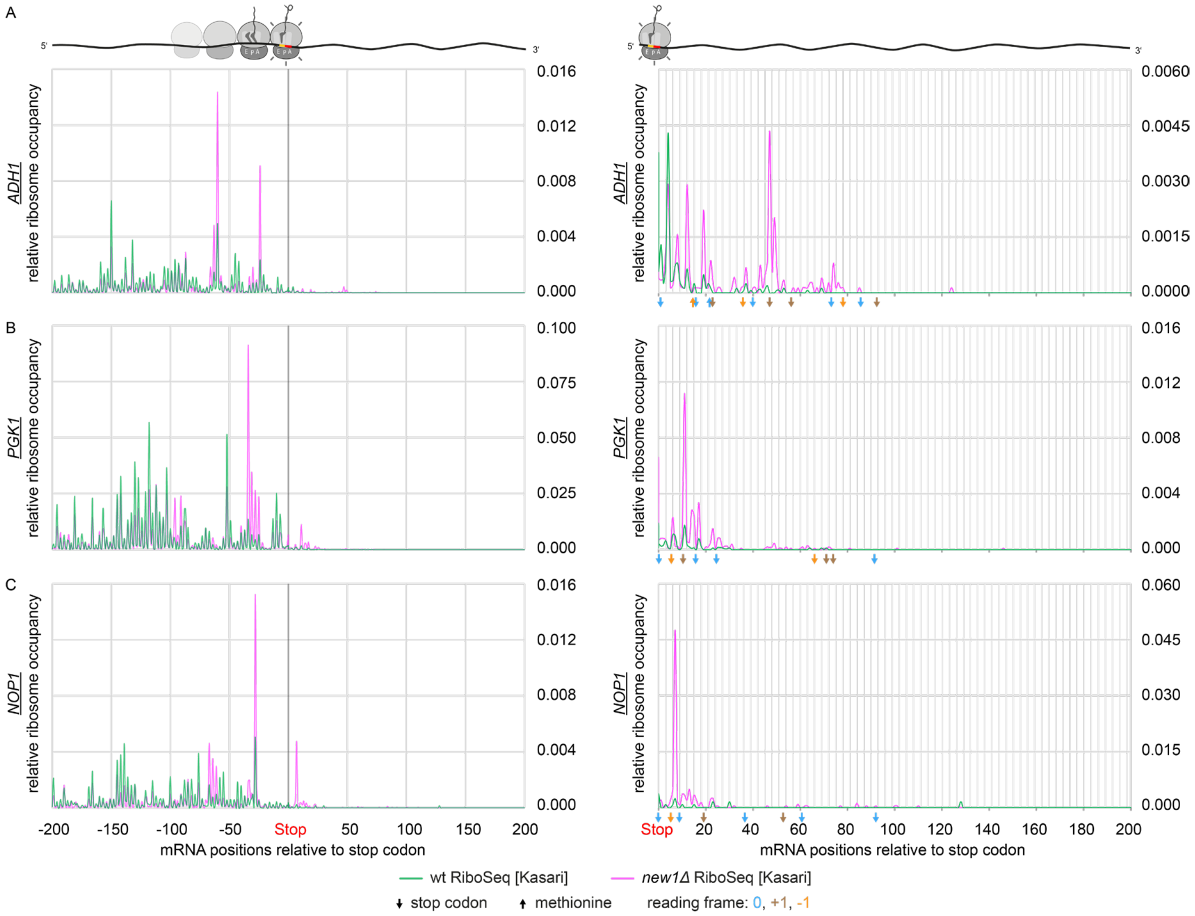

Supplementary Figure S7. Re-analysis of published RiboSeq data from *new1Δ*, comparing to wildtype (2), focusing on 200 nt upstream and downstream of the stop codon. P-site positions are shown. Single plots are shown for the strongly affected mRNAs (**A**) *ADH1*, (**B**) *PGK1* and (**C**) *NOP1*. Full plots are shown on the left and only 3′-UTR on the right. Positions of stop codons (↓) and of methionine codons (↑ as potential re-initiation sites) are shown by arrows in different colours, indicating different reading frames. In the right panel, grid lines represent in-frame steps of 3 nt. A convincing 3′-periodicity was not detected in the 3′-UTR.

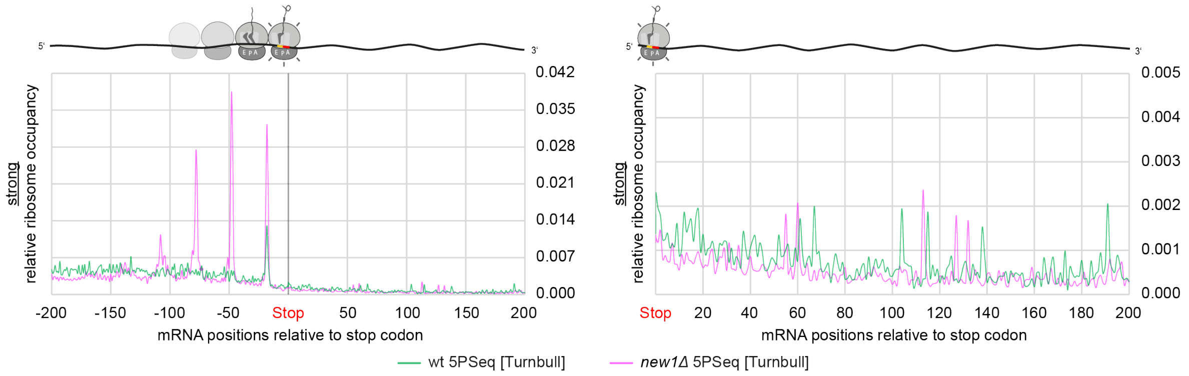

Supplementary Figure S8. Re-analysis of published 5PSeq data (3), focusing on 200 nt upstream and downstream of the stop codon. 5′-end positions are shown. Metagene plots are shown for the strongly affected group of mRNAs. Full metaplots are shown on the left and only 3′-UTR on the right. Metaplots do not reveal any additional 5′-end density in *new1Δ*, and no convincing 3-nt periodicity within the 3′-UTR.

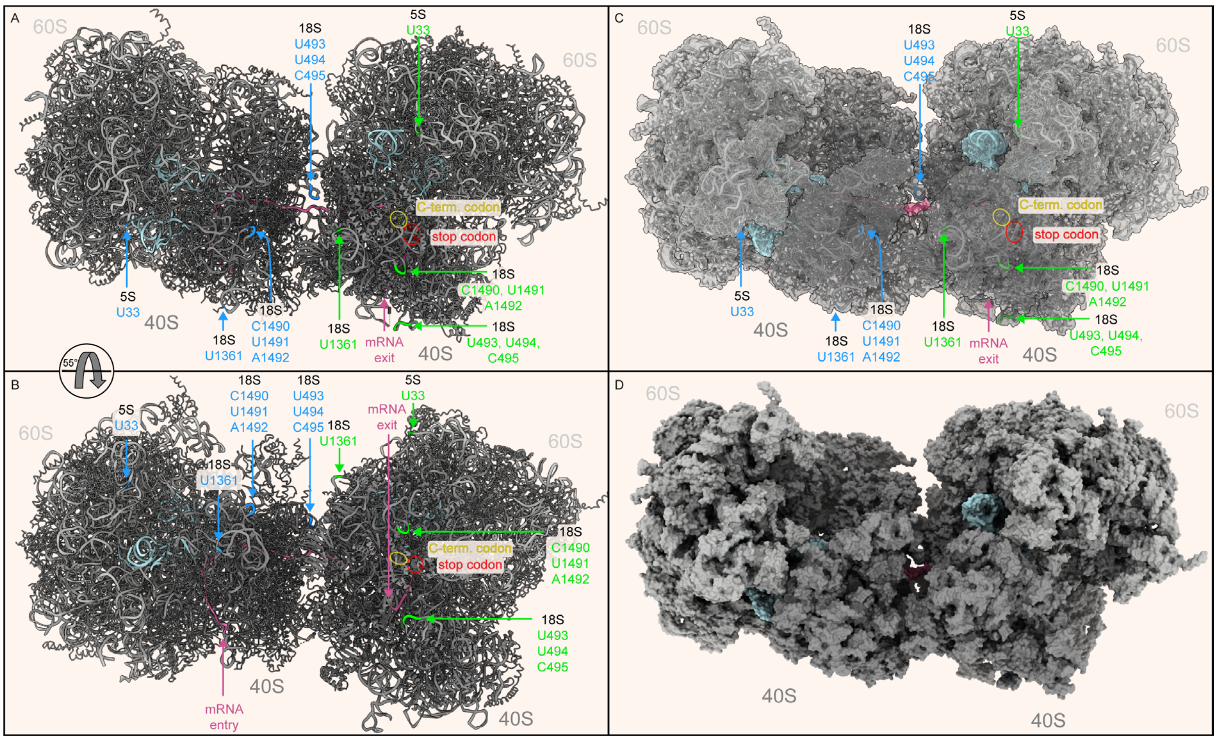

Supplementary Figure S9. Crosslink sites of Hel2 in 18S and 5S rRNA, determined from published CRAC data (1) positioned on a published disome structure (pdb_00006i7o (4)). Hel2 crosslinking sites are indicated in green for the stalled ribosome, and in blue for the collided ribosome. mRNA is shown in magenta. tRNAs are shown in cyan. Hypothetical positions of stop codon (red) and C-terminal codon (yellow) are indicated for the case that the stalled ribosome is positioned with the stop codon in the A-site, as previously indicated for ribosomes stalled in *new1Δ* by RiboSeq (2) and 5PSeq (3). Ribosomal proteins and RNA are shown in grey. (**A**, **B**) ribbon model, tilted by a 55° angle to visualize mRNA exit and mRNA entry sites, (**C**) transparent surface model, (**D**) surface model.

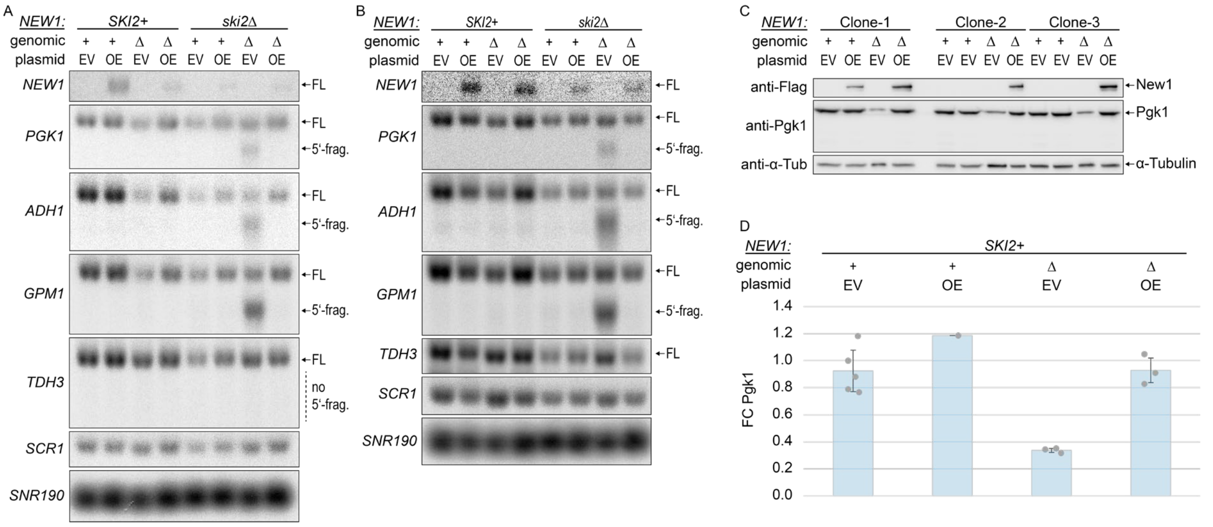

Supplementary Figure S10. Further replicates for study of NGD fragments at 20°C. Related to **Figure 2.** (**A, B**) Northern blot analysis of RNA levels for *NEW1*, *PGK1*, *ADH1*, *GPM1*, *TDH3*, *SCR1*, and *SNR190* in *new1Δ* (genomic Δ), *NEW1*-positive (genomic +) strains, containing an empty vector (plasmid EV) or New1-overexpression vector (plasmid OE), and containing or lacking the *SKI2* gene. Blots for replicates 2 and 3 (quantified in **Figure 2B**). (**C**) Western blot analysis of Pgk1 levels in *new1Δ* (genomic Δ; plasmid EV), wildtype-like (genomic +; plasmid EV), and New1-overexpressing (genomic + or genomic Δ; plasmid OE) strains, compared to wildtype strains containing an empty vector (genomic +, plasmid EV). (**D**) Quantification of protein levels for Pgk1, relative to α-Tubulin, related to (**C**), n = 3 biological replicates for genomic Δ; plasmid EV and genomic Δ; plasmid OE, n = 5 biological replicates for genomic + ; plasmid EV, n = 1 biological replicate for genomic +; plasmid OE. All data quantified as fold-change (FC) compared to median of all genomic + values. Error bars represent 1 standard deviation.

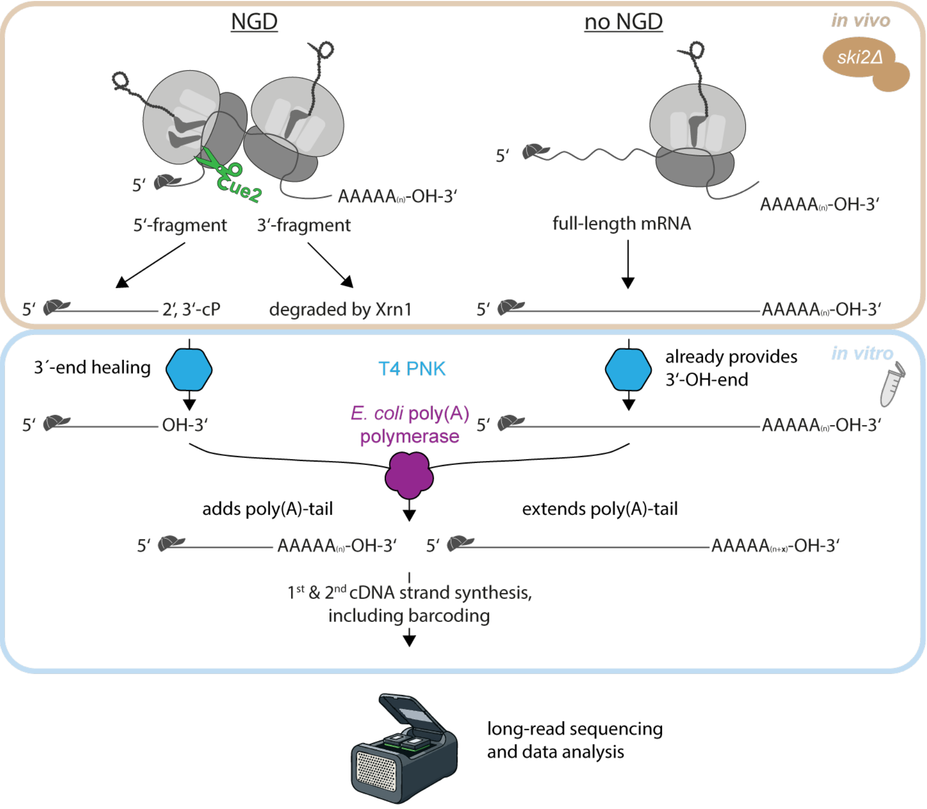

Supplementary Figure S11. Schematic representation of endogenous processing (top) and library preparation (bottom) for NGD cleavage fragments in Ski2-lacking strains, for nanopore sequencing analysis. Top: During NGD, target mRNAs are cleaved by Cue2, which leaves 2′-3′-cyclic phosphates at the 3′-end of the 5′-fragment, and 5′-fragments that are consecutively degraded by Xrn1. In a Ski2-lacking strain, the 5′-fragment is stabilized, but lacks a poly(A)-tail. To enable addition of poly(A) to allow for 1st strand cDNA synthesis employing oligo(dT) primers, RNA is polyadenylated *in vitro* using *E. coli* poly(A) polymerase. This step adds a poly(A)-tail to cleavage fragments and extends existing poly(A)-tails.

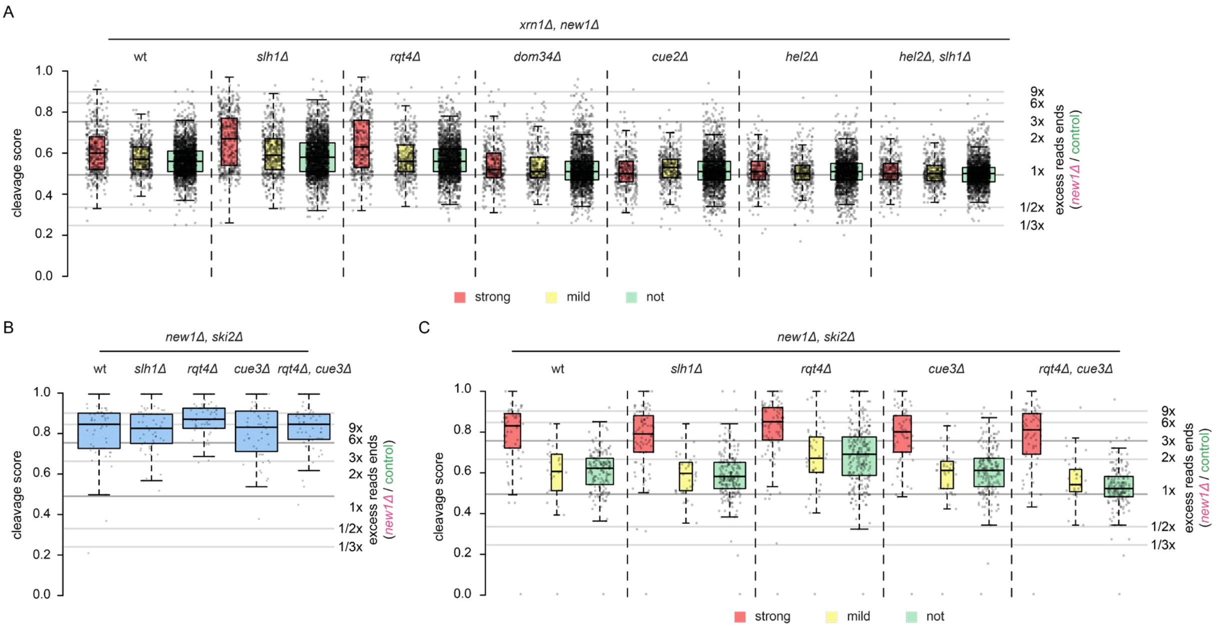

Supplementary Figure S12. Comparison of cleavage scores for different groups of mRNA over different strains. (**A**) Cleavage scores of strongly, mildly, or non-affected mRNAs compared over all different strains lacking Xrn1 in combination with New1 (and additional factors). Cleavage scores of (**B**) all mRNAs called as NGD candidates, or (**C**) of strongly, mildly, or non-affected mRNAs in strains lacking Ski2. Cleavage score of 0.5 corresponds to a ratio of cleavage fragments of 1 (comparing strains lacking New1 and corresponding controls not lacking New1), meaning equal (normalized) number of read ends. A cleavage score of 0.75 (threshold for the cleavage score, 1st criterium for calling NGD candidates) corresponds to 3-fold excess of cleavage fragments in the New1-lacking strains compared to matched controls not lacking New1. Boxplot description: Center lines: medians, box limits: 25th and 75th percentiles, whiskers: 1.5x interquartile range from 25th - 75th percentile. All data points are represented by dots. Width of the boxes is proportional to the square root of the sample size.

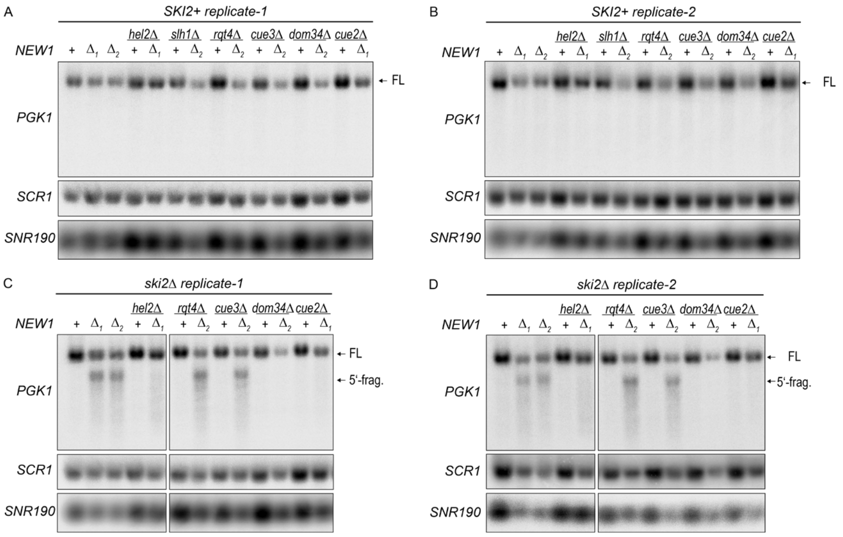

Supplementary Figure S13. Northern blots for study of NGD fragments in mutants lacking additional NGD or RQT factors, at 20°C. (**A**,**B**) in *SKI2* containing strains, (**C**,**D**) in *ski2Δ* strains. Δ_1 designates mutants in which the *NEW1* gene was replaced with a KanMX cassette, Δ_2 designates mutants in which the *NEW1* gene was replaced with a HphMX cassette. FL: full-length; 5′-frag.: endonucleolytic 5′-fragment. For **C** and **D**, different parts of the same blot (from the same raw images) were combined, as indicated by frames.

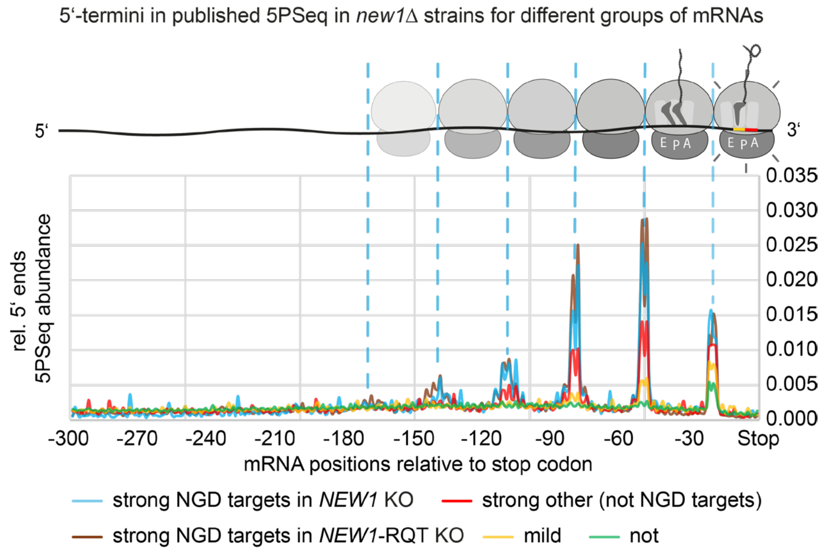

Supplementary Figure S14. 5PSeq (3) analysis of different groups of mRNAs. Strongly affected mRNAs called as NGD targets in the *NEW1* deletion strain without additional deletion of RQT factors, strongly affected mRNAs called as NGD targets only upon additional deletion of RQT factor(s), strongly affected mRNAs not called as NGD targets, mildly affected and non-affected mRNAs. Relative abundance of 5′-ends is shown as unsmoothed data, related to **Figure 5B**, where data are shown smoothed with a mean filter with a 5 nt window.

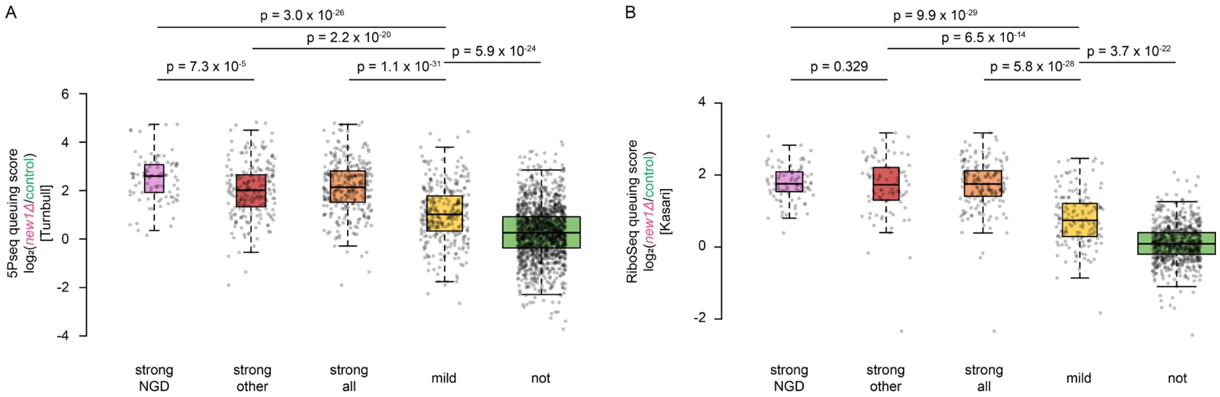

Supplementary Figure S15. Boxplot analysis of queuing scores from published (**A**) 5PSeq (3) and (**B**) RiboSeq (2) in strains lacking *NEW1* compared to wildtype strain for different groups of mRNAs: strongly affected mRNAs called as NGD targets, other strongly affected mRNAs not called NGD targets, all strongly affected mRNAs, mildly and non-affected mRNAs. Queuing scores were calculated following pipelines described in the respective publications. p-values were calculated using Student's t-test (2-tailed, heteroscedastic). Boxplot description: Center lines: medians, box limits: 25th and 75th percentiles, whiskers: 1.5x interquartile range from 25th - 75th percentile. All data points are represented by dots. Width of the boxes is proportional to the square root of the sample size.

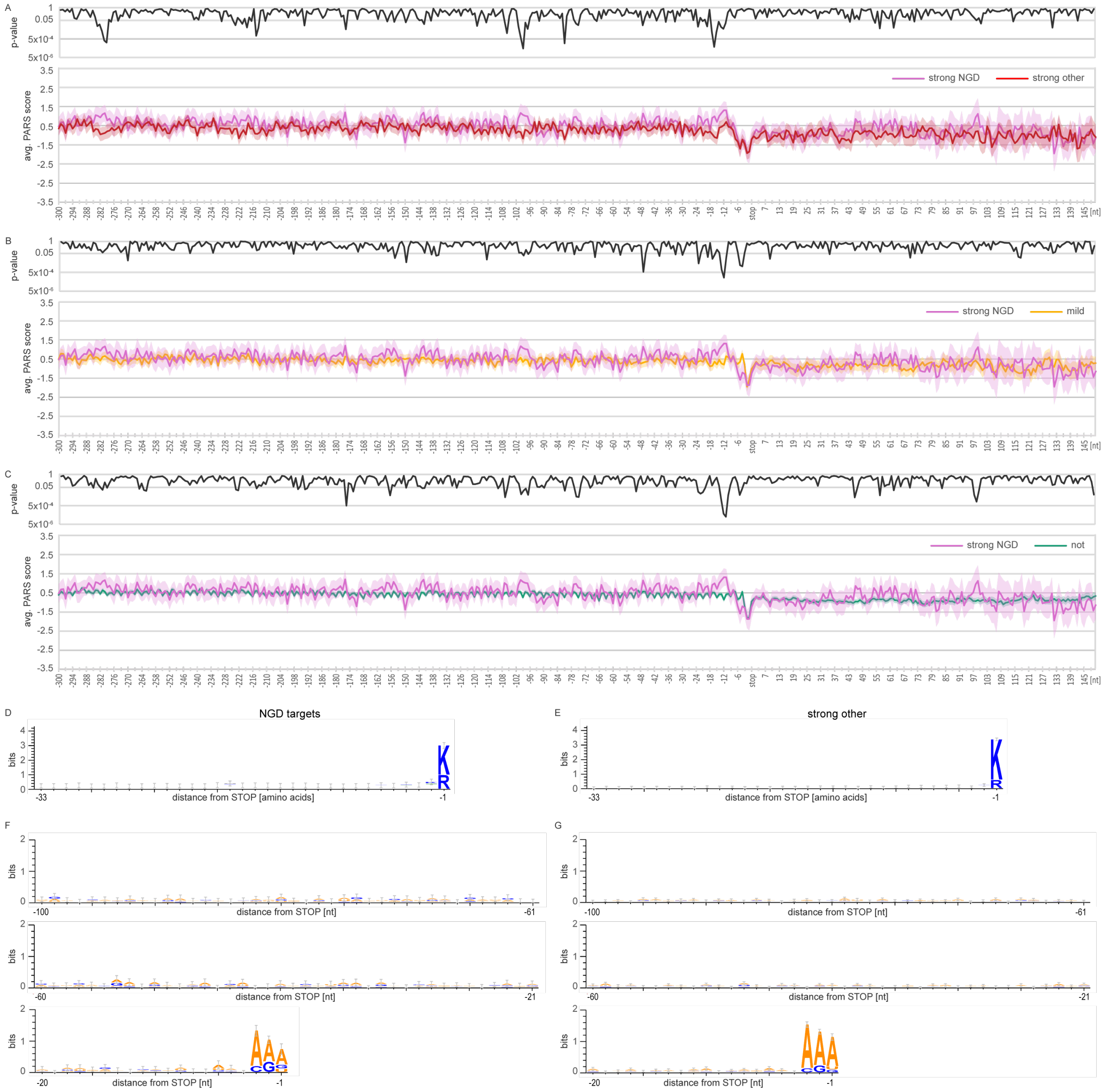

Supplementary Figure S16. Related to **Figure 5D**. Analysis of folding propensity (**A-C**) and sequence motifs (**D-G**) comparing different groups of mRNAs. (**A-C**) Published (5) position-specific PARS score (average across all mRNAs of each group) and 95% confidence intervals are shown for the last 300 nt of the coding sequence (including stop codon) and first 150 nt of the 3′-UTR. p-value (Student′s t-test is also shown to approximate the significance of differences at each position). The following groups were compared: (**A**) strongly affected mRNAs called as NGD targets *versus* other strongly affected mRNAs not called as NGD target, (**B**) strongly affected mRNAs called as NGD targets *versus* mildly affected mRNAs, (**C**) strongly affected mRNAs called as NGD targets *versus* non-affected mRNAs. (**D-G**) Amino acid (last 33 amino acids of each encoded protein; **D,E**) or nucleotide sequence (last 100 nt of the coding sequence, excluding stop codon; **F,G**) were tested for motifs using WebLogo 3 (6) and resulting sequence logos are plotted for strongly affected mRNA called as NGD targets (**D,F**) or other strongly affected mRNAs, not called as NGD targets (**E,G**). Except for conserved last amino acids (K or R) and last codons (′AAA′, ′AGG′ or ′CGU′), no sequence motif emerged, but in the class of NGD-targets, the arginine codons ′AGG′ and ′CGU′ were more strongly represented than in the class of mRNAs not called as targets, where lysine codon ′AAA′ was more dominant.

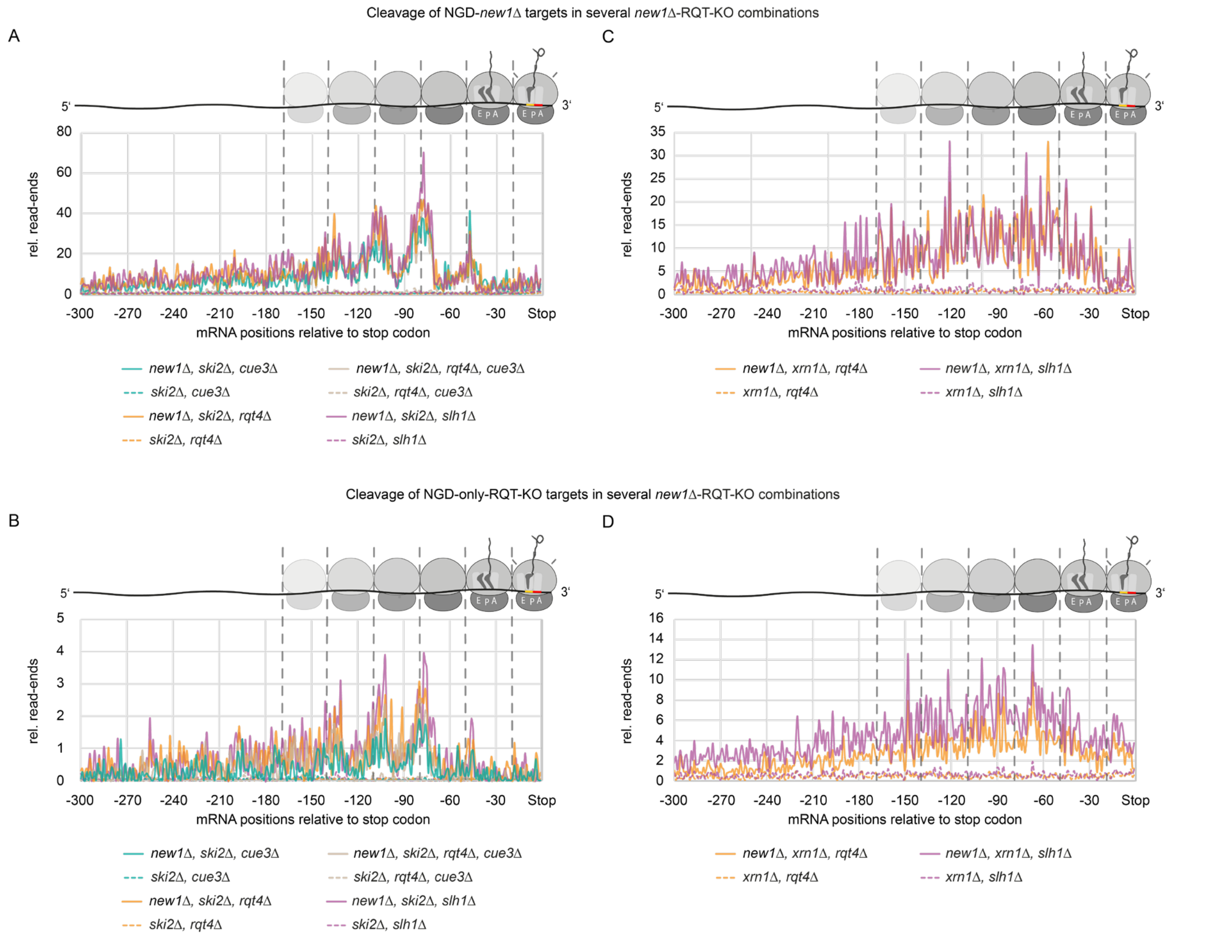

Supplementary Figure S17. Analysis of endonucleolytic fragments by nanopore sequencing in RQT deficient yeast strains. (**A**) Relative distribution of nanopore sequencing 3′-read ends across the last 300 nt upstream of the 3′-UTR for genes targeted by NGD in the absence of New1 alone or (**B**) upon additional loss of RQT factors, in multiple RQT deficient strains, in the absence of *SKI2*. (**C**) Nanopore 5′-read-end analysis using strains lacking Xrn1 across the last 300 nt upstream of the 3′-UTR for genes targeted by NGD in the absence of New1 alone or (**D**) upon additional loss of RQT factors, in multiple RQT deficient strains, in the absence of *SKI2*. Queued ribosomes are indicated by dashed, grey lines based on *new1Δ* 5PSeq data (3) (see **Supplementary Figure S14**). Strains lacking *SKI2* n=2, strains lacking *XRN1* n=3. Related to **Figure 6B,C**.

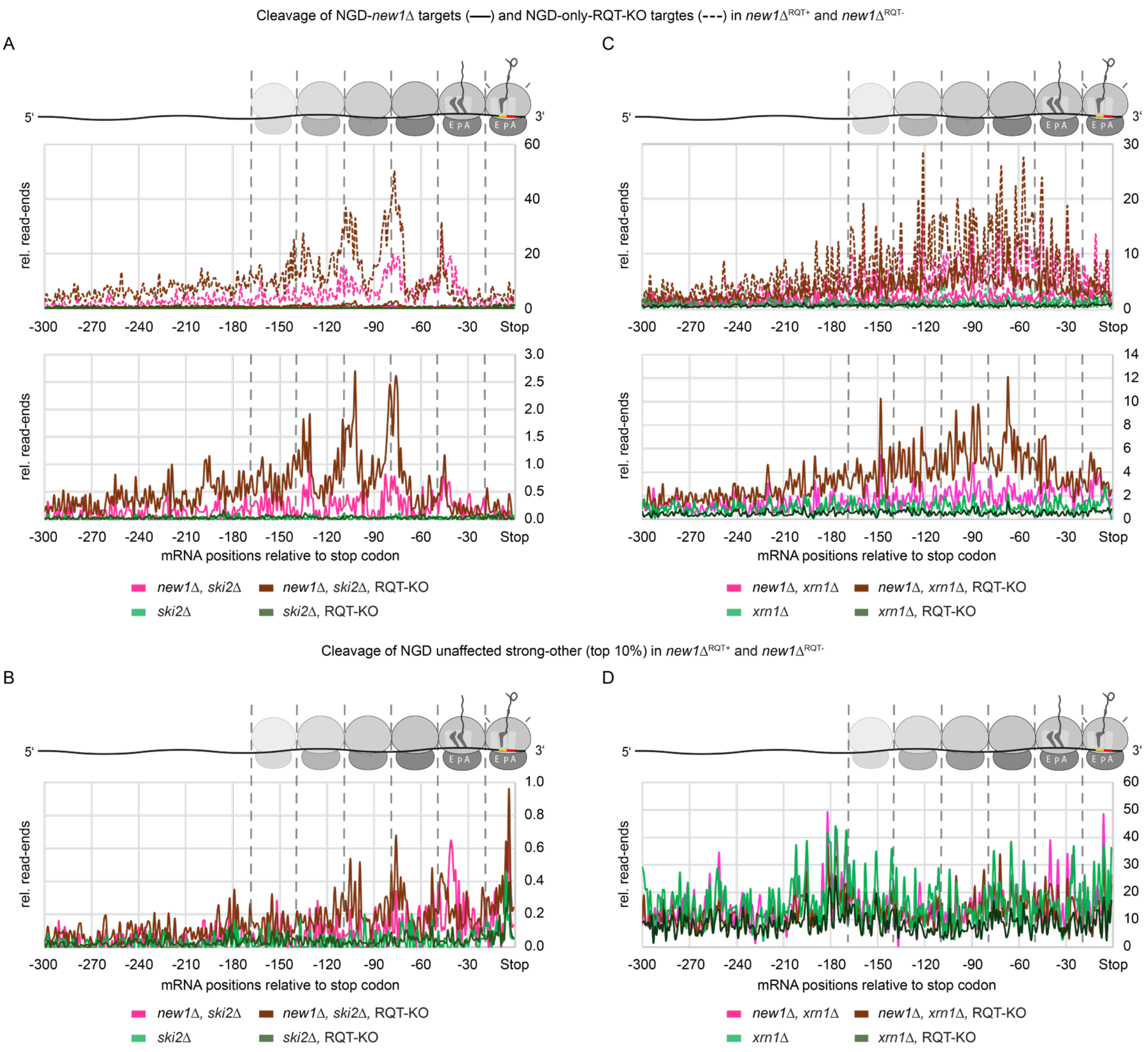

Supplementary Figure S18. Comparison of relative nanopore sequencing read ends across the last 300 nt upstream of the 3′-UTR of (**A**) genes defined as NGD targets in the absence of New1 (dashed line) and genes targeted by NGD only after additional loss of RQT factors (solid line) in strains lacking *new1Δ, ski2Δ* (RQT+) or with additional loss of RQT factors (RQT-). (**B**) Control: the top 10% most abundant mRNAs from the group of strongly affected mRNAs not called as NGD targets (**C**, **D**) Same comparison for strains lacking *XRN1* instead of *SKI2*. Queued ribosomes are indicated by dashed, grey lines based on *new1Δ* 5PSeq data (3) (see **Supplementary Figure S14**). Strains lacking *SKI2* n=2, strains lacking *XRN1* n=3, except *xrn1Δ* and *new1Δ, xrn1Δ* n=2. Data from several strains combined for the RQT-KO group.

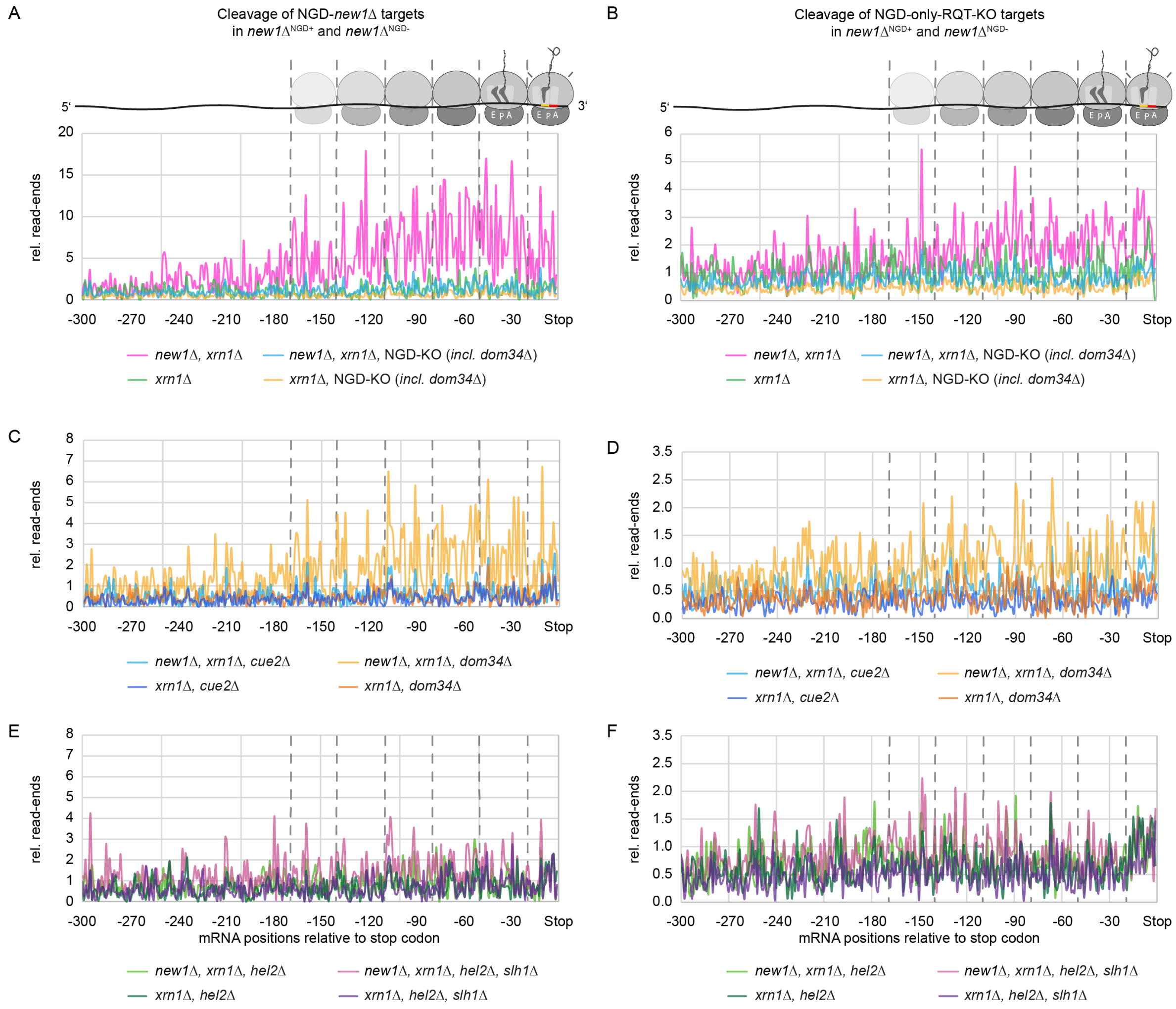

Supplementary Figure S19. Analysis of relative nanopore sequencing read ends of NGD targets in NGD mutants. (**A**) Comparison of relative 5′-ends across the last 300 nt upstream of the 3′-UTR for genes targeted by NGD in the absence of New1 alone or (**B**) upon additional loss of RQT factors, in strains lacking *NEW1* and *XRN1* (NGD+) or with additional loss of NGD factors (NGD-). Strains lacking *DOM34*, *CUE2*, *HEL2* or *HEL2+SLH1* were grouped to define the NGD-knockouts (NGD-KO) class. (**C**) Detailed comparison of strains lacking *CUE2* or *DOM34* in the absence of *XRN1* and with or without *NEW1* for NGD targets detected in *new1Δ* or (**D**) NGD targets detected only upon loss of RQT factors. (**E**,**F**) Analogous comparison for strains additionally lacking *HEL2* or *HEL2+SLH1*. Queued ribosomes are indicated by dashed, grey lines based on *new1Δ* 5Pseq data (3) (see **Supplementary Figure S14**). Strains lacking *XRN1* n=3, except *xrn1Δ* and *new1Δ, xrn1Δ* n=2. Data from four strains combined for the NGD-KO group.

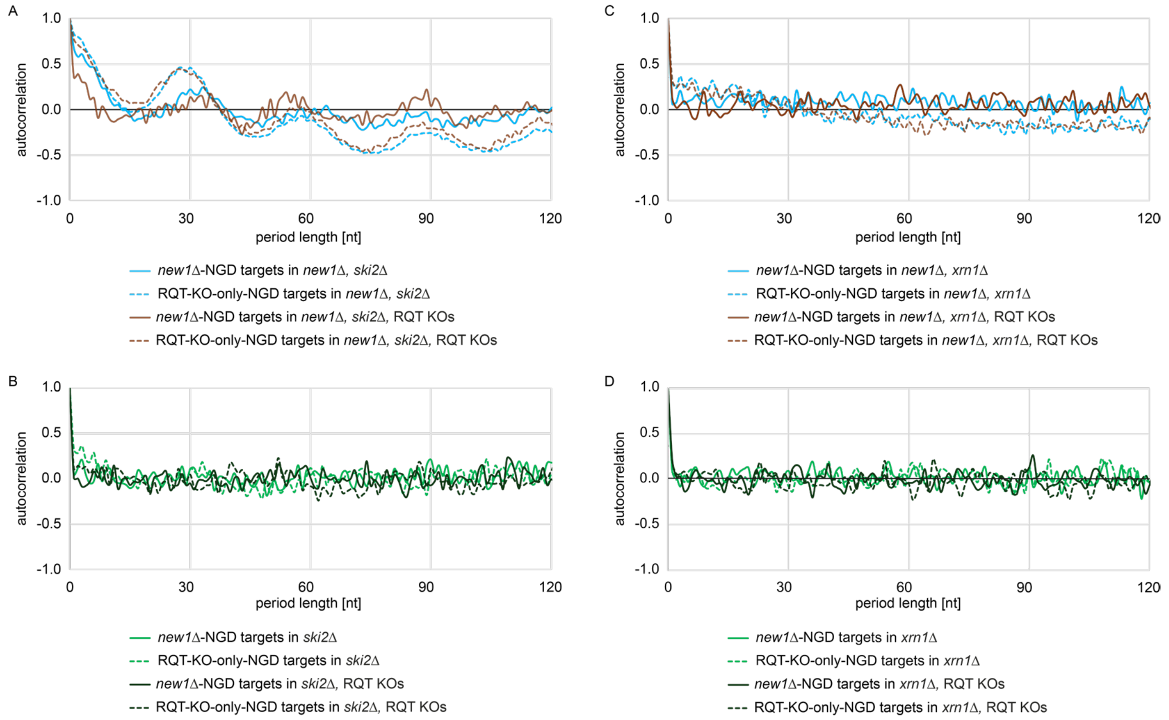

Supplementary Figure S20. Analysis of periodicity *via* autocorrelation for NGD targets detected in *new1Δ* and NGD targets detected only upon loss of RQT factors, in strains lacking *NEW1* and *SKI2*, and corresponding strains with the additional loss of RQT factors (**A**), controls bearing genomic *NEW1* (**B**). Analogous analyses for *XRN1* knockouts (in place of *SKI2* are shown (**C**) with their corresponding controls (**D**). Strains lacking *SKI2* n=2, strains lacking *XRN1* n=3, except *xrn1Δ* and *new1Δ, xrn1Δ* n=2. Data from several strains combined for the RQT-KO group.

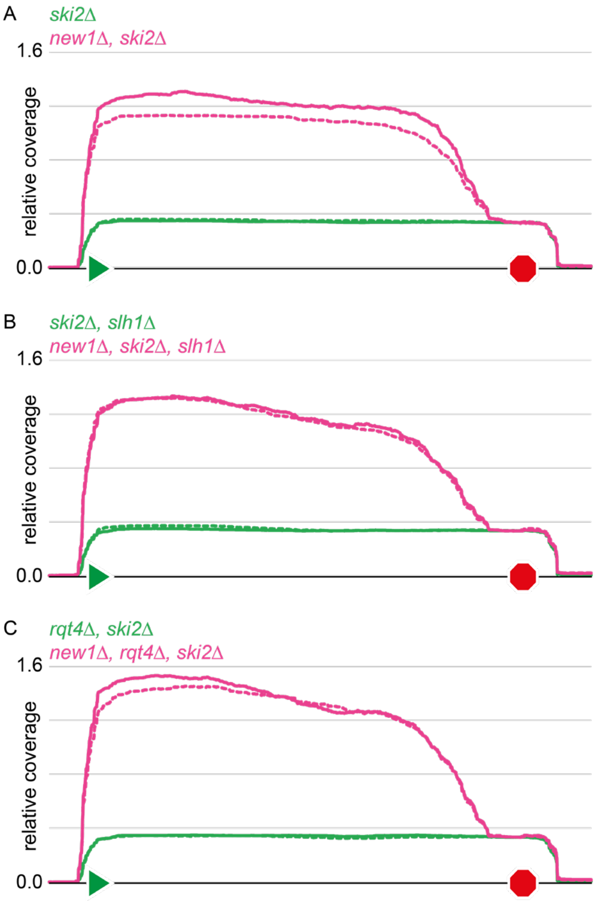

Supplementary Figure S21. Relative nanopore sequencing read coverage along the NGD target *ADH1* for strains with or without *NEW1* and in the absence of *SKI2* (**A**) and for additional loss of the RQT factor *SLH1* (**B**) or *RQT4* (**C**), *CUE3* (**D**) or *RQT4*+*CUE3* (**E**). Each condition includes two biological replicates (dashed and solid line). The start codon is indicated by a green triangle and the stop codon by a red octagon.

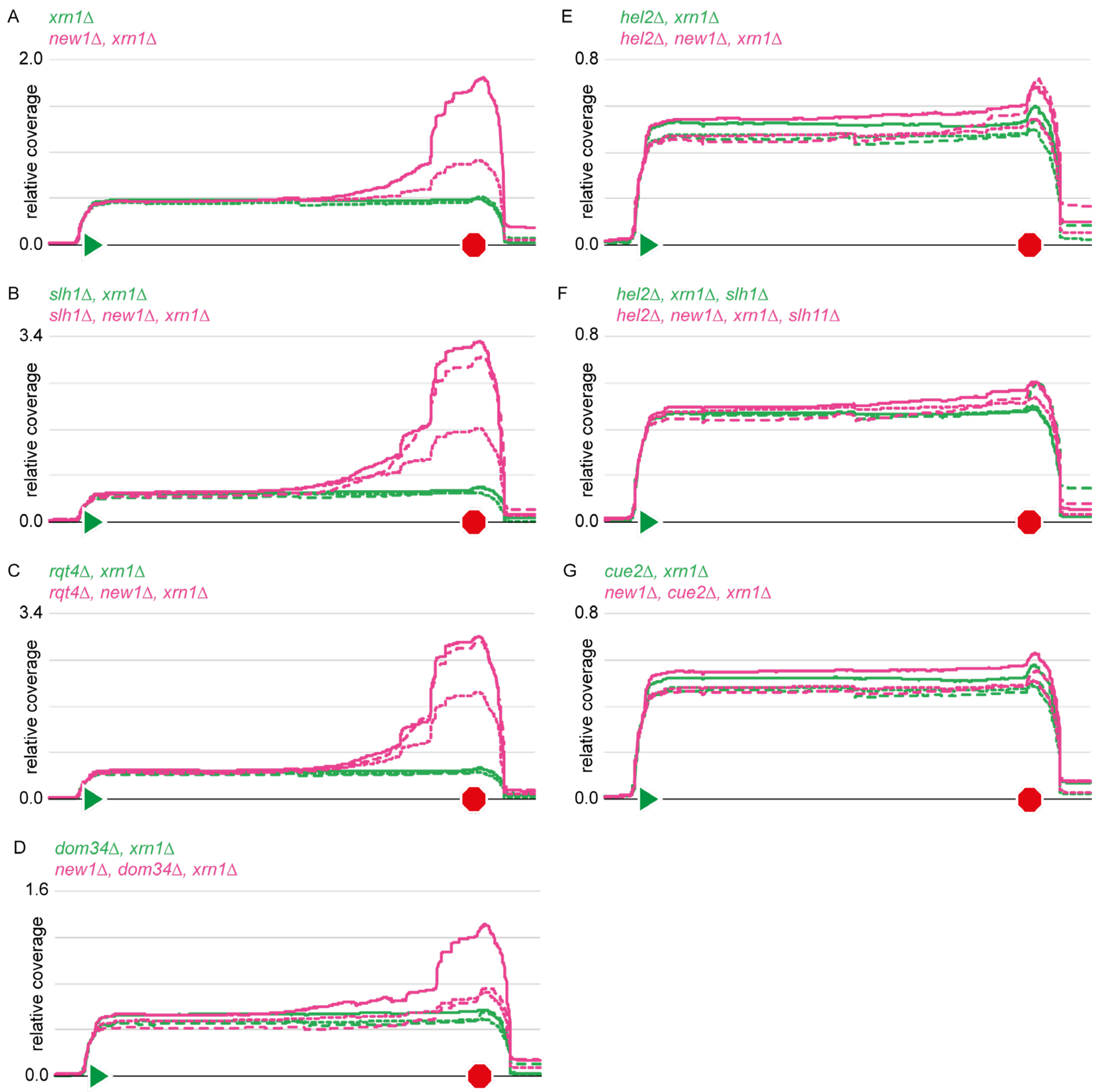

Supplementary Figure S22. Relative nanopore sequencing read coverage along the NGD target *ADH1* in strains with or without *NEW1* and in the absence of *XRN1* (**A**) and for additional loss of the RQT factor *SLH1* (**B**) or *RQT4* (**C**) or NGD factor *DOM34* (**D**), *HEL2* (**E**), *HEL2* and RQT factor *SLH1* (**F**) or *CUE2* (**G**). Each condition includes three biological replicates (dotted, dashed and solid line) except *new1Δ, xrn1Δ and xrn1Δ*, here two replicates are used. The start codon is indicated by a green triangle and the stop codon by a red octagon.

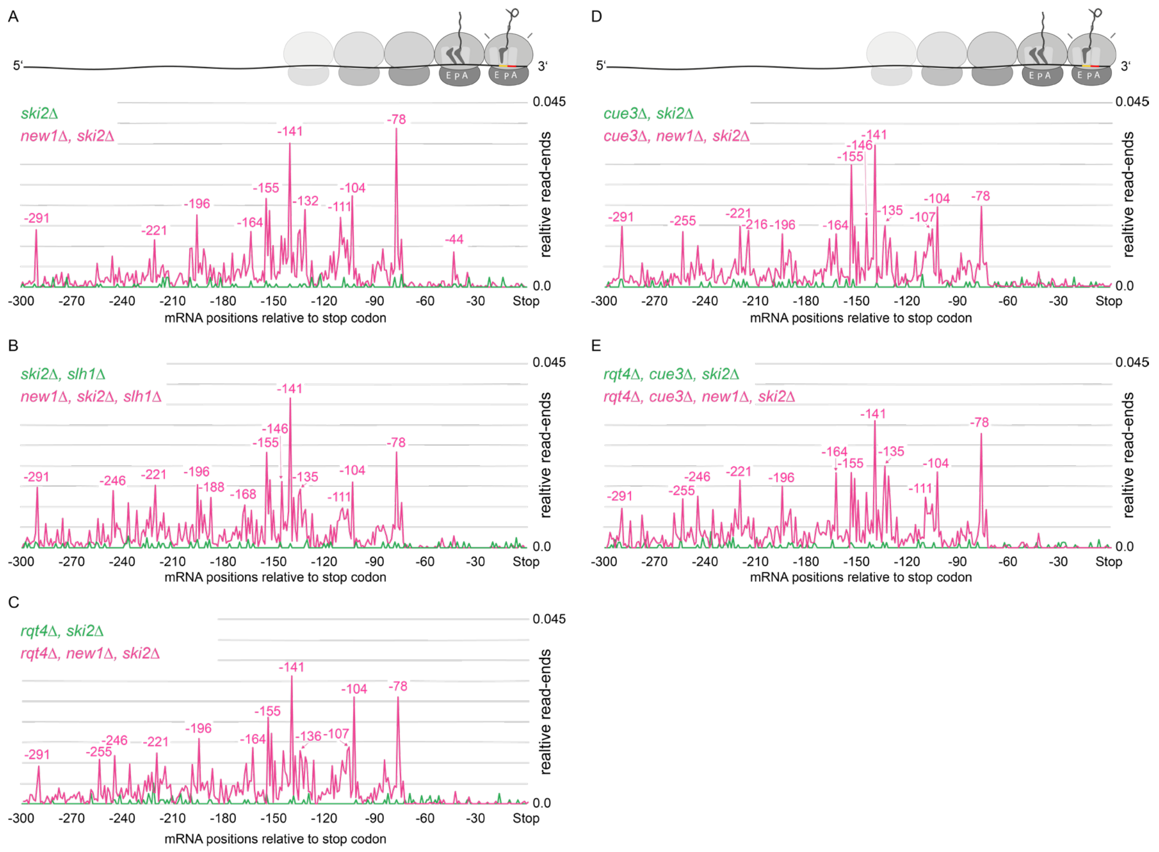

Supplementary Figure S23. Relative nanopore sequencing 3′-read ends along the last 300 nt upstream of the 3′-UTR of the NGD target *ADH1* with or without *NEW1* and in the absence of *SKI2* (**A**) and additional loss of the RQT factor *SLH1* (**B**) or *RQT4* (**C**), *CUE3* (**D**) or *RQT4+CUE3* (**E**). Queued ribosomes are schematically represented at the top, based on *new1Δ* 5PSeq data (3) (see **Supplementary Figure S14**). Each condition includes two biological replicates (data pooled).

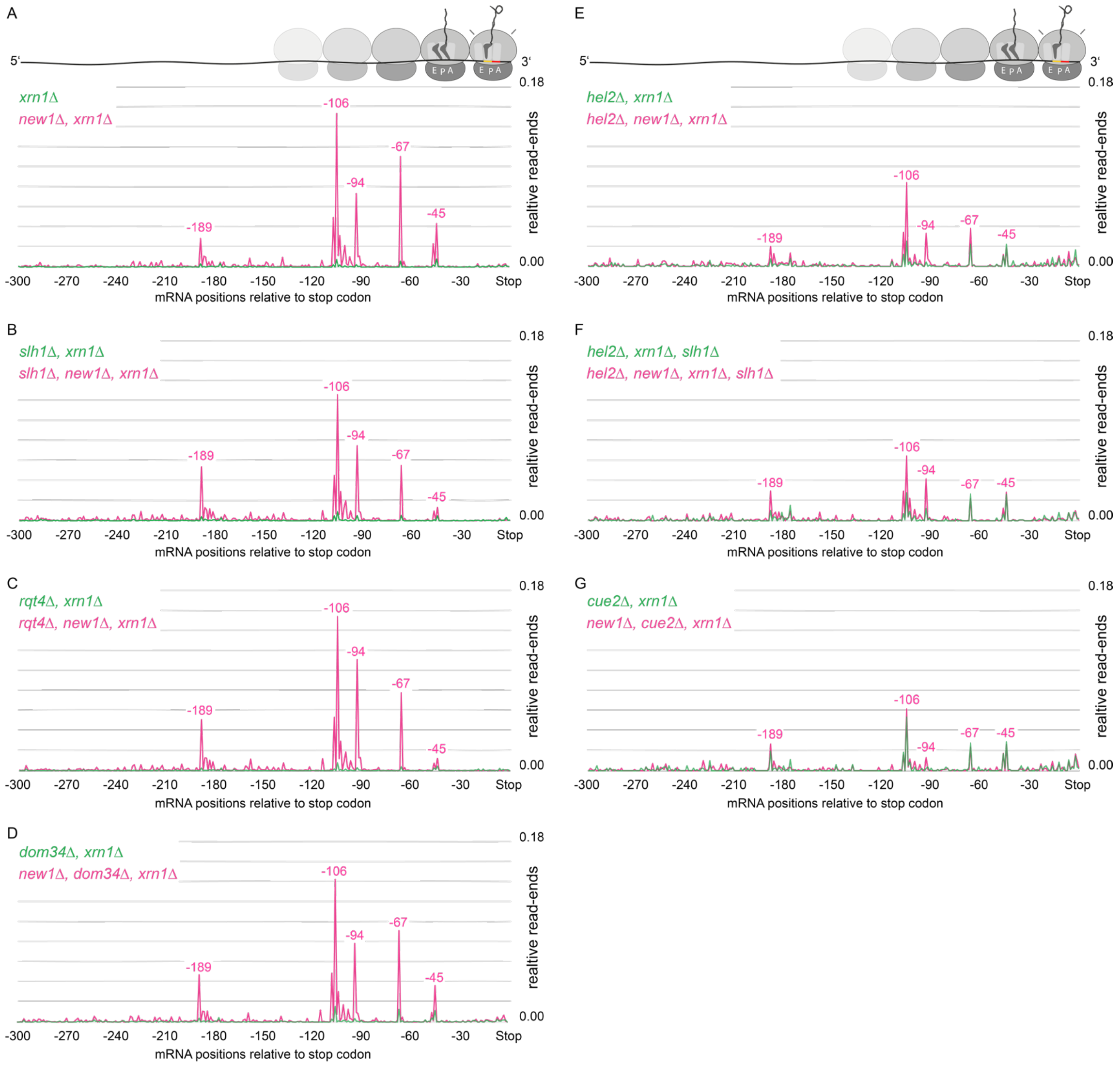

Supplementary Figure S24. Relative nanopore sequencing 5′-read ends for the NGD target *ADH1* along the last 300 nt upstream of the 3′-UTR with or without *NEW1* and in the absence of *XRN1* (**A**) and for the additional loss of the RQT factor *SLH1* (**B**) or *RQT4* (**C**) or the additional loss of the NGD factor *DOM34* (**D**), *HEL2* (**E**), *HEL2* and the RQT factor *SLH1* (**F**) or *CUE2* (**G**). Queued ribosomes are schematically represented at the top based on *new1Δ* 5PSeq data (3) (see **Supplementary Figure S14**). Each condition includes three biological replicates except *new1Δ, xrn1Δ and xrn1Δ*, here two replicates are used (data pooled). Note: panels show relative (normalized) read-end counts per condition. Consequently, low absolute numbers of read-ends can produce visible relative signals if total signals are also low, as, e.g., seen in panel **G** (where NGD endonuclease Cue2 is lacking and signals from New1-lacking strains do not exceed those from controls not lacking New1). Therefore, signals should only be compared within a panel rather than comparing absolute magnitudes between panels.

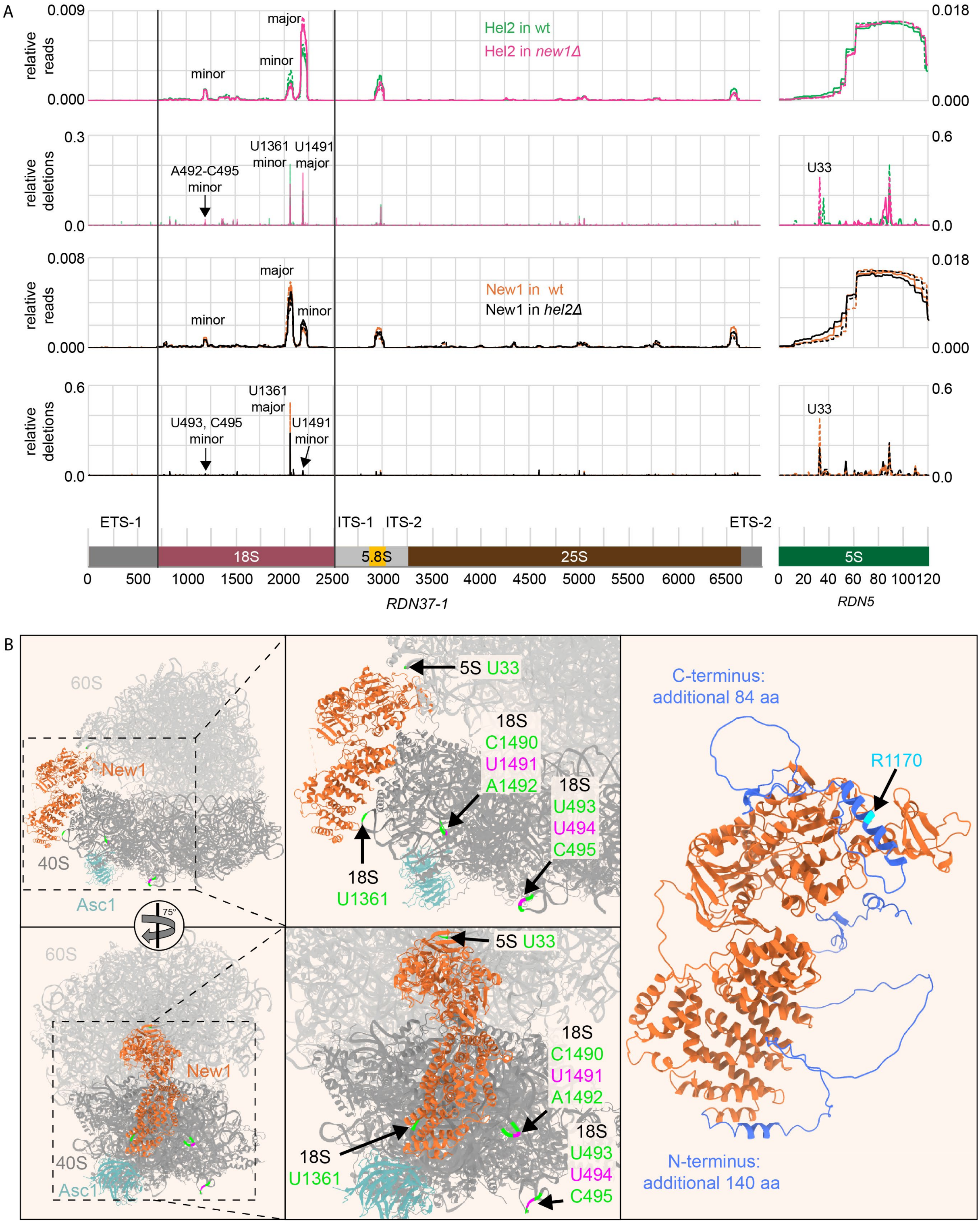

Supplementary Figure S25. (**A**) CRAC data for Hel2 and New1 interactions with ribosomal RNA at 20°C. Reads and deletions mapped to *RDN37-1* (encoding 18S, 5.8S and 25S rRNAs as a single transcript flanked by external and internal transcribed spacers (ETS, ITS, respectively), as well as *RDN5*, encoding 5S rRNA. For *RDN5*, all reads mapped randomly to either *RDN5-1, RDN5-2, RDN5-3, RDN5-4, RDN5-5* or *RDN5-6* were summed up to compensate for differences arising from random mapping. The sum of reads and deletions mapped to one of the two loci was normalized to 1 and relative reads and relative deletions over the respective locus are shown. Note a difference in scaling between the two loci. Major and minor peaks were

determined based on relative reads mapping to the positions. The crosslink positions were determined based on the main deletion peaks mapped in the respective part of the sequence. (**B**) Main deletion peaks mapped to the structure model for New1 bound to a ribosome (pdb: 6S47 (2)) (left and middle panels. Left: full ribosome shown, right: zoom on the New1-bound portion of the ribosome. Colour scheme: green: crosslink sites found in both, Hel2 and New1 CRAC data; Magenta: crosslink sites only found in Hel2 data. Right panel: alphafold (7) model (AF-Q08972-F1) with portion of New1 resolved in the published structure model shown in orange, additional N- and C-terminal amino acids shown in blue. Cyan: Protein-RNA crosslink site within New1 identified by identification of RNA-associated peptides (iRAP) (8).

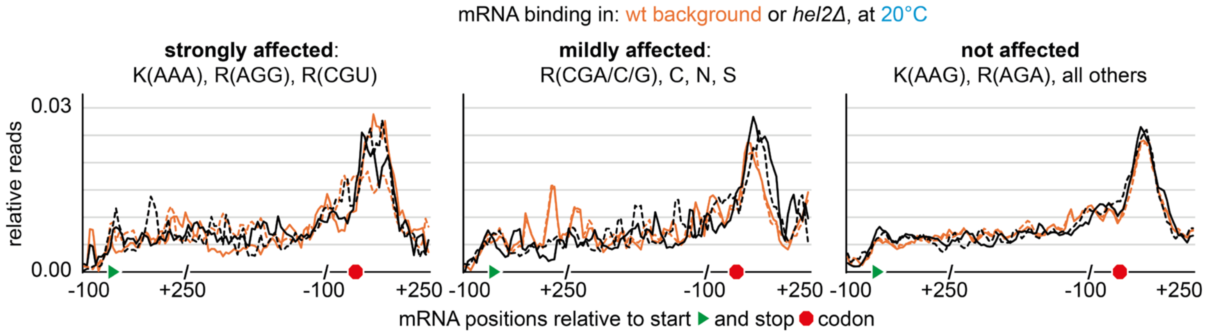

Supplementary Figure S26. New1-HTP-CRAC metaplots showing New1 binding (relative reads) in either wildtype (wt, orange, 2 biological replicates) or *hel2Δ* (black, 2 biological replicates) background, at 20°C, for those mRNAs within the group of top 1000 highest Hel2-bound mRNAs (1) with C-terminal codons being either strongly affected, mildly affected or not affected by lack of New1.

Supplementary Table S1. List of strains used in this study

#	Strain	Genotype	Parental strain	Reference or source
1	BY4741	*MATa*, *his3Δ*, *leu2Δ*, *met15Δ*, *ura3Δ*	-	Ref. (9)
2	*new1Δ::KanMX*	*MATa*, *his3Δ*, *leu2Δ*, *met15Δ*, *ura3Δ*, *new1Δ::KanMX*	BY4741 (1)	this study
3	*new1Δ::HphMX*	*MATa*, *his3Δ*, *leu2Δ*, *met15Δ*, *ura3Δ*, *new1Δ::HphMX*	BY4741 (1)	this study
4	*hel2Δ*	*MATa*, *his3Δ*, *leu2Δ*, *met15Δ*, *ura3Δ*, *hel2Δ*	BY4741 (1)	Winz et al., 2019
5	*hel2Δ*, *new1Δ::KanMX*	*MATa*, *his3Δ*, *leu2Δ*, *met15Δ*, *ura3Δ*, *hel2Δ*, *new1Δ::KanMX*	*hel2Δ* (4)	this study
6	*slh1Δ::KanMX*	*MATa*, *his3Δ*, *leu2Δ*, *met15Δ*, *ura3Δ*, *slh1Δ::KanMX*	BY4741 (1)	this study
7	*slh1Δ::KanMX*, *new1Δ::HphMX*	*MATa*, *his3Δ*, *leu2Δ*, *met15Δ*, *ura3Δ*, *slh1Δ::KanMX*, *new1Δ::HphMX*	*slh1Δ::KanMX* (6)	this study
8	*rqt4Δ::KanMX*	*MATa*, *his3Δ*, *leu2Δ*, *met15Δ*, *ura3Δ*, *rqt4Δ::KanMX*	BY4741 (1)	this study
9	*rqt4Δ::KanMX*, *new1Δ::HphMX*	*MATa*, *his3Δ*, *leu2Δ*, *met15Δ*, *ura3Δ*, *rqt4Δ::KanMX*, *new1Δ::HphMX*	*rqt4Δ::KanMX* (8)	this study
10	*cue3Δ::KanMX*	*MATa*, *his3Δ*, *leu2Δ*, *met15Δ*, *ura3Δ*, *cue3Δ::KanMX*	BY4741 (1)	this study
11	*cue3Δ::KanMX*, *new1Δ::HphMX*	*MATa*, *his3Δ*, *leu2Δ*, *met15Δ*, *ura3Δ*, *cue3Δ::KanMX*, *new1Δ::HphMX*	*cue3Δ::KanMX* (10)	this study
12	*cue2Δ::KanMX*	*MATa*, *his3Δ*, *leu2Δ*, *met15Δ*, *ura3Δ*, *cue2Δ::KanMX*	BY4741 (1)	this study
13	*new1Δ::KanMX*, *cue2Δ::HphMX*	*MATa*, *his3Δ*, *leu2Δ*, *met15Δ*, *ura3Δ*, *new1Δ::KanMX*, *cue2Δ::HphMX*	*new1Δ::KanMX* (2)	this study
14	*dom34Δ::KanMX*	*MATa*, *his3Δ*, *leu2Δ*, *met15Δ*, *ura3Δ*, *dom34Δ::KanMX*	BY4741 (1)	this study
15	*new1Δ::HphMX*, *dom34Δ::KanMX*	*MATa*, *his3Δ*, *leu2Δ*, *met15Δ*, *ura3Δ*, *new1Δ::HphMX*, *dom34Δ::KanMX*	*new1Δ::HphMX* (3)	this study
16	*ski2Δ::LEU2*	*MATa*, *his3Δ*, *leu2Δ*, *met15Δ*, *ura3Δ*, *ski2Δ::LEU2*	BY4741 (1)	this study
17	*new1Δ::KanMX*, *ski2Δ::LEU2*	*MATa*, *his3Δ*, *leu2Δ*, *met15Δ*, *ura3Δ*, *new1Δ::KanMX*, *ski2Δ::LEU2*	*new1Δ::KanMX* (2)	this study
18	*new1Δ::HphMX*, *ski2Δ::LEU2*	*MATa*, *his3Δ*, *leu2Δ*, *met15Δ*, *ura3Δ*, *new1Δ::HphMX*, *ski2Δ::LEU2*	*new1Δ::HphMX* (3)	this study
19	*hel2Δ*, *ski2Δ::LEU2*	*MATa*, *his3Δ*, *leu2Δ*, *met15Δ*, *ura3Δ*, *hel2Δ*, *ski2Δ::LEU2*	*hel2Δ* (4)	this study
20	*hel2Δ*, *new1Δ::KanMX*, *ski2Δ::LEU2*	*MATa*, *his3Δ*, *leu2Δ*, *met15Δ*, *ura3Δ*, *hel2Δ*, *new1Δ::KanMX*, *ski2Δ::LEU2*	*hel2Δ*, *new1Δ::KanMX* (5)	this study
21	*slh1Δ::KanMX*, *ski2Δ::LEU2*	*MATa*, *his3Δ*, *leu2Δ*, *met15Δ*, *ura3Δ*, *slh1Δ::KanMX*, *ski2Δ::LEU2*	*slh1Δ::KanMX* (6)	this study
22	*rqt4Δ::KanMX*, *ski2Δ::LEU2*	*MATa*, *his3Δ*, *leu2Δ*, *met15Δ*, *ura3Δ*, *rqt4Δ::KanMX*, *ski2Δ::LEU2*	*rqt4Δ::KanMX* (8)	this study
23	*rqt4Δ::KanMX*, *new1Δ::HphMX*, *ski2Δ::LEU2*	*MATa*, *his3Δ*, *leu2Δ*, *met15Δ*, *ura3Δ*, *rqt4Δ::KanMX*, *new1Δ::HphMX*, *ski2Δ::LEU2*	*rqt4Δ::KanMX*, *new1Δ::HphMX* (9)	this study
24	*cue3Δ::KanMX*, *ski2Δ::LEU2*	*MATa*, *his3Δ*, *leu2Δ*, *met15Δ*, *ura3Δ*, *cue3Δ::KanMX*, *ski2Δ::LEU2*	*cue3Δ::KanMX* (10)	this study
25	*cue3Δ::KanMX*, *new1Δ::HphMX*, *ski2Δ::LEU2*	*MATa*, *his3Δ*, *leu2Δ*, *met15Δ*, *ura3Δ*, *cue3Δ::KanMX*, *new1Δ::HphMX*, *ski2Δ::LEU2*	*cue3Δ::KanMX*, *new1Δ::HphMX* (11)	this study
26	*cue2Δ::KanMX*, *ski2Δ::LEU2*	*MATa*, *his3Δ*, *leu2Δ*, *met15Δ*, *ura3Δ*, *cue2Δ::KanMX*, *ski2Δ::LEU2*	*cue2Δ::KanMX* (12)	this study
27	*new1Δ::KanMX*, *cue2Δ::HphMX*, *ski2Δ::LEU2*	*MATa*, *his3Δ*, *leu2Δ*, *met15Δ*, *ura3Δ*, *new1Δ::KanMX*, Cue2Δ::HphMX, *ski2Δ::LEU2*	*new1Δ::KanMX*, *cue2Δ::HphMX* (13)	this study
28	*dom34Δ::KanMX*, *ski2Δ::LEU2*	*MATa*, *his3Δ*, *leu2Δ*, *met15Δ*, *ura3Δ*, *dom34Δ::KanMX*, *ski2Δ::LEU2*	*dom34Δ::KanMX* (14)	this study

29	*new1Δ::HphMX*, *dom34Δ::KanMX*, *ski2Δ::LEU2*	*MATa*, *his3Δ*, *leu2Δ*, *met15Δ*, *ura3Δ*, *new1Δ::HphMX*, *dom34Δ::KanMX*, *ski2Δ::LEU2*	*new1Δ::HphMX*, *dom34Δ::KanMX* (15)	this study
30	*ski2Δ::LEU2*, *slh1Δ::HphMX*	*MATa*, *his3Δ*, *leu2Δ*, *met15Δ*, *ura3Δ*, *ski2Δ::LEU2*, *slh1Δ::HphMX*	*ski2Δ::LEU2* (16)	this study
31	*rqt4Δ::KanMX*, *ski2Δ::LEU2*, *cue3Δ::HIS3*	*MATa*, *his3Δ*, *leu2Δ*, *met15Δ*, *ura3Δ*, *rqt4Δ::KanMX*, *ski2Δ::LEU2*, *cue3Δ::HIS3*	*rqt4Δ::KanMX*, *ski2Δ::LEU2* (22)	this study
32	*new1Δ::KanMX*, *ski2Δ::LEU2*, *slh1Δ::HphMX*	*MATa*, *his3Δ*, *leu2Δ*, *met15Δ*, *ura3Δ*, *new1Δ::KanMX*, *ski2Δ::LEU2*, *slh1Δ::HphMX*	*new1Δ::KanMX*, *ski2Δ::LEU2* (17)	this study
33	*rqt4Δ::KanMX*, *new1Δ::HphMX*, *ski2Δ::LEU2*, *cue3Δ::HIS3*	*MATa*, *his3Δ*, *leu2Δ*, *met15Δ*, *ura3Δ*, *rqt4Δ::KanMX*, *new1Δ::HphMX*, *ski2Δ::LEU2*, *cue3Δ::HIS3*	*rqt4Δ::KanMX*, *new1Δ::HphMX*, *ski2Δ::LEU2* (23)	this study
34	BY4741, pGTSTR	*MATa*, *his3Δ*, *leu2Δ*, *met15Δ*, *ura3Δ*, BY4741, pGTSTR	BY4741 (1)	this study
35	*new1Δ::KanMX*, pGTSTR	*MATa*, *his3Δ*, *leu2Δ*, *met15Δ*, *ura3Δ*, *new1Δ::KanMX*, pGTSTR	*new1Δ::KanMX* (2)	this study
36	*new1Δ::HphMX*, pGTSTR	*MATa*, *his3Δ*, *leu2Δ*, *met15Δ*, *ura3Δ*, *new1Δ::HphMX*, pGTSTR	*new1Δ::HphMX* (3)	this study
37	*hel2Δ*, pGTSTR	*MATa*, *his3Δ*, *leu2Δ*, *met15Δ*, *ura3Δ*, *hel2Δ*, pGTSTR	*hel2Δ* (4)	this study
38	*hel2Δ*, *new1Δ::KanMX*, pGTSTR	*MATa*, *his3Δ*, *leu2Δ*, *met15Δ*, *ura3Δ*, *hel2Δ*, *new1Δ::KanMX*, pGTSTR	*hel2Δ*, *new1Δ::KanMX* (5)	this study
39	*slh1Δ::KanMX*, pGTSTR	*MATa*, *his3Δ*, *leu2Δ*, *met15Δ*, *ura3Δ*, *slh1Δ::KanMX*, pGTSTR	*slh1Δ::KanMX* (6)	this study
40	*slh1Δ::KanMX*, *new1Δ::HphMX*, pGTSTR	*MATa*, *his3Δ*, *leu2Δ*, *met15Δ*, *ura3Δ*, *slh1Δ::KanMX*, *new1Δ::HphMX*, pGTSTR	*slh1Δ::KanMX*, *new1Δ::HphMX* (7)	this study
41	*rqt4Δ::KanMX*, pGTSTR	*MATa*, *his3Δ*, *leu2Δ*, *met15Δ*, *ura3Δ*, *rqt4Δ::KanMX*, pGTSTR	*rqt4Δ::KanMX* (8)	this study
42	*rqt4Δ::KanMX*, *new1Δ::HphMX*, pGTSTR	*MATa*, *his3Δ*, *leu2Δ*, *met15Δ*, *ura3Δ*, *rqt4Δ::KanMX*, *new1Δ::HphMX*, pGTSTR	*rqt4Δ::KanMX*, *new1Δ::HphMX* (9)	this study
43	*cue3Δ::KanMX*, pGTSTR	*MATa*, *his3Δ*, *leu2Δ*, *met15Δ*, *ura3Δ*, *cue3Δ::KanMX*, pGTSTR	*cue3Δ::KanMX* (10)	this study
44	*cue3Δ::KanMX*, *new1Δ::HphMX*, pGTSTR	*MATa*, *his3Δ*, *leu2Δ*, *met15Δ*, *ura3Δ*, *cue3Δ::KanMX*, *new1Δ::HphMX*, pGTSTR	*cue3Δ::KanMX*, *new1Δ::HphMX* (11)	this study
45	*dom34Δ::KanMX*, pGTSTR	*MATa*, *his3Δ*, *leu2Δ*, *met15Δ*, *ura3Δ*, *dom34Δ::KanMX*, pGTSTR	*dom34Δ::KanMX* (14)	this study
46	*dom34Δ::KanMX*, *new1Δ::HphMX*, pGTSTR	*MATa*, *his3Δ*, *leu2Δ*, *met15Δ*, *ura3Δ*, *dom34Δ::KanMX*, *new1Δ::HphMX*, pGTSTR	*new1Δ::HphMX*, *dom34Δ::KanMX* (15)	this study
47	*cue2Δ::KanMX*, pGTSTR	*MATa*, *his3Δ*, *leu2Δ*, *met15Δ*, *ura3Δ*, *cue2Δ::KanMX*, pGTSTR	*cue2Δ::KanMX* (12)	this study
48	*new1Δ::KanMX*, *cue2Δ::HphMX*, pGTSTR	*MATa*, *his3Δ*, *leu2Δ*, *met15Δ*, *ura3Δ*, *new1Δ::KanMX*, *cue2Δ::HphMX*, pGTSTR	*new1Δ::KanMX*, *cue2Δ::HphMX* (13)	this study
49	*ski2Δ::LEU2*, pGTSTR	*MATa*, *his3Δ*, *leu2Δ*, *met15Δ*, *ura3Δ*, *ski2Δ::LEU2*, pGTSTR	*ski2Δ::LEU2* (16)	this study
50	*new1Δ::KanMX*, *ski2Δ::LEU2*, pGTSTR	*MATa*, *his3Δ*, *leu2Δ*, *met15Δ*, *ura3Δ*, *new1Δ::KanMX*, *ski2Δ::LEU2*, pGTSTR	*new1Δ::KanMX*, *ski2Δ::LEU2* (17)	this study
51	*new1Δ::HphMX*, *ski2Δ::LEU2*, pGTSTR	*MATa*, *his3Δ*, *leu2Δ*, *met15Δ*, *ura3Δ*, *new1Δ::HphMX*, *ski2Δ::LEU2*, pGTSTR	*new1Δ::HphMX*, *ski2Δ::LEU2* (18)	this study
52	*hel2Δ*, *ski2Δ::LEU2*, pGTSTR	*MATa*, *his3Δ*, *leu2Δ*, *met15Δ*, *ura3Δ*, *hel2Δ*, *ski2Δ::LEU2*, pGTSTR	*hel2Δ*, *ski2Δ::LEU2* (19)	this study
53	*hel2Δ*, *new1Δ::KanMX*, *ski2Δ::LEU2*, pGTSTR	*MATa*, *his3Δ*, *leu2Δ*, *met15Δ*, *ura3Δ*, *hel2Δ*, *new1Δ::KanMX*, *ski2Δ::LEU2*, pGTSTR	*hel2Δ*, *new1Δ::KanMX*, *ski2Δ::LEU2* (20)	this study
54	*slh1Δ::KanMX*, *ski2Δ::LEU2*, pGTSTR	*MATa*, *his3Δ*, *leu2Δ*, *met15Δ*, *ura3Δ*, *slh1Δ::KanMX*, *ski2Δ::LEU2*, pGTSTR	*slh1Δ::KanMX*, *ski2Δ::LEU2* (21)	this study
55	*rqt4Δ::KanMX*, *ski2Δ::LEU2*, pGTSTR	*MATa*, *his3Δ*, *leu2Δ*, *met15Δ*, *ura3Δ*, *rqt4Δ::KanMX*, *ski2Δ::LEU2*, pGTSTR	*rqt4Δ::KanMX*, *ski2Δ::LEU2* (22)	this study

56	*rqt4Δ::KanMX*, *new1Δ::HphMX*, *ski2Δ::LEU2*, pGTSTR	*MATa*, *his3Δ*, *leu2Δ*, *met15Δ*, *ura3Δ*, *rqt4Δ::KanMX*, *new1Δ::HphMX*, *ski2Δ::LEU2*, pGTSTR	*rqt4Δ::KanMX*, *new1Δ::HphMX*, *ski2Δ::LEU2* (23)	this study
57	*cue3Δ::KanMX*, *ski2Δ::LEU2*, pGTSTR	*MATa*, *his3Δ*, *leu2Δ*, *met15Δ*, *ura3Δ*, *cue3Δ::KanMX*, *ski2Δ::LEU2*, pGTSTR	*cue3Δ::KanMX*, *ski2Δ::LEU2* (24)	this study
58	*cue3Δ::KanMX*, *new1Δ::HphMX*, *ski2Δ::LEU2*, pGTSTR	*MATa*, *his3Δ*, *leu2Δ*, *met15Δ*, *ura3Δ*, *cue3Δ::KanMX*, *new1Δ::HphMX*, *ski2Δ::LEU2*, pGTSTR	*cue3Δ::KanMX*, *new1Δ::HphMX*, *ski2Δ::LEU2* (25)	this study
59	*dom34Δ::KanMX*, *ski2Δ::LEU2*, pGTSTR	*MATa*, *his3Δ*, *leu2Δ*, *met15Δ*, *ura3Δ*, *dom34Δ::KanMX*, *ski2Δ::LEU2*, pGTSTR	*dom34Δ::KanMX*, *ski2Δ::LEU2* (28)	this study
60	*new1Δ::HphMX*, *dom34Δ::KanMX*, *ski2Δ::LEU2*, pGTSTR	*MATa*, *his3Δ*, *leu2Δ*, *met15Δ*, *ura3Δ*, *new1Δ::HphMX*, *dom34Δ::KanMX*, *ski2Δ::LEU2*, pGTSTR	*new1Δ::HphMX*, *dom34Δ::KanMX*, *ski2Δ::LEU2* (29)	this study
61	*cue2Δ::KanMX*, *ski2Δ::LEU2*, pGTSTR	*MATa*, *his3Δ*, *leu2Δ*, *met15Δ*, *ura3Δ*, *cue2Δ::KanMX*, *ski2Δ::LEU2*, pGTSTR	*cue2Δ::KanMX*, *ski2Δ::LEU2* (26)	this study
62	*new1Δ::KanMX*, *cue2Δ::HphMX*, *ski2Δ::LEU2*, pGTSTR	*MATa*, *his3Δ*, *leu2Δ*, *met15Δ*, *ura3Δ*, *new1Δ::KanMX*, *cue2Δ::HphMX*, *ski2Δ::LEU2*, pGTSTR	*new1Δ::KanMX*, Cue2Δ::HphMX, *ski2Δ::LEU2* (27)	this study
63	BY4741, pGTRR	*MATa*, *his3Δ*, *leu2Δ*, *met15Δ*, *ura3Δ*, BY4741, pGTRR	BY4741 (1)	this study
64	*new1Δ::KanMX*, pGTRR	*MATa*, *his3Δ*, *leu2Δ*, *met15Δ*, *ura3Δ*, *new1Δ::KanMX*, pGTRR	*new1Δ::KanMX* (2)	this study
65	*new1Δ::HphMX*, pGTRR	*MATa*, *his3Δ*, *leu2Δ*, *met15Δ*, *ura3Δ*, *new1Δ::HphMX*, pGTRR	*new1Δ::HphMX* (3)	this study
66	*hel2Δ*, pGTRR	*MATa*, *his3Δ*, *leu2Δ*, *met15Δ*, *ura3Δ*, *hel2Δ*, pGTRR	*hel2Δ* (4)	this study
67	*hel2Δ*, *new1Δ::KanMX*, pGTRR	*MATa*, *his3Δ*, *leu2Δ*, *met15Δ*, *ura3Δ*, *hel2Δ*, *new1Δ::KanMX*, pGTRR	*hel2Δ*, *new1Δ::KanMX* (5)	this study
68	*slh1Δ::KanMX*, pGTRR	*MATa*, *his3Δ*, *leu2Δ*, *met15Δ*, *ura3Δ*, *slh1Δ::KanMX*, pGTRR	*slh1Δ::KanMX* (6)	this study
69	*slh1Δ::KanMX*, *new1Δ::HphMX*, pGTRR	*MATa*, *his3Δ*, *leu2Δ*, *met15Δ*, *ura3Δ*, *slh1Δ::KanMX*, *new1Δ::HphMX*, pGTRR	*slh1Δ::KanMX*, *new1Δ::HphMX* (7)	this study
70	*rqt4Δ::KanMX*, pGTRR	*MATa*, *his3Δ*, *leu2Δ*, *met15Δ*, *ura3Δ*, *rqt4Δ::KanMX*, pGTRR	*rqt4Δ::KanMX* (8)	this study
71	*rqt4Δ::KanMX*, *new1Δ::HphMX*, pGTRR	*MATa*, *his3Δ*, *leu2Δ*, *met15Δ*, *ura3Δ*, *rqt4Δ::KanMX*, *new1Δ::HphMX*, pGTRR	*rqt4Δ::KanMX*, *new1Δ::HphMX* (9)	this study
72	*cue3Δ::KanMX*, pGTRR	*MATa*, *his3Δ*, *leu2Δ*, *met15Δ*, *ura3Δ*, *cue3Δ::KanMX*, pGTRR	*cue3Δ::KanMX* (10)	this study
73	*cue3Δ::KanMX*, *new1Δ::HphMX*, pGTRR	*MATa*, *his3Δ*, *leu2Δ*, *met15Δ*, *ura3Δ*, *cue3Δ::KanMX*, *new1Δ::HphMX*, pGTRR	*cue3Δ::KanMX*, *new1Δ::HphMX* (11)	this study
74	*dom34Δ::KanMX*, pGTRR	*MATa*, *his3Δ*, *leu2Δ*, *met15Δ*, *ura3Δ*, *dom34Δ::KanMX*, pGTRR	*dom34Δ::KanMX* (14)	this study
75	*dom34Δ::KanMX*, *new1Δ::HphMX*, pGTRR	*MATa*, *his3Δ*, *leu2Δ*, *met15Δ*, *ura3Δ*, *dom34Δ::KanMX*, *new1Δ::HphMX*, pGTRR	*new1Δ::HphMX*, *dom34Δ::KanMX* (15)	this study
76	*cue2Δ::KanMX*, pGTRR	*MATa*, *his3Δ*, *leu2Δ*, *met15Δ*, *ura3Δ*, *cue2Δ::KanMX*, pGTRR	*cue2Δ::KanMX* (12)	this study
77	*new1Δ::KanMX*, *cue2Δ::HphMX*, pGTRR	*MATa*, *his3Δ*, *leu2Δ*, *met15Δ*, *ura3Δ*, *new1Δ::KanMX*, *cue2Δ::HphMX*, pGTRR	*new1Δ::KanMX*, *cue2Δ::HphMX* (13)	this study
78	*ski2Δ::LEU2*, pGTRR	*MATa*, *his3Δ*, *leu2Δ*, *met15Δ*, *ura3Δ*, *ski2Δ::LEU2*, pGTRR	*ski2Δ::LEU2* (16)	this study
79	*new1Δ::KanMX*, *ski2Δ::LEU2*, pGTRR	*MATa*, *his3Δ*, *leu2Δ*, *met15Δ*, *ura3Δ*, *new1Δ::KanMX*, *ski2Δ::LEU2*, pGTRR	*new1Δ::KanMX*, *ski2Δ::LEU2* (17)	this study
80	*new1Δ::HphMX*, *ski2Δ::LEU2*, pGTRR	*MATa*, *his3Δ*, *leu2Δ*, *met15Δ*, *ura3Δ*, *new1Δ::HphMX*, *ski2Δ::LEU2*, pGTRR	*new1Δ::HphMX*, *ski2Δ::LEU2* (18)	this study
81	*hel2Δ*, *ski2Δ::LEU2*, pGTRR	*MATa*, *his3Δ*, *leu2Δ*, *met15Δ*, *ura3Δ*, *hel2Δ*, *ski2Δ::LEU2*, pGTRR	*hel2Δ*, *ski2Δ::LEU2* (19)	this study
82	*hel2Δ*, *new1Δ::KanMX*, *ski2Δ::LEU2*, pGTRR	*MATa*, *his3Δ*, *leu2Δ*, *met15Δ*, *ura3Δ*, *hel2Δ*, *new1Δ::KanMX*, *ski2Δ::LEU2*, pGTRR	*hel2Δ*, *new1Δ::KanMX*, *ski2Δ::LEU2* (20)	this study
83	*slh1Δ::KanMX*, *ski2Δ::LEU2*, pGTRR	*MATa*, *his3Δ*, *leu2Δ*, *met15Δ*, *ura3Δ*, *slh1Δ::KanMX*, *ski2Δ::LEU2*, pGTRR	*slh1Δ::KanMX*, *ski2Δ::LEU2* (21)	this study

84	*rqt4Δ::KanMX*, *ski2Δ::LEU2*, pGTRR	*MATa*, *his3Δ*, *leu2Δ*, *met15Δ*, *ura3Δ*, *rqt4Δ::KanMX*, *ski2Δ::LEU2*, pGTRR	*rqt4Δ::KanMX*, *ski2Δ::LEU2* (22)	this study
85	*rqt4Δ::KanMX*, *new1Δ::HphMX*, *ski2Δ::LEU2*, pGTRR	*MATa*, *his3Δ*, *leu2Δ*, *met15Δ*, *ura3Δ*, *rqt4Δ::KanMX*, *new1Δ::HphMX*, *ski2Δ::LEU2*, pGTRR	*rqt4Δ::KanMX*, *new1Δ::HphMX*, *ski2Δ::LEU2* (23)	this study
86	*cue3Δ::KanMX*, *ski2Δ::LEU2*, pGTRR	*MATa*, *his3Δ*, *leu2Δ*, *met15Δ*, *ura3Δ*, *cue3Δ::KanMX*, *ski2Δ::LEU2*, pGTRR	*cue3Δ::KanMX*, *ski2Δ::LEU2* (24)	this study
87	*cue3Δ::KanMX*, *new1Δ::HphMX*, *ski2Δ::LEU2*, pGTRR	*MATa*, *his3Δ*, *leu2Δ*, *met15Δ*, *ura3Δ*, *cue3Δ::KanMX*, *new1Δ::HphMX*, *ski2Δ::LEU2*, pGTRR	*cue3Δ::KanMX*, *new1Δ::HphMX*, *ski2Δ::LEU2* (25)	this study
88	*dom34Δ::KanMX*, *ski2Δ::LEU2*, pGTRR	*MATa*, *his3Δ*, *leu2Δ*, *met15Δ*, *ura3Δ*, *dom34Δ::KanMX*, *ski2Δ::LEU2*, pGTRR	*dom34Δ::KanMX*, *ski2Δ::LEU2* (28)	this study
89	*new1Δ::HphMX*, *dom34Δ::KanMX*, *ski2Δ::LEU2*, pGTRR	*MATa*, *his3Δ*, *leu2Δ*, *met15Δ*, *ura3Δ*, *new1Δ::HphMX*, *dom34Δ::KanMX*, *ski2Δ::LEU2*, pGTRR	*new1Δ::HphMX*, *dom34Δ::KanMX*, *ski2Δ::LEU2* (29)	this study
90	*cue2Δ::KanMX*, *ski2Δ::LEU2*, pGTRR	*MATa*, *his3Δ*, *leu2Δ*, *met15Δ*, *ura3Δ*, *cue2Δ::KanMX*, *ski2Δ::LEU2*, pGTRR	*cue2Δ::KanMX*, *ski2Δ::LEU2* (26)	this study
91	*new1Δ::KanMX*, *cue2Δ::HphMX*, *ski2Δ::LEU2*, pGTRR	*MATa*, *his3Δ*, *leu2Δ*, *met15Δ*, *ura3Δ*, *new1Δ::KanMX*, *cue2Δ::HphMX*, *ski2Δ::LEU2*, pGTRR	*new1Δ::KanMX*, Cue2Δ::HphMX, *ski2Δ::LEU2* (27)	this study
92	*ski2Δ::LEU2*, *slh1Δ::HphMX*, pGTRR	*MATa*, *his3Δ*, *leu2Δ*, *met15Δ*, *ura3Δ*, *ski2Δ::LEU2*, *slh1Δ::HphMX*, pGTRR	*ski2Δ::LEU2*, *slh1Δ::HphMX* (30)	this study
93	*rqt4Δ::KanMX*, *ski2Δ::LEU2*, *cue3Δ::HIS3*, pGTRR	*MATa*, *his3Δ*, *leu2Δ*, *met15Δ*, *ura3Δ*, *rqt4Δ::KanMX*, *ski2Δ::LEU2*, *cue3Δ::HIS3*, pGTRR	*rqt4Δ::KanMX*, *ski2Δ::LEU2*, *cue3Δ::HIS3* (31)	this study
94	*new1Δ::KanMX*, *ski2Δ::LEU2*, *slh1Δ::HphMX*, pGTRR	*MATa*, *his3Δ*, *leu2Δ*, *met15Δ*, *ura3Δ*, *new1Δ::KanMX*, *ski2Δ::LEU2*, *slh1Δ::HphMX*, pGTRR	*new1Δ::KanMX*, *ski2Δ::LEU2*, *slh1Δ::HphMX* (32)	this study
95	*rqt4Δ::KanMX*, *new1Δ::HphMX*, *ski2Δ::LEU2*, *cue3Δ::HIS3*, pGTRR	*MATa*, *his3Δ*, *leu2Δ*, *met15Δ*, *ura3Δ*, *rqt4Δ::KanMX*, *new1Δ::HphMX*, *ski2Δ::LEU2*, *cue3Δ::HIS3*, pGTRR	*rqt4Δ::KanMX*, *new1Δ::HphMX*, *ski2Δ::LEU2*, *cue3Δ::HIS3* (33)	this study
96	*xrn1Δ::LEU2*	*MATa*, *his3Δ*, *leu2Δ*, *met15Δ*, *ura3Δ*, *xrn1Δ::LEU2*	BY4741 (1)	this study
97	*new1Δ::KanMX*, *xrn1Δ::LEU2*	*MATa*, *his3Δ*, *leu2Δ*, *met15Δ*, *ura3Δ*, *new1Δ::KanMX*, *xrn1Δ::LEU2*	*new1Δ::KanMX* (2)	this study
98	*hel2Δ*, *xrn1Δ::LEU2*	*MATa*, *his3Δ*, *leu2Δ*, *met15Δ*, *ura3Δ*, *hel2Δ*, *xrn1Δ::LEU2*	*hel2Δ* (4)	this study
99	*slh1Δ::KanMX*, *xrn1Δ::LEU2*	*MATa*, *his3Δ*, *leu2Δ*, *met15Δ*, *ura3Δ*, *slh1Δ::KanMX*, *xrn1Δ::LEU2*	*slh1Δ::KanMX* (6)	this study
100	*rqt4Δ::KanMX*, *xrn1Δ::LEU2*	*MATa*, *his3Δ*, *leu2Δ*, *met15Δ*, *ura3Δ*, *rqt4Δ::KanMX*, *xrn1Δ::LEU2*	*rqt4Δ::KanMX* (8)	this study
101	*dom34Δ::KanMX*, *xrn1Δ::LEU2*	*MATa*, *his3Δ*, *leu2Δ*, *met15Δ*, *ura3Δ*, *dom34Δ::KanMX*, *xrn1Δ::LEU2*	*dom34Δ::KanMX* (14)	this study
102	*cue2Δ::KanMX*, *xrn1Δ::LEU2*	*MATa*, *his3Δ*, *leu2Δ*, *met15Δ*, *ura3Δ*, *cue2Δ::KanMX*, *xrn1Δ::LEU2*	*cue2Δ::KanMX* (12)	this study
103	*hel2Δ*, *new1Δ::KanMX*, *xrn1Δ::LEU2*	*MATa*, *his3Δ*, *leu2Δ*, *met15Δ*, *ura3Δ*, *hel2Δ*, *new1Δ::KanMX*, *xrn1Δ::LEU2*	*hel2Δ*, *new1Δ::KanMX* (5)	this study
104	*slh1Δ::KanMX*, *new1Δ::HphMX*, *xrn1Δ::LEU2*	*MATa*, *his3Δ*, *leu2Δ*, *met15Δ*, *ura3Δ*, *slh1Δ::KanMX*, *new1Δ::HphMX*, *xrn1Δ::LEU2*	*slh1Δ::KanMX*, *new1Δ::HphMX* (7)	this study
105	*rqt4Δ::KanMX*, *new1Δ::HphMX*, *xrn1Δ::LEU2*	*MATa*, *his3Δ*, *leu2Δ*, *met15Δ*, *ura3Δ*, *rqt4Δ::KanMX*, *new1Δ::HphMX*, *xrn1Δ::LEU2*	*rqt4Δ::KanMX*, *new1Δ::HphMX* (9)	this study
106	*new1Δ::HphMX*, *dom34Δ::KanMX*, *xrn1Δ::LEU2*	*MATa*, *his3Δ*, *leu2Δ*, *met15Δ*, *ura3Δ*, *new1Δ::HphMX*, *dom34Δ::KanMX*, *xrn1Δ::LEU2*	*new1Δ::HphMX*, *dom34Δ::KanMX* (15)	this study
107	*new1Δ::KanMX*, *cue2Δ::HphMX*, *xrn1Δ::LEU2*	*MATa*, *his3Δ*, *leu2Δ*, *met15Δ*, *ura3Δ*, *new1Δ::KanMX*, *cue2Δ::HphMX*, *xrn1Δ::LEU2*	*new1Δ::KanMX*, *cue2Δ::HphMX* (13)	this study
108	*xrn1Δ::LEU2*, *slh1Δ::HphMX*	*MATa*, *his3Δ*, *leu2Δ*, *met15Δ*, *ura3Δ*, *xrn1Δ::LEU2*, *slh1Δ::HphMX*	*xrn1Δ::LEU2* (96)	this study
109	*hel2Δ*, *xrn1Δ::LEU2*, *slh1Δ::HphMX*	*MATa*, *his3Δ*, *leu2Δ*, *met15Δ*, *ura3Δ*, *hel2Δ*, *xrn1Δ::LEU2*, *slh1Δ::HphMX*	*hel2Δ*, *xrn1Δ::LEU2* (98)	this study

110	*new1Δ::KanMX*, *xrn1Δ::LEU2*, *slh1Δ::HphMX*	*MATa*, *his3Δ*, *leu2Δ*, *met15Δ*, *ura3Δ*, *new1Δ::KanMX*, *xrn1Δ::LEU2*, *slh1Δ::HphMX*	*new1Δ::KanMX*, *xrn1Δ::LEU2* (97)	this study
111	*hel2Δ*, *new1Δ::KanMX*, *xrn1Δ::LEU2*, *slh1Δ::HphMX*	*MATa*, *his3Δ*, *leu2Δ*, *met15Δ*, *ura3Δ*, *hel2Δ*, *new1Δ::KanMX*, *xrn1Δ::LEU2*, *slh1Δ::HphMX*	*hel2Δ*, *new1Δ::KanMX*, *xrn1Δ::LEU2* (103)	this study
112	*xrn1Δ::LEU2*, pGTRR	*MATa*, *his3Δ*, *leu2Δ*, *met15Δ*, *ura3Δ*, *xrn1Δ::LEU2*, pGTRR	*xrn1Δ::LEU2* (96)	this study
113	*new1Δ::KanMX*, *xrn1Δ::LEU2*, pGTRR	*MATa*, *his3Δ*, *leu2Δ*, *met15Δ*, *ura3Δ*, *new1Δ::KanMX*, *xrn1Δ::LEU2*, pGTRR	*new1Δ::KanMX*, *xrn1Δ::LEU2* (97)	this study
114	*hel2Δ*, *xrn1Δ::LEU2*, pGTRR	*MATa*, *his3Δ*, *leu2Δ*, *met15Δ*, *ura3Δ*, *hel2Δ*, *xrn1Δ::LEU2*, pGTRR	*hel2Δ*, *xrn1Δ::LEU2* (98)	this study
115	*slh1Δ::KanMX*, *xrn1Δ::LEU2*, pGTRR	*MATa*, *his3Δ*, *leu2Δ*, *met15Δ*, *ura3Δ*, *slh1Δ::KanMX*, *xrn1Δ::LEU2*, pGTRR	*slh1Δ::KanMX*, *xrn1Δ::LEU2* (99)	this study
116	*rqt4Δ::KanMX*, *xrn1Δ::LEU2*, pGTRR	*MATa*, *his3Δ*, *leu2Δ*, *met15Δ*, *ura3Δ*, *rqt4Δ::KanMX*, *xrn1Δ::LEU2*, pGTRR	*rqt4Δ::KanMX*, *xrn1Δ::LEU2* (100)	this study
117	*dom34Δ::KanMX*, *xrn1Δ::LEU2*, pGTRR	*MATa*, *his3Δ*, *leu2Δ*, *met15Δ*, *ura3Δ*, *dom34Δ::KanMX*, *xrn1Δ::LEU2*, pGTRR	*dom34Δ::KanMX*, *xrn1Δ::LEU2* (101)	this study
118	*cue2Δ::KanMX*, *xrn1Δ::LEU2*, pGTRR	*MATa*, *his3Δ*, *leu2Δ*, *met15Δ*, *ura3Δ*, *cue2Δ::KanMX*, *xrn1Δ::LEU2*, pGTRR	*cue2Δ::KanMX*, *xrn1Δ::LEU2* (102)	this study
119	*hel2Δ*, *new1Δ::KanMX*, *xrn1Δ::LEU2*, pGTRR	*MATa*, *his3Δ*, *leu2Δ*, *met15Δ*, *ura3Δ*, *hel2Δ*, *new1Δ::KanMX*, *xrn1Δ::LEU2*, pGTRR	*hel2Δ*, *new1Δ::KanMX*, *xrn1Δ::LEU2* (103)	this study
120	*slh1Δ::KanMX*, *new1Δ::HphMX*, *xrn1Δ::LEU2*, pGTRR	*MATa*, *his3Δ*, *leu2Δ*, *met15Δ*, *ura3Δ*, *slh1Δ::KanMX*, *new1Δ::HphMX*, *xrn1Δ::LEU2*, pGTRR	*slh1Δ::KanMX*, *new1Δ::HphMX*, *xrn1Δ::LEU2* (104)	this study
121	*rqt4Δ::KanMX*, *new1Δ::HphMX*, *xrn1Δ::LEU2*, pGTRR	*MATa*, *his3Δ*, *leu2Δ*, *met15Δ*, *ura3Δ*, *rqt4Δ::KanMX*, *new1Δ::HphMX*, *xrn1Δ::LEU2*, pGTRR	*rqt4Δ::KanMX*, *new1Δ::HphMX*, *xrn1Δ::LEU2* (105)	this study
122	*new1Δ::HphMX*, *dom34Δ::KanMX*, *xrn1Δ::LEU2*, pGTRR	*MATa*, *his3Δ*, *leu2Δ*, *met15Δ*, *ura3Δ*, *new1Δ::HphMX*, *dom34Δ::KanMX*, *xrn1Δ::LEU2*, pGTRR	*new1Δ::HphMX*, *dom34Δ::KanMX*, *xrn1Δ::LEU2* (106)	this study
123	*new1Δ::KanMX*, *cue2Δ::HphMX*, *xrn1Δ::LEU2*, pGTRR	*MATa*, *his3Δ*, *leu2Δ*, *met15Δ*, *ura3Δ*, *new1Δ::KanMX*, *cue2Δ::HphMX*, *xrn1Δ::LEU2*, pGTRR	*new1Δ::KanMX*, *cue2Δ::HphMX*, *xrn1Δ::LEU2* (107)	this study
124	*xrn1Δ::LEU2*, *slh1Δ::HphMX*, pGTRR	*MATa*, *his3Δ*, *leu2Δ*, *met15Δ*, *ura3Δ*, *xrn1Δ::LEU2*, *slh1Δ::HphMX*, pGTRR	*xrn1Δ::LEU2*, *slh1Δ::HphMX* (108)	this study
125	*hel2Δ*, *xrn1Δ::LEU2*, *slh1Δ::HphMX*, pGTRR	*MATa*, *his3Δ*, *leu2Δ*, *met15Δ*, *ura3Δ*, *hel2Δ*, *xrn1Δ::LEU2*, *slh1Δ::HphMX*, pGTRR	*hel2Δ*, *xrn1Δ::LEU2*, *slh1Δ::HphMX* (109)	this study
126	*new1Δ::KanMX*, *xrn1Δ::LEU2*, *slh1Δ::HphMX*, pGTRR	*MATa*, *his3Δ*, *leu2Δ*, *met15Δ*, *ura3Δ*, *new1Δ::KanMX*, *xrn1Δ::LEU2*, *slh1Δ::HphMX*, pGTRR	*new1Δ::KanMX*, *xrn1Δ::LEU2*, *slh1Δ::HphMX* (110)	this study
127	*hel2Δ*, *new1Δ::KanMX*, *xrn1Δ::LEU2*, *slh1Δ::HphMX*, pGTRR	*MATa*, *his3Δ*, *leu2Δ*, *met15Δ*, *ura3Δ*, *hel2Δ*, *new1Δ::KanMX*, *xrn1Δ::LEU2*, *slh1Δ::HphMX*, pGTRR	*hel2Δ*, *new1Δ::KanMX*, *xrn1Δ::LEU2*, *slh1Δ::HphMX* (111)	this study
128	BY4741, pEV-*HIS3*	*MATa*, *his3Δ*, *leu2Δ*, *met15Δ*, *ura3Δ*, BY4741, pEV-*HIS3*	BY4741 (1)	this study
129	BY4741, pNew1-FLAG-*HIS3*	*MATa*, *his3Δ*, *leu2Δ*, *met15Δ*, *ura3Δ*, BY4741, pNew1-FLAG-*HIS3*	BY4741 (1)	this study
130	*new1Δ::KanMX*, pEV-*HIS3*	*MATa*, *his3Δ*, *leu2Δ*, *met15Δ*, *ura3Δ*, *new1Δ::KanMX*, pEV-*HIS3*	*new1Δ::KanMX* (2)	this study
131	*new1Δ::KanMX*, pNew1-FLAG-*HIS3*	*MATa*, *his3Δ*, *leu2Δ*, *met15Δ*, *ura3Δ*, *new1Δ::KanMX*, pNew1-FLAG-*HIS3*	*new1Δ::KanMX* (2)	this study
132	*ski2Δ::LEU2*, pEV-*HIS3*	*MATa*, *his3Δ*, *leu2Δ*, *met15Δ*, *ura3Δ*, *ski2Δ::LEU2*, pEV-*HIS3*	*ski2Δ::LEU2* (16)	this study
133	*ski2Δ::LEU2*, pNew1-FLAG-*HIS3*	*MATa*, *his3Δ*, *leu2Δ*, *met15Δ*, *ura3Δ*, *ski2Δ::LEU2*, pNew1-FLAG-*HIS3*	*ski2Δ::LEU2* (16)	this study
134	*new1Δ::KanMX*, *ski2Δ::LEU2*, pEV-*HIS3*	*MATa*, *his3Δ*, *leu2Δ*, *met15Δ*, *ura3Δ*, *new1Δ::KanMX*, *ski2Δ::LEU2*, pEV-*HIS3*	*new1Δ::KanMX* (2)	this study
135	*new1Δ::KanMX*, *ski2Δ::LEU2*, pNew1-FLAG-*HIS3*	*MATa*, *his3Δ*, *leu2Δ*, *met15Δ*, *ura3Δ*, *new1Δ::KanMX*, *ski2Δ::LEU2*, pNew1-FLAG-*HIS3*	*new1Δ::KanMX* (2)	this study

136	New1-HTP	*MATa*, *his3Δ*, *leu2Δ*, *met15Δ*, *ura3Δ*, New1-HTP	BY4741 (1)	this study
137	*hel2Δ*, New1-HTP	*MATa*, *his3Δ*, *leu2Δ*, *met15Δ*, *ura3Δ*, *hel2Δ*, New1-HTP	*hel2Δ* (4)	this study
138	Hel2-HTP	*MATa*, *his3Δ*, *leu2Δ*, *met15Δ*, *ura3Δ*, Hel2-HTP	BY4741 (1)	Winz et al., 2019
139	Hel2-HTP, *new1Δ::KanMX*	*MATa*, *his3Δ*, *leu2Δ*, *met15Δ*, *ura3Δ*, Hel2-HTP, *new1Δ::KanMX*	Hel2-HTP (138)	this study
140	New1-FTP	*MATa*, *his3Δ*, *leu2Δ*, *met15Δ*, *ura3Δ*, New1-FTP	BY4741 (1)	this study

Supplementary Table S2. List of oligonucleotides used in this study.

Name	Sequence	Usage
	**Strain construction**	
NEW1-KanMX-HphMX-F	5'-GTAAATACAACGACAATCAGTGCTAATTCAACT CAGGATGCGGATCCCCGGGTTAATTAA-OH-3'	deletion of *NEW1* by exchange with *KanMX* or *HphMX* cassette
NEW1-KanMX-R	5'-CGAAGTTAGCGAAGATAAAACACTAGCCAGTA GGCTTTCAGAATTCGAGCTCGTTTAAAC-OH-3'	deletion of *NEW1* by exchange with *KanMX* cassette
NEW1-in-F	5'-GCGTCGATCTATAGTGC-OH-3'	testing deletion of *NEW1*
NEW1-in-R	5'-GAGCGTCATATGCTTGTC-OH-3'	testing deletion or tagging of *NEW1*
NEW1-KanMX-HphMX-F	5'-GTAAATACAACGACATCAGTGCTAATTCAACTCA GGATGCGTACGCTGCAGGTCGAC-OH-3'	deletion of *NEW1* by exchange with *KanMX* or *HphMX* cassette
NEW1-KanMX-HphMX-R	5'-CGAAGTTAGCGAAGATAAAACACTAGCCAGTA GGCTTTCAATCGATGAATTCGAGCTCG-OH-3'	deletion of *NEW1* by exchange with *KanMX* or *HphMX* cassette
KanMX-in-F	5'-TTAAGTGCGCAGAAAGTAAT-OH-3'	testing presence of *KanMX* cassette
HphMX-in-R	5'-ACATGGGGATGTATGGGC-OH-3'	testing presence of *KanMX* or *HphMX* cassette
SLH1-KanMX-F	5'-TGAGAAGTAGATCCGTACCATCAATAGCCGGC TCAAGATGCGGATCCCCGGGTTAATTAA-OH-3'	deletion of *SLH1* by exchange with *KanMX* cassette
SLH1-KanMX-R	5'-TCTTTCACAAAATAATTGTTGTTTTAATTGTGTCT CACTAGAATTCGAGCTCGTTTAAAC-OH-3'	deletion of *SLH1* by exchange with *KanMX* cassette
SLH1-in-F	5'-TGAGAAGTAGATCCGTACC-OH-3'	testing deletion of *SLH1*
SLH1-in-R	5'-ATCGGATTAACATTATCACCTG-OH-3'	testing deletion of *SLH1*
RQT4-KanMX-F	5'-GCTTAATTATTTAACCTTGGGTTTGCAGTAATTA ATAATGCGTACGCTGCAGGTCGAC-OH-3'	deletion of *RQT4* by exchange with *KanMX* cassette
RQT4-KanMX-R	5'-ATCCTCTATCATATATAATATAAACTTTTACCATT CATCAATCGATGAATTCGAGCTCG-OH-3'	deletion of *RQT4* by exchange with *KanMX* cassette
RQT4-in-F	5'-GGCAAAGTGAACGTAGTC-OH-3'	testing deletion of *RQT4*
RQT4-in-R	5'-AAGCCAACTAAGGAAAACTC-OH-3'	testing deletion of *RQT4*
CUE3-KanMX-F	5'-TCTTGGAAATTTTTATAGGATAGAATCACTA AGAATAATGCGGATCCCCGGGTTAATTAA-OH-3'	deletion of *CUE3* by exchange with *KanMX* cassette
CUE3-KanMX-R	5'-ACATACGCTTGTCTATCTTGTATGCTGATGCA TTTTATCAGAATTCGAGCTCGTTTAAAC-OH-3'	deletion of *CUE3* by exchange with *KanMX* cassette
CUE3-in-F	5'-CACTAGTTCTCATAAATGAGAGAC-OH-3'	testing deletion of *CUE3*
CUE3-in-R	5'-CGCTTGTCTATCTTGTATGC-OH-3'	testing deletion of *CUE3*
CUE2-KanMX-HphMX-F	5'-AATTATGACACCTCATTTATCGTGCATATAAGATC ATGCATAGCTTGCCTCGTCCCC-OH-3'	deletion of *CUE2* by exchange with *KanMX* or *HphMX* cassette
CUE2-KanMX-HphMX-R	5'-GCCTAGCGTTTATAACCTTTGTAGAAAAATCAGA TAGGTTCGCATAGGCCACTAGTGG-OH-3'	deletion of *CUE2* by exchange with *KanMX* or *HphMX* cassette
CUE2-in-F	5'-GACACTCACAGACAAGCCTCAGGGG-OH-3'	testing deletion of *CUE2*
DOM34-KanMX-F	5'-TTTTGTTCAATTATCGCATTCCTATCATAGCAAAA ATATGCGGATCCCCGGGTTAATTAA-OH-3'	deletion of *DOM34* by exchange with *KanMX* cassette
DOM34-KanMX-R	5'-TTTTATGTGTACATTACTTTTTTCTTACATAGTAA ATCTAGAATTCGAGCTCGTTTAAAC-OH-3'	deletion of *DOM34* by exchange with *KanMX* cassette
DOM34-in-F	5'-GCGTGATGAAAGTGTACATA-OH-3'	testing deletion of *DOM34*
SKI2-LEU2-F	5'-AACCTAACTCACAAAATTTACTGTACTAATACTA ATTTATGCGTATCACGAGGCCC-OH-3'	deletion of *SKI2* by exchange with *LEU2* cassette
SKI2-LEU2-R	5'-CTTTTATAAACATGACTCACATTGAGAATAAATG AGCTCTGATAAGCTGTCAAACATGAG-OH-3'	deletion of *SKI2* by exchange with *LEU2* cassette
SKI2-in-F	5'-GTTAATGATATCACGACGGAC-OH-3'	testing deletion of *SKI2*
LEU2-in-R	5'-GAAAAAGGTATATGCGTCAGG-OH-3'	testing presence of *LEU2* cassette
NEW1-HTP-F	5'-AAGGTACACCAAAACCAGTTGATACTGACGATG AAGAAGATGAGCACCATCACCATCACC-OH-3'	tagging of New1
NEW1-HTP-FTP-R	5'-AACAAACGAAGTTAGCGAAGATAAAACACTAG CCAGTAGGCTTTACGACTCACTATAGGG-OH-3'	tagging of New1
NEW1-FTP-F	5'-AAGGTACACCAAAACCAGTTGATACTGACGAT	tagging of New1

	GAAGAAGATGAGGACTACAAAGACGATG-OH-3'	
HTP-in-F	5'-TATGATTGTCTCCGGG-OH-3'	validated HTP tagging
XRN1-LEU2-F	5'-ACTTGTAACAACAGCAGCAACAAATATATATCAGTACGGTGCG TATCACGAGGCCC-OH-3'	deletion of *XRN1* by exchange with *LEU2* cassette
XRN1-LEU2-R	5'-TAAAGTAACCTCGAATATACTTCGTTTTTAGTCGTATGTTCTACC CTATGAACATATTCC-OH-3'	deletion of *XRN1* by exchange with *LEU2* cassette
XRN1-in-F	5'-TTTCTAAAGGATACTGTCTTCTTCC-OH-3'	testing deletion of *XRN1*
	**Northern Blot**	
SCR1-NP	5'-ATCCCGGCCGCCTCCATCAC-OH-3'	Northern blot probe for *SCR1*
PGK1-NP	5'-TAAGATGGCCAAGAATGGTCTGGTTGGG-OH-3'	Northern blot probe for *PGK1*
SNR190-NP	5'-TCGTCATGGTCGAATCGGACGAGG-OH-3'	Northern blot probe for *SNR190*
FLAG-NP	5'-ACTTGTCGTCATCGTCTTTGTAGTCC-OH-3'	Northern blot probe for *FLAG*
GPM1-NP	5'-AGCGTCGATTGGGGGAGGTGGAAC-OH-3'	Northern blot probe for *GPM1*
ADH1-NP	5'-ACCGATCTTCCAGCCCTTAAC-OH-3'	Northern blot probe for *ADH1*
TDH3-NP	5'-ACCTTCTTGGCACCAGCGTC-OH-3'	Northern blot probe for *TDH3*
	**Cloning**	
New1-Xba1-F	5'-TCGACGGATTCTAGAATGCCTCCAAAGAAGTTTAAGG-OH-3'	Amplyfing *NEW1* CDS with Xba1 recogntion overhang
New1-Xba1-R	5'-CAGGTGTCTAGAACTAGTGGTCAATCCTTGTCGT CATCGTCTTTGTAGTC-OH-3'	Amplyfing *NEW1* CDS with Xba1 recogntion overhang
Backbone-SangerSeq-1	5'-ACTCGCCATTTCAAAGAATACG-OH-3'	Sanger-Sequecning of pNew1-FLAG-*HIS3* and pEV-*HIS3*
New1-SangerSeq-1	5'-TCGTGAGGAAATTGCAGCCG-OH-3'	Sanger-Sequecning of pNew1-FLAG-*HIS3*
New1-SangerSeq-2	5'-CATTCTTCAATGTCGCACTTG-OH-3'	Sanger-Sequecning of pNew1-FLAG-*HIS3*
New1-SangerSeq-3	5'-CTCAGGCAGACTGACTTTAGAAG-OH-3'	Sanger-Sequecning of pNew1-FLAG-*HIS3*
New1-SangerSeq-4	5'-GACGGCAAACCAATATTTGCAATG-OH-3'	Sanger-Sequecning of pNew1-FLAG-*HIS3*
Mut-pBS1539-His2FLAG-F	5'-P-GACTACAAAGACGATGACGACAAGGATTATGAT ATTCCAACTACTG-OH-3'	site-directed mutagenesis of HTP tag of pBS15139 to FTP tag, including FLAG sequence
Mut-pBS1539-R	5'-P-CTCCATGGATCCTCCAG-OH-3'	site-directed mutagenesis of HTP tag of pBS15139 to FTP tag
pBS1539-FTP-Sanger-Seq	5'-CGCCAAGCGCGCAATTAACCC-OH-3'	Sanger-Sequencing of mutated FTP-tag in pBS15139-FTP
	**CRAC**	
L3	5'-rAppTGGAATTCTCGGGTGCCAAGG-ddC-3'	3'-linker
L5Ac	5'-invddT-ACACrGrArCrGrCrUrCrUrUrCrCrGrArUrCrU rNrNrNrGrCrGrCrArGrC-OH-3'	5'-linker, barcode underlined
L5Ad	5'-invddT-ACACrGrArCrGrCrUrCrUrUrCrCrGrArUrCrU rNrNrNrCrGrCrUrUrArGrC-OH-3'	5'-linker, barcode underlined
L5Bc	5'-invddT-ACACrGrArCrGrCrUrCrUrUrCrCrGrArUrCrU rNrNrNrCrArCrUrArGrC-OH-3'	5'-linker, barcode underlined
L5Bd	5'-invddT-ACACrGrArCrGrCrUrCrUrUrCrCrGrArUrCrU rNrNrNrUrCrUrCrUrArGrC-OH-3'	5'-linker, barcode underlined
L5Ca	5'-invddT-ACACrGrArCrGrCrUrCrUrUrCrCrGrArUrCrU rNrNrNrCrUrArGrC-OH-3'	5'-linker, barcode underlined
L5Da	5'-invddT-ACACrGrArCrGrCrUrCrUrUrCrCrGrArUrCrU rNrNrNrCrGrUrGrArUrN-OH-3'	5'-linker, barcode underlined
L5Db	5'-invddT-ACACrGrArCrGrCrUrCrUrUrCrCrGrArUrCrU rNrNrNrGrCrArCrUrArN-OH-3'	5'-linker, barcode underlined
L5Dc	5'-invddT-ACACrGrArCrGrCrUrCrUrUrCrCrGrArUrCrU rNrNrNrUrArGrUrGrCrN-OH-3'	5'-linker, barcode underlined
L5De	5'-invddT-ACACrGrArCrGrCrUrCrUrUrCrCrGrArUrCrU rNrNrNrArUrCrArCrGrN-OH-3'	5'-linker, barcode underlined

L5Ea	5'-invddT-ACACrGrArCrGrCrUrCrUrUrCrCrGrArUrCrU rNrNrNrCrArCrUrGrUrN-OH-3'	5'-linker, barcode underlined
L5Eb	5'-invddT-ACACrGrArCrGrCrUrCrUrUrCrCrGrArUrCrU rNrNrNrGrUrGrArCrArN-OH-3'	5'-linker, barcode underlined
L5Ec	5'-invddT-ACACrGrArCrGrCrUrCrUrUrCrCrGrArUrCrU rNrNrNrUrGrUrCrArCrN-OH-3'	5'-linker, barcode underlined
L5Ed	5'-invddT-ACACrGrArCrGrCrUrCrUrUrCrCrGrArUrCrU rNrNrNrArCrArGrUrGrN-OH-3'	5'-linker, barcode underlined
PRT_D01a_RT	5'-CAAGCAGAAGACGGCATACGAGATCCACGCTT CATTCCTGGCCTTGGCACCCGAGAATTCCA-OH-3'	RT, indexing in same step, index underlined
PRT_D02a _RT	5'-CAAGCAGAAGACGGCATACGAGATGATGCTAC CATTCCTGGCCTTGGCACCCGAGAATTCCA-OH-3'	RT, indexing in same step, index underlined
PRT	5’-CAGACGTGTGCTCTTCCGATCT-OH-3’	RT without indexing
PPCR_r _1	5'-CAAGCAGAAGACGGCATACGA-OH-3'	library PCR, reverse primer for indexed cDNA (30°C samples)
PPCR_r_2	5'-CAAGCAGAAGACGGCATACGAGATCGGTCTCG GCATTCCTGGCCTTGGCACCCGAGAATTCC-OH-3'	library PCR, reverse primer for non-indexed cDNA (20°C samples)
PPCR_f	5'-AATGATACGGCGACCACCGAGATCTACACTC TTTCCCTACACGACGCTCTTCCGATCT-OH-3‘	library PCR
Pi_index_read	5'-GGAATTCTCGGGTGCCAAGGCCAGGAATG-OH-3'	custom index read primer (30°C samples)
	**Long-read sequencing**	
	Native adapter(NA) (ONT, SQK-NBD114.24)	

Supplementary Table S3. List of plasmids used in this study.

Plasmid	Reference
pKK148	(10)
pBS1539-FTP	this study
pBS1539-HTP	(11)
pEV-*HIS3*	This study
pFA6-kanMX	(12)
pGTRR	(13)
pGTSTR	(13)
pNew1-FLAG-*HIS3*	This study
pyM18	(14)
pyM20	(14)
YEp181-CUP1-His-Ubi	(15)

Supplementary Table S4. List of materials used in this study.

Product	Company	Product number	Comment
[gamma-P32] ATP	Hartmann Analytics	SRP-501	
2-Mercaptoethanol	Roth	4227.3	
2-Propanol ≥99,7%	VWR	20842.33	
Accessories Beads Zirconia/glass beads, 0,5 mm	Roth	N034.1	
Acetic acid glacial ≥99.7%	VWR	20104.334	
Agar	Formedium	AGA03	
Albumin, Acetylated from bovine serum	Sigma-Aldrich	B8894	
Ammonium peroxydisulphate	Roth	9592.3	
Ampicillin Sodium	Formedium	AMP05	100 µg/mL
AMPure XP beads-based	AGENCOURT®	A63881	
anti-alpha Tubulin antibody	Abcam	ab184970	1:10,000
anti-Mouse (GAM)-HRP conjugate antibody	Bio-Rad Laboratories	1705047	1:20,000
anti-PGK1 antibody	Thermo Scientific™	22C5D8	1:10,000
ATP Solution (100 mM)	Thermo Scientific™	R0441	
Blunt/TA Ligase Master Mix	New England Biolabs	M0367	
Bromophenol blue sodium salt	Roth	A512.2	
Complete supplement mixture	Formedium	DCS0019	
Complete supplement mixture drop out -URA	Formedium	DCS0169	
cOmplete™, Mini, EDTA-free Protease Inhibitor Cocktail	Roche	04693159001	
D(+)-Glucose	Roth	HN06.3	
Deoxycholic acid, sodium salt	Sigma-Aldrich	D6750-10G	
di-Sodium hydrogen phosphate	Roth	P030.1	
DreamTaq Green DNA Polymerase	Thermo Scientific™	EP0712	
Dynabeads™ M-280 Tosylactivated	Invitrogen™	14203	
E. coli Poly(A) Polymerase	New England Biolabs	M0276	
ECL anti Rabbit IgG HRP	Cytiva	NA934W	1:5,000
Ethanol absolute, >= 99%	Fisher Chemical	E/0600DF/F21	
Ethidium bromide solution 1 %	Roth	2218.2	
Ethylendiamine tetraacetic acid disodium salt dihydrate	Roth	8043.2	
Exonuclease I	Thermo Scientific™	EN0582	
FastAP Thermosensitive Alkaline Phosphatase	Thermo Scientific™	EF0651	
G-418 DiSulphate	Formedium	G4181	200 µg/mL
GeneJET Gel Extraction Kit	Thermo Scientific™	K0691	
GeneJET Plasmid Miniprep Kit	Thermo Scientific™	K0503	
Glycerol 99%	Grüssing	110521000	
Glycine	Roth	79.4	
Guanidine hydrochloride	Roth	0037.1	
Halo TEV protease	Promega	G660B	
HEPES	Formedium	HEPES01	
Hybond-N	GE Healthcare	RPN303N	
Hybond-N+	GE Healthcare	RPN203B	

Hygromycin B solution	Roth	1287.2	300 µg/mL
IgG from rabbit serum	Sigma-Aldrich	I5006	
IgG Sepharose™ 6 Fast Flow	Cytiva	17-0969-01	
Imidazole	Roth	X998.3	
La Taq	Takara	RR002	
L-Histidin	Roth	1696.1	
Lithium acetate	Roth	5447.2	
L-Leucin	Roth	1699.1	
L-Tryptophan	Roth	1739.1	
Maxima H Minus Double-Stranded cDNA Synthesis Kit	Thermo Scientific™	K2561	
MetaPhor Agarose	BioZym	859181	
MinElute Gel Extraction Kit	Qiagen	28604	
MOPS	Roth	6979.2	
Mouse IgG (Magnetic Bead Conjugate)	Cell Signaling	5873	
Native Barcoding Kit 24 V14	Oxford Nanopore Technologies	SQK-NBD114.24	
NEBNext® Quick Ligation Module	New England Biolabs	E6056	
NEBNext® Ultra™ II End Repair/dA-Tailing Module	New England Biolabs	E7546	
Ni-NTA sepharose beads	Qiagen	30210	
NorthernMax™-Gly Sample Loading Dye	Invitrogen™	AM8551	
Novex™ TBE Gels, 6%	Invitrogen™	EC6265	
NP-40-Alternative	Sigma-Aldrich	492016-100ML	
NuPAGE 4-12% Bis–Tris gels	Invitrogen™	NP0335	
PageRuler™ Plus Prestained Protein Ladder, 10 to 250 kDa	Thermo Scientific™	26619	
PEG	Sigma-Aldrich	88276-250G-F	
Peroxidase Anti-Peroxidase (PAP) Soluble Complex antibody produced in rabbit	Sigma-Aldrich	P1291	1:2,000
Phosphate Buffered Saline	Formedium	PBS100L	
Phusion™ High-Fidelity DNA-Polymerase	Thermo Scientific™	F530L	
Pierce™ ECL Western Blotting-Substrat	Thermo Scientific™	32106	
Ponceau S (C.I. 27195)	Roth	5938.2	
Powdered milk	Roth	T145.3	
Proteinase K	Roth	7528.1	
PureCube Ni-NTA MagBeads	Cubebiotech	31201	
QIAquick Gel Extraction Kit	Qiagen	28704	
Qubit™ dsDNA Quantification Assay Kit	Invitrogen™	Q3285	
RiboRuler Low Range RNA Ladder	Thermo Scientific™	SM1831	
RNace-IT Ribonuclease Cocktail	Agilent Technologies	400720	
RNAClean XP beads	AGENCOURT®	A63987	
RNase A/T1 Mix	Thermo Scientific™	EN0551	
RNasin® Ribonuclease Inhibitor	Promega	N2515	
ROTI®Aqua-Phenol	Roth	A980.3	
ROTI®C/I	Roth	X984.2	
ROTI®Quant universal	Roth	120.1	
ROTIPHORESE®Gel 30 (37.5:1)	Roth	3029.1	

Salmon Sperm DNA sodium salt	Roth	5434.1
SDS pellets	Roth	8029.4
SigmaPrepTM spin column	Sigma Aldrich	SC1000
Sodium acetate	Roth	6773.1
Sodium chloride	VWR	27808.297
Sodium dihydrogen phosphate dihydrate	Roth	T879.1
Sodium hydroxide	VWR	VWRC28244.295
Soya peptone	Formedium	VPEP01
SuperScript™ III Reverse Transcriptase	Invitrogen™	18080093
T4 DNA Ligase	Thermo Scientific™	EL0016
T4 polynucleotide kinase	Thermo Scientific™	EK0032
T4 RNA Ligase 1 (ssRNA Ligase)	New England Biolabs	M0204
T4 RNA Ligase 2, truncated KQ	New England Biolabs	M0373
TEMED	Roth	2367.1
TRIS	Roth	5429.3
Trisodium citrate dihydrate >= 99%	Thermo Scientific Alfa Aesar	036439.A3
Trizma® hydrochloride solution	Sigma-Aldrich	T2319-1L
TRIzol™ Reagent	Invitrogen™	15596018
Tween® 20	Roth	9127.2
UltraPure™ Agarose	Invitrogen™	16500500
Uracil	Formedium	DOC0212
XbaI	Thermo Scientific™	ER0681
Yeast extract	Formedium	YEA03
Yeast Nitrogen Base without Amino Acids	Formedium	CYN0410
Yeast synthetic drop-out medium supplements	Sigma-Aldrich	Y2001-20G
Zirconia/Silica Beads	BioSpec	11079105z

Supplementary Table S5. List of Media used in this study.

Media	Ingredients
YPD	2 % w/v soya peptone, 1 % w/v yeast extract, 2 % w/v D(+)-Glucose
CSM complete	0.77 g/L complete supplement mixture, 6.9 g/L Yeast Nitrogen Base without Amino Acids, 20 g/L D(+)-Glucose
-LEU	1.4 g/L yeast synthetic drop-out medium supplements, 76 mg/L L-Histidin, 76 mg/L L-Tryptophan, 76 mg/L Uracil, 6.9 g/L Yeast Nitrogen Base without Amino Acids, 20 g/L D(+)-Glucose
-HIS	1.4 g/L yeast synthetic drop-out medium supplements, 76 mg/L L-Leucine, 76 mg/L L-Tryptophan, 76 mg/L Uracil, 6.9 g/L Yeast Nitrogen Base without Amino Acids, 20 g/L D(+)-Glucose
-URA	0.77 g/L complete supplement mixture drop out -URA, 6.9 g/L Yeast Nitrogen Base without Amino Acids, 20 g/L D(+)-Glucose
-TRP	1.4 g/L yeast synthetic drop-out medium supplements, 76 mg/L L-Leucine, 76 mg/L L-Histidin, 76 mg/L Uracil, 6.9 g/L Yeast Nitrogen Base without Amino Acids, 20 g/L D(+)-Glucose
For the corresponding plates	add 2 % w/v agar

Supplementary Table S6. List of buffers used in this study.

Buffer	Ingredients
PBS-Tween	9.93 g/L PBS, 1 % v/v Tween® 20
Western Blot lysis buffer	0.1 M NaOH, 0.05 M EDTA pH 8, 2 % w/v SDS, 2% v/v 2-mercaptoethanol
Loading buffer	0.25 M Tris-HCl pH 6.8, 50 % v/v glycerol, 0.05 % w/v bromophenol blue
SDS-running buffer	3 g/L Tris, 14.4 g/L Glycine, 1 g/L SDS
Transfer Buffer	3 g/L Tris, 14.4 g/L Glycine
5 % Milk	5 % w/v powdered milk in PBST
Mild stripping buffer pH 2.2	15 g/L Glycine, 1 g/L SDS, 1 % v/v Tween® 20
MOPS buffer pH 7	200 mM MOPS, 80 mM sodium acetate, 10 mM EDTA pH 8
10X SSC pH 7	1.5 M Sodium chloride, 0.15 M Trisodiumcitratedihydrate
Hybridization buffer	0.5 M Sodium phosphate, 1 mM EDTA pH 8, 7 % w/v SDS
Sodium phosphate pH 7	Mix 610 mL 1 M Sodium dihydrogen phosphate dihydrate with 390 mL 1 M di-Sodium hydrogen phosphate, adjust pH with NaOH
Washing solution	1 M Sodium phosphate, 1 mM EDTA pH 8, 1% w/v SDS
RIPA	25 mM HEPES pH 8, 150 mM NaCl, 1 mM EDTA, 0.5 % w/v sodium deoxycholate, 1 % v/v NP-40 alternative, 0.1 % w/v SDS
CRAC lysis buffer	50 mM Tris-HCl, pH 7.8, 150 mM NaCl, 0.1 % v/v Nonidet-P-40 substituent (Roche), 5 mM 2-mercaptoethanol
CRAC wash buffer I	50 mM Tris-HCl, pH 7.5, 300 mM NaCl, 0.1 % v/v Nonidet-P-40 substituent, 10 mM imidazole, 5 mM 2-mercaptoethanol, 6 M guanidinium hydrochloride
CRAC wash buffer II	50 mM Tris-HCl, pH 7.5, 50 mM NaCl, 0.1 % v/v Nonidet-P-40 substituent, 10 mM imidazole, 5 mM 2-mercaptoethanol
CRAC elution buffer	wash buffer II + 250 mM imidazole
CRAC high salt buffer	50 mM Tris-HCl, pH 7.8, 1 M NaCl, 0.1 % v/v Nonidet-P-40 substituent (Roche), 5 mM 2-mercaptoethanol

Supplementary Table S7. Polyacrylamide gel composition used in this study.

Polyacrylamide gel	Ingredients
10% running gel	water 2.37 mL, 1.5 M Tris-HCL pH 8.8 1.5 mL, 10% SDS 60 μL, 10% APS 60 μL, TEMED 6 μL, 30% acrylamide 2 mL
stucking gel	water 2.06 mL, 1 M Tris-HCL pH 6.8 375 μL, 10% SDS 30 μL, 10% APS 30 μL, TEMED 3 μL, 30% acrylamide 488 μL

Supplementary Table S8. List of devices used in this study.

Device	Company
Fixed angle rotor 1189-A	Hettich
Flexstation	Molecular Devices
FUSION Pulse TS	Vilber
Mikro 220R centrifuge	Hettich
Mini Hybrid 38	H. Saur
MiniSeq-System	Illumina
Multifuge X3R Centrifuge	Thermo Scientific™
NanoDrop™ 2000	Thermo Scientific™
Orbitrap Astral mass spectrometer	Thermo Scientific™
Owl™ A5 system	Thermo Scientific™
Gel-Documentation EBox VX5	Peqlab
Qubit™ 2 Fluorometer	Invitrogen™
Qubit™ 3 Fluorometer	Invitrogen™
ReproSil-Pur 120 C18-AQ	Dr. Maisch GmbH
Sonorex Super RK 31	Bandelin electronic
Swinging bucket rotor TX-750	Thermo Scientific™
Typhoon FLA9500	GE Healthcare
UV Stratalinker™ 1800	Stratagene
Vanquish Neo UHPLC system	Thermo Scientific™
Vari-X-Link	UVO3
Wet/Tank Blotting Systems	BioRad

Supplementary Table S9. List of barcoded 5′-linkers used for CRAC experiments.

Strain	Temperature	Replicate	5'-Linker
BY4741	30°C	1	L5Ad
Hel2-HTP	30°C	1	L5Ca
Hel2-HTP, *new1Δ::KanMX*	30°C	1	L5Ac
BY4741	30°C	2	L5Dc
Hel2-HTP	30°C	2	L5De
Hel2-HTP, *new1Δ::KanMX*	30°C	2	L5Da
BY4741	20°C	1	L5De
Hel2-HTP	20°C	1	L5Ea
Hel2-HTP, *new1Δ::KanMX*	20°C	1	L5Eb
New1-HTP	20°C	1	L5Ed
New1-HTP, *hel2Δ*	20°C	1	L5Ec
BY4741	20°C	2	L5Bc
Hel2-HTP	20°C	2	L5Bd
Hel2-HTP, *new1Δ::KanMX*	20°C	2	L5Da
New1-HTP	20°C	2	L5Db
New1-HTP, *hel2Δ*	20°C	2	L5Dc

Supplementary Table S10. Overview of nanopore sequencing libraries generated in this work.

Strain	Run	Replicate	Treatment	Barcode	fastq-sequences after adapter trimming	Aligned Reads
hel2Δ, xrn1Δ (1)	1	1	-	4	2,714,409	2,608,013
slh1Δ, xrn1Δ (1)	1	1	-	5	1,917,417	1,906,038
rqt4Δ, xrn1Δ (1)	1	1	-	6	2,724,735	2,713,872
dom34Δ, xrn1Δ (1)	1	1	-	7	2,906,515	2,872,494
cue2Δ, xrn1Δ (1)	1	1	-	8	3,250,797	3,191,156
hel2Δ, new1Δ, xrn1Δ (1)	1	1	-	10	1,849,777	1,814,629
slh1Δ, new1Δ, xrn1Δ (1)	1	1	-	11	1,681,706	1,667,079
rqt4Δ, new1Δ, xrn1Δ (1)	1	1	-	12	1,650,053	1,631,653
dom34Δ, new1Δ, xrn1Δ (1)	1	1	-	13	2,828,356	2,734,604
cue2Δ, new1Δ, xrn1Δ (1)	1	1	-	14	2,566,707	2,516,382
xrn1Δ (1)	2	1	-	5	3,426,304	3,376,904
hel2Δ, xrn1Δ (2)	2	2	-	6	3,170,636	3,120,320
slh1Δ, xrn1Δ (2)	2	2	-	7	2,351,920	2,317,039
rqt4Δ, xrn1Δ (2)	2	2	-	10	2,088,505	2,053,510
dom34Δ, xrn1Δ (2)	2	2	-	11	689,747	680,039
cue2Δ, xrn1Δ (2)	2	2	-	12	1,571,790	1,544,206
hel2Δ, slh1Δ, xrn1Δ (1)	2	1	-	13	1,823,354	1,790,165
hel2Δ, slh1Δ, xrn1Δ (2)	2	2	-	14	1,853,240	1,818,606
new1Δ, xrn1Δ (1)	2	1	-	15	2,306,348	2,256,651
hel2Δ, new1Δ, xrn1Δ (2)	2	2	-	16	1,652,786	1,616,311
slh1Δ, new1Δ, xrn1Δ (2)	2	2	-	17	1,435,397	1,392,413
rqt4Δ, new1Δ, xrn1Δ (2)	2	2	-	18	1,639,565	1,607,235
dom34Δ, new1Δ, xrn1Δ (2)	2	2	-	19	3,465,938	3,399,415
cue2Δ, new1Δ, xrn1Δ (2)	2	2	-	20	2,172,843	2,128,292
hel2Δ, new1Δ, slh1Δ, xrn1Δ (1)	2	1	-	21	2,080,842	2,029,153
hel2Δ, new1Δ, slh1Δ, xrn1Δ (2)	2	2	-	22	2,873,256	2,822,041
xrn1Δ (2)	3	2	-	4	2,747,695	2,705,385
hel2Δ, xrn1Δ (3)	3	3	-	6	2,508,212	2,468,888
slh1Δ, xrn1Δ (3)	3	3	-	7	1,984,302	1,955,351
rqt4Δ, xrn1Δ (3)	3	3	-	9	2,271,339	2,223,530
dom34Δ, xrn1Δ (3)	3	3	-	11	3,226,160	3,184,998
cue2Δ, xrn1Δ (3)	3	3	-	12	1,983,897	1,948,032
hel2Δ, slh1Δ, xrn1Δ (3)	3	3	-	13	3,146,284	3,055,450
new1Δ, xrn1Δ (2)	3	2	-	14	2,282,849	2,226,457
hel2Δ, new1Δ, xrn1Δ (3)	3	3	-	15	3,504,275	3,433,777
slh1Δ, new1Δ, xrn1Δ (3)	3	3	-	16	2,315,721	2,281,747
rqt4Δ, new1Δ, xrn1Δ (3)	3	3	-	17	2,227,184	2,185,262
dom34Δ, new1Δ, xrn1Δ (3)	3	3	-	18	2,090,425	2,060,480
cue2Δ, new1Δ, xrn1Δ (3)	3	3	-	19	2,838,575	2,798,548

**Strain**	**Run**	**Replicate**	**Treatment**	**Barcode**	**fastq-sequences after adapter trimming**	**Aligned Reads**
hel2Δ, new1Δ, slh1Δ, xrn1Δ (3)	3	3	-	20	2,201,934	2,170,285
ski2Δ (1)	4	1	+PNK, +IVPA	1	2,050,034	2,009,565
ski2Δ, slh1Δ (1)	4	1	+PNK, +IVPA	3	2,438,609	2,395,757
rqt4Δ, ski2Δ (1)	4	1	+PNK, +IVPA	4	2,102,073	2,050,661
cue3Δ, ski2Δ (1)	4	1	+PNK, +IVPA	5	2,347,087	2,299,200
rqt4Δ, ski2Δ, cue3Δ (1)	4	1	+PNK, +IVPA	6	4,072,178	3,994,932
new1Δ, ski2Δ (1)	4	1	+PNK, +IVPA	7	2,248,387	2,202,936
new1Δ, ski2Δ, slh1Δ (1)	4	1	+PNK, +IVPA	9	2,677,053	2,605,656
rqt4Δ, new1Δ, ski2Δ (1)	4	1	+PNK, +IVPA	10	2,817,224	2,752,479
cue3Δ, new1Δ, ski2Δ (1)	4	1	+PNK, +IVPA	11	2,495,794	2,429,099
rqt4Δ, new1Δ, ski2Δ, cue3Δ (1)	4	1	+PNK, +IVPA	12	2,953,622	2,888,687
ski2Δ (2)	4	2	+PNK, +IVPA	13	3,075,608	3,018,704
ski2Δ, slh1Δ (2)	4	2	+PNK, +IVPA	15	2,999,185	2,845,435
rqt4Δ, ski2Δ (2)	4	2	+PNK, +IVPA	16	1,616,594	1,557,337
cue3Δ, ski2Δ (2)	4	2	+PNK, +IVPA	17	2,286,708	2,172,414
rqt4Δ, ski2Δ, cue3Δ (2)	4	2	+PNK, +IVPA	18	3,921,137	3,825,985
new1Δ, ski2Δ (2)	4	2	+PNK, +IVPA	19	2,778,591	2,679,284
new1Δ, ski2Δ, slh1Δ (2)	4	2	+PNK, +IVPA	21	4,154,523	4,022,673
rqt4Δ, new1Δ, ski2Δ (2)	4	2	+PNK, +IVPA	22	3,239,591	3,105,033
cue3Δ, new1Δ, ski2Δ (2)	4	2	+PNK, +IVPA	23	2,899,501	2,773,473
rqt4Δ, new1Δ, ski2Δ, cue3Δ (2)	4	2	+PNK, +IVPA	24	2,951,628	2,824,217
**Strain**	**Run**	**Replicate**	**Treatment**	**Barcode**	**fastq-sequences after adapter & poly(A) tail trimming**	**Aligned Reads**
wildtype (1)	5	1	-	10	1,180,280	1,176,473
wildtype (2)	5	2	-	11	1,465,381	1,462,165
wildtype (3)	5	3	-	12	1,226,871	1,224,204
new1Δ (1)	5	1	-	13	1,415,371	1,410,469
new1Δ (2)	5	2	-	14	348,470	346,507
new1Δ (3)	5	3	-	15	1,174,439	1,169,732

Supplementary Table S11. List of NGD candidates.

Gene	C-terminal codon	*new1Δ, ski2Δ*	*new1Δ, slh1Δ, ski2Δ*	*new1Δ, rqt4Δ, ski2Δ*	*new1Δ, cue3Δ, ski2Δ*	*new1Δ, rqt4Δ, cue3Δ, ski2Δ*	*new1Δ, xrn1Δ*	*new1Δ, slh1Δ, xrn1Δ*	*new1Δ, rqt4Δ, xrn1Δ*	*new1Δ, dom34Δ, xrn1Δ*	*new1Δ, cue2Δ, xrn1Δ*	*new1Δ, hel2Δ, xrn1Δ*	*new1Δ, hel2Δ, slh1Δ, xrn1Δ*
ACC1	strong	-	-	-	-	-	-	x	x	-	-	-	-
ACF4	strong	-	-	-	-	-	-	x	-	-	-	-	-
ACO1	strong	-	x	x	x	x	x	x	x	-	-	-	-
ACS2	strong	-	x	-	-	x	x	x	x	-	-	-	-
ADH1	strong	x	x	x	x	x	x	x	x	x	-	-	-
ADH3	strong	-	x	-	-	-	x	x	x	-	-	-	-
ADO1	strong	x	x	x	x	x	-	-	x	-	-	-	-
AIM17	strong	-	-	-	-	-	x	x	x	-	-	-	-
ALD3	strong	-	-	-	-	-	x	x	x	-	-	-	-
ALD5	strong	-	-	-	-	-	x	x	x	-	-	-	-
APT1	strong	-	x	-	-	-	-	-	-	-	-	-	-
ARC1	strong	-	x	x	-	-	-	-	x	-	-	-	-
ARO10	strong	-	-	-	-	-	-	x	x	x	-	-	-
ARO3	strong	-	-	x	-	-	-	x	x	-	-	-	-
ARO4	strong	-	x	x	x	x	-	x	x	-	-	-	-
ARO8	strong	-	-	-	-	-	-	x	x	-	-	-	-
ATG8	strong	-	x	-	-	-	-	-	-	-	-	-	-
BMH2	strong	-	x	x	-	x	x	x	x	-	-	-	-
BRF1	strong	-	-	-	-	-	-	x	x	-	-	-	-
CAB2	strong	-	-	-	-	-	-	-	x	-	-	-	-
CAM1	strong	-	-	-	-	-	x	x	x	-	-	-	-
CCT4	strong	-	-	-	-	-	-	x	-	-	-	-	-
CDC10	strong	-	-	-	-	-	-	x	x	-	-	-	-
CDC12	strong	-	-	-	-	-	-	x	x	-	-	-	-
CDC20	strong	-	-	-	-	-	-	x	-	-	-	-	-
CDC3	strong	-	-	-	-	-	-	-	x	-	-	-	-
CDC73	strong	-	-	-	-	-	-	x	-	-	-	-	-
CLU1	strong	-	-	-	-	-	-	-	-	x	-	-	-
CMD1	strong	-	-	-	-	-	-	x	-	-	-	-	-
CRS1	strong	-	-	-	-	-	-	x	-	-	-	-	-
CYC7	strong	-	-	-	-	-	-	x	x	-	-	-	-
DCC1	strong	-	-	-	-	-	-	x	x	-	-	-	-
DCS1	strong	-	-	-	-	-	-	x	x	-	-	-	-
DRS1	strong	-	-	-	-	-	-	x	-	-	-	-	-
EGD2	strong	x	x	x	x	x	-	-	-	-	-	-	-
ELO2	strong	-	-	-	-	-	x	x	x	-	-	-	-
ERG13	strong	-	-	-	-	-	x	x	x	-	-	-	-
ERG20	strong	-	x	-	-	x	x	x	x	-	-	-	-
ERV41	strong	-	-	-	-	-	-	-	x	-	-	-	-
FAS2	strong	-	-	-	-	-	-	x	x	-	-	-	-
FCF2	strong	-	-	-	-	-	-	x	-	-	-	-	-
FIS1	strong	-	-	-	-	-	-	x	-	-	-	-	-
FKS1	strong	-	-	-	-	-	-	x	x	-	-	-	-
FMP48	strong	-	-	-	-	-	-	x	-	-	-	-	-
FPR4	strong	-	-	-	-	-	-	x	x	-	-	-	-
FUM1	strong	-	-	-	-	-	-	x	x	-	-	-	-
GLE2	strong	-	-	-	-	-	-	x	x	-	-	-	-
GPM1	strong	x	x	x	x	x	x	x	x	-	-	-	-
GUS1	strong	-	-	-	-	-	-	-	x	-	-	-	-
HRP1	strong	-	-	-	-	-	-	x	x	-	-	-	-
HRT1	strong	-	x	-	-	-	-	-	-	-	-	-	-
HXT1	strong	-	-	-	x	-	x	x	x	-	-	-	-
HXT3	strong	-	-	x	x	x	x	x	x	-	-	-	-
HXT6	strong	-	-	x	-	-	x	-	-	-	-	-	-
HXT7	strong	-	-	-	x	-	x	x	x	x	-	-	-
INA1	strong	-	-	-	-	-	x	x	-	-	-	-	-
IRC22	strong	x	-	x	-	-	-	-	-	-	-	-	-
KEL3	strong	-	-	-	-	-	-	-	x	-	-	-	-
LAP3	strong	-	-	-	-	-	x	x	x	-	-	-	-
LSC1	strong	-	-	-	-	-	-	x	x	-	-	-	-
LYS21	strong	-	-	x	x	x	-	x	x	-	-	-	-

Gene	C-terminal codon	*new1Δ, ski2Δ*	*new1Δ, slh1Δ, ski2Δ*	*new1Δ, rqt4Δ, ski2Δ*	*new1Δ, cue3Δ, ski2Δ*	*new1Δ, rqt4Δ, cue3Δ, ski2Δ*	*new1Δ, xrn1Δ*	*new1Δ, slh1Δ, xrn1Δ*	*new1Δ, rqt4Δ, xrn1Δ*	*new1Δ, dom34Δ, xrn1Δ*	*new1Δ, cue2Δ, xrn1Δ*	*new1Δ, hel2Δ, xrn1Δ*	*new1Δ, hel2Δ, slh1Δ, xrn1Δ*
MAP1	strong	-	-	-	-	-	-	-	x	-	-	-	-
MBF1	strong	-	-	-	-	-	-	x	-	-	-	-	-
MDH1	strong	x	x	x	-	x	-	x	x	-	-	-	-
MGM101	strong	-	-	-	-	-	-	x	-	-	-	-	-
MRPL35	strong	-	-	-	-	-	x	x	-	-	-	-	-
MTD1	strong	-	-	-	-	-	-	-	x	-	-	-	-
NCE103	strong	-	-	-	-	-	-	x	x	-	-	-	-
NIP1	strong	-	-	-	-	-	x	x	-	-	-	-	-
NOP1	strong	-	-	-	-	x	x	x	x	-	-	-	-
NOP12	strong	-	-	-	-	-	-	-	x	-	-	-	-
NOP2	strong	-	-	-	-	-	-	x	x	-	-	-	-
NOP58	strong	-	x	-	-	-	-	-	-	-	-	-	-
NPC2	strong	x	x	x	x	x	-	x	-	-	-	-	-
NPL3	strong	-	-	x	x	x	-	x	x	-	-	-	x
NUS1	strong	-	-	-	-	-	x	-	-	-	-	-	-
OM14	strong	-	-	x	-	-	-	-	-	-	-	-	-
PDR5	strong	-	-	-	-	-	x	-	x	-	-	-	-
PET9	strong	-	x	x	x	x	-	-	-	-	-	-	-
PFK1	strong	-	-	-	-	-	-	-	x	-	-	-	-
PGK1	strong	x	x	x	x	x	-	x	x	-	-	-	-
PNC1	strong	x	x	x	x	x	-	x	-	-	-	-	-
PRO3	strong	-	-	-	-	-	-	-	x	-	-	-	-
PRX1	strong	-	-	-	-	-	-	x	x	-	-	-	-
PUF6	strong	-	-	-	-	-	-	-	x	-	-	-	-
QRI1	strong	-	-	-	-	-	-	x	x	-	-	-	-
RBG2	strong	-	-	-	-	-	-	x	x	-	-	-	-
RDI1	strong	-	-	-	-	-	-	x	-	-	-	-	-
RIB3	strong	-	-	x	-	-	-	-	-	-	-	-	-
ROK1	strong	-	-	-	-	-	-	x	-	-	-	-	-
RPA49	strong	-	-	-	-	-	-	x	x	-	-	-	-
RPG1	strong	-	-	-	-	-	x	x	x	x	-	-	-
RPL11A	strong	-	-	x	-	-	-	-	-	-	-	-	-
RPL11B	strong	x	x	x	x	x	-	x	-	-	-	-	-
RPL13A	strong	x	x	x	x	x	-	-	-	-	-	-	-
RPL13B	strong	-	-	x	-	-	-	-	-	-	-	-	-
RPL15A	strong	x	x	x	-	x	-	x	-	-	-	-	-
RPL24A	strong	x	x	x	x	x	-	-	-	-	-	-	-
RPL24B	strong	-	-	x	-	-	-	-	-	-	-	-	-
RPL34A	strong	-	x	x	x	x	-	-	-	-	-	-	-
RPL40B	strong	x	-	-	-	-	-	-	-	-	-	-	-
RPS16A	strong	x	x	x	-	-	-	-	-	-	-	-	-
RPS16B	strong	x	x	x	x	-	-	-	-	-	-	-	-
RPS21A	strong	x	-	-	-	-	-	-	-	-	-	-	-
RPS5	strong	x	x	x	x	x	-	-	-	-	-	-	-
RPT3	strong	-	-	-	-	-	-	x	x	-	-	-	-
RRP3	strong	-	-	-	-	-	-	-	x	-	-	-	-
RSM23	strong	-	-	-	-	-	-	x	-	-	-	-	-
RTS3	strong	-	-	-	-	-	x	x	x	-	-	-	-
RVS161	strong	-	-	-	-	-	-	x	-	-	-	-	-
SAC6	strong	-	-	-	-	-	x	x	x	-	-	-	-
SEC14	strong	-	-	-	-	-	-	x	x	-	-	-	-
SEC24	strong	-	-	-	-	-	-	x	x	-	-	-	-
SFA1	strong	-	-	-	-	-	-	x	x	-	-	-	-
SKI6	strong	-	-	-	-	-	-	-	x	-	-	-	-
SKI8	strong	-	-	-	-	-	-	x	-	-	-	-	-
SKP1	strong	-	-	-	-	-	-	x	x	-	-	-	-
SPN1	strong	-	-	-	-	-	-	x	-	-	-	-	-
SRP68	strong	-	-	-	-	-	-	x	-	-	-	-	-
SSB1	strong	x	x	x	x	x	-	x	x	-	-	-	-
SSB2	strong	x	x	x	x	x	-	x	x	-	-	-	-
SSO1	strong	-	-	-	-	-	-	x	-	-	-	-	-
TEF1	strong	x	x	x	x	x	x	x	x	-	-	-	-
TEF2	strong	x	x	x	x	x	-	x	x	-	-	-	-
TEF4	strong	x	x	x	x	x	-	x	x	-	-	-	-

Gene	C-terminal codon	*new1Δ, ski2Δ*	*new1Δ, slh1Δ, ski2Δ*	*new1Δ, rqt4Δ, ski2Δ*	*new1Δ, cue3Δ, ski2Δ*	*new1Δ, rqt4Δ, cue3Δ, ski2Δ*	*new1Δ, xrn1Δ*	*new1Δ, slh1Δ, xrn1Δ*	*new1Δ, rqt4Δ, xrn1Δ*	*new1Δ, dom34Δ, xrn1Δ*	*new1Δ, cue2Δ, xrn1Δ*	*new1Δ, hel2Δ, xrn1Δ*	*new1Δ, hel2Δ, slh1Δ, xrn1Δ*
TFS1	strong	-	-	-	-	-	-	x	x	-	-	-	-
TIF3	strong	-	-	-	-	-	x	x	x	-	-	-	-
TMH18	strong	-	-	x	-	-	-	-	-	-	-	-	-
TOS7	strong	-	-	-	-	-	-	x	-	-	-	-	-
TPN1	strong	-	-	-	-	-	x	-	x	-	-	-	-
TSA1	strong	x	x	x	x	x	-	x	x	-	-	-	-
UBC6	strong	-	-	-	-	-	-	x	-	-	-	-	-
VMA13	strong	-	-	x	-	-	-	-	-	-	-	-	-
VRG4	strong	-	-	-	-	-	x	x	x	-	-	-	-
YKE2	strong	-	-	-	-	-	-	x	-	-	-	-	-
YMR027W	strong	-	-	-	-	-	-	x	-	-	-	-	-
YNL134C	strong	-	-	-	-	-	x	x	x	-	-	-	-
YOR283W	strong	-	-	-	-	-	-	x	x	-	-	-	-
YRA1	strong	-	-	-	-	-	-	x	-	-	-	-	-
BFR1	mild	-	-	-	-	-	-	x	-	-	-	-	-
CDC25	mild	-	-	-	-	-	-	x	-	-	-	-	-
CYS3	mild	-	-	-	-	-	-	-	x	-	-	-	-
LSC2	mild	-	-	-	-	-	-	x	-	-	-	-	-
MET6	mild	-	-	-	-	-	-	-	x	-	-	-	-
NSG1	mild	-	-	-	-	-	x	-	-	-	-	-	-
OYE2	mild	-	-	-	-	-	-	x	x	-	-	-	-
RSA3	mild	-	-	-	-	-	-	-	-	x	-	-	-
STI1	mild	-	x	-	-	-	-	x	x	-	-	-	-
UBI4	mild	-	-	-	-	-	-	x	-	-	-	-	-
ACH1	not	-	-	-	-	-	-	-	x	-	-	-	-
ATP1	not	-	x	x	-	-	-	x	x	-	-	-	-
GAS1	not	x	-	-	-	-	-	-	-	-	-	-	-
IES5	not	-	-	-	-	-	-	x	-	-	-	-	-
LSP1	not	-	-	-	-	-	x	-	-	-	-	-	-
LYS20	not	-	-	x	-	-	-	-	-	-	-	-	-
PAU7	not	-	-	-	-	-	-	x	x	-	-	-	-
PDA1	not	-	-	x	-	-	-	-	-	-	-	-	-
PIL1	not	-	-	x	-	-	-	-	-	-	-	-	-
PMC1	not	-	-	-	-	-	-	-	x	-	-	-	-
PRC1	not	-	-	x	-	-	-	-	-	-	-	-	-
RPL19B	not	-	-	x	-	-	-	-	-	-	-	-	-
RPL21B	not	-	-	x	-	-	-	-	-	-	-	-	-
RPL35B	not	-	-	-	-	x	-	-	-	-	-	-	-
RPL8A	not	-	-	x	-	-	-	-	-	-	-	-	-
RPS11A	not	-	-	x	-	-	-	-	-	-	-	-	-
RPS14A	not	-	-	x	-	-	-	-	-	-	-	-	-
RPS4A	not	-	-	x	-	-	-	-	-	-	-	-	-
RRP7	not	-	-	-	-	-	-	x	-	-	-	-	-
SHM2	not	-	-	x	-	x	-	-	x	-	-	-	-
SSE1	not	-	x	-	-	-	-	-	-	-	-	-	-
VMA2	not	-	-	x	-	-	-	-	-	-	-	-	-
YRB1	not	-	x	-	-	-	-	x	-	-	-	-	-
Discarded candidates													
NEW1	artefacts due to deletion	-	-	-	-	-	x	x	x	x	x	x	x
YLR156C-A	rRNA	x	x	-	-	x	-	-	-	-	-	-	-
YLR157C-C	overlap-	-	-	-	x	-	-	-	-	-	-	-	-
YLR154C-G	ping	-	-	-	-	-	-	x	-	-	-	-	-
RPL37B	short gene with intron	-	-	x	-	-	-	-	-	-	-	-	-
RPS11A		-	-	x	-	-	-	-	-	-	-	-	-
RPL42A		-	-	-	-	x	-	-	-	-	-	-	-
YBR027C	close to neighboring CDS	-	-	x	-	-	-	-	-	-	-	-	-

Supplementary Table S12: Overview of RNA types of RNA that non-collapsed reads were aligned to, comparing New1-CRAC to Hel2-CRAC. New1 binds roughly similar amounts of mRNA and rRNA as Hel2 does, but shows higher binding for tRNA than Hel2. Non-collapsed data are shown here, as collapsing leads to underestimation of highly abundant tRNAs and rRNAs, due to the nature of our unique molecule identifiers.

	New1, rep. 1	New1, rep. 2	New1, *hel2Δ*, rep. 1	New1, *hel2Δ*, rep. 2	Hel2, rep. 1	Hel2, rep. 2	Hel2, *new1Δ*, rep. 1	Hel2, *new1Δ*, rep. 2
mRNA	40%	43%	41%	42%	52%	49%	44%	36%
rRNA	35%	34%	41%	32%	37%	40%	44%	54%
tRNA	20%	16%	14%	19%	7%	7%	8%	7%
Other RNAs	5%	7%	5%	7%	5%	4%	4%	3%

Supplementary Table S13. List of different tRNAs bound by New1 in different samples, analyzed by type. Reads mapping to different alleles of identical tRNAs were combined. Dark grey: tRNAs decoding strongly affected codons, medium grey: tRNAs decoding mildly affected codons, light grey: tRNAs decoding non-affected lysine and arginine codons.

tRNA type	New1, rep. 1	New1, rep. 2	New1, *hel2Δ*, rep. 1	New1, *hel2Δ*, rep. 2
tA(AGC)	0.8%	0.5%	0.7%	0.4%
tA(UGC)	6.9%	10.4%	8.5%	9.4%
tC(GCA)	0.9%	0.9%	0.5%	0.8%
tD(GUC)	2.7%	3.4%	6.1%	3.8%
tE(CUC)	5.9%	4.8%	7.2%	4.3%
tE(UUC)	18.8%	14.2%	21.4%	11.3%
tF(GAA)	0.04%	0.02%	0.1%	0.0%
tG(CCC)	1.7%	0.8%	0.8%	0.8%
tG(GCC)	16.2%	11.4%	13.7%	8.4%
tG(UCC)	3.6%	1.2%	2.0%	0.9%
tH(GUG)	1.9%	1.4%	1.6%	1.4%
tI(AAU)	1.2%	1.0%	0.8%	0.6%
tI(UAU)	0.1%	0.1%	0.1%	0.1%
tK(CUU)	3%	2.3%	3.0%	2.6%
tK(UUU)	0.4%	0.2%	0.23%	0.2%
tL(CAA)	3.5%	1.2%	1.9%	1.2%
tL(GAG)	0.03%	0.01%	0.03%	0.02%
tL(UAA)	0.8%	0.5%	0.3%	0.4%
tL(UAG)	0.6%	0.4%	0.6%	0.3%
tM(CAU)	0.4%	0.3%	0.3%	0.2%
tN(GUU)	0.6%	0.2%	0.8%	0.7%
tP(AGG)	0.6%	0.02%	0.4%	0.1%
tP(UGG)	0.3%	0.2%	0.2%	0.1%
tQ(CUG)	1.0%	0.2%	0.7%	0.3%
tR(ACG)	1.0%	0.5%	1.0%	0.3%
tR(CCG)	1.5%	0.7%	1.1%	1.1%
tR(CCU)	6.2%	28.9%	6.5%	37.7%
tR(UCU)	6.0%	2.4%	5.0%	2.7%
tS(AGA)	3.9%	5.4%	4.6%	2.4%
tS(CGA)	0.1%	0.1%	0.2%	0.1%
tS(GCU)	0.8%	0.5%	1.0%	0.4%
tS(UGA)	2.1%	1.8%	2.0%	2.4%
tT(AGU)	0.6%	0.2%	0.5%	0.2%
tT(CGU)	0.7%	0.9%	0.8%	0.8%
tT(UGU)	0.3%	0.1%	0.3%	0.07%
tT(XXX)	0.0%	0.0%	0.0%	0.0%
tV(AAC)	0.9%	0.5%	0.8%	0.6%
tV(CAC)	0.9%	0.7%	1.0%	0.7%
tV(UAC)	1.3%	1.1%	0.6%	0.8%
tW(CCA)	0.5%	0.1%	0.4%	0.1%
tW(UCA)	0.0%	0.0%	0.02%	0.0%
tX(XXX)	0.3%	0.2%	0.3%	0.1%
tY(GUA)	1.3%	0.6%	2.2%	1.0%
total tRNA in library	20%	16%	14%	19%

Supplementary Methods

Construction of Plasmids

New1 overexpression plasmid. For the construction of a New1 overexpressing plasmid, the coding sequence encoding New1-FLAG was amplified from genomic DNA of a genomically, C-terminally tagged New1-FLAG-TEV-ProteinA$_2$ expressing strain, with Phusion high fidelity polymerase and oligonucleotides harboring XbaI restriction sites as 5′-overhangs. The PCR product was purified after agarose gel electrophoresis followed by DNA fragment extraction. Next, XbaI digestion was performed overnight at 37°C (0.0075 U/µL XbaI, 1*x* Tango-Buffer (Invitrogen), 50-75 ng/µL DNA). The plasmid pKK148 (10) was digested in the same way. To prevent backbone self-ligation, 0.05 U/µL Fast AP alkaline phosphatase was added to an aliquot of the digested backbone and incubated for 25 min at 37°C. After heat inactivation at 75°C for 10 min, purification was performed for both PCR product and plasmid backbone by agarose gel electrophoresis followed by DNA fragment extraction. The ligation was conducted with an insert to backbone molar-ratio of 5:1 for the New1-FLAG overexpression vector and non-dephosphorylated backbone was used for empty vector ligation for 1 h at room temperature (1x Ligase buffer, 0.075 U/µL T4 DNA Ligase). The ligation product was transformed into chemically competent DH5α *E. coli* cells and selected on 100 µg/mL ampicillin plates. Colonies were restreaked and validated *via* PCR. Plasmids were isolated (according to manufacturer's protocol with GeneJET Plasmid Miniprep Kit) and the coding sequence of *NEW1-FLAG* was validated by Sanger sequencing (Eurofins Genomics, Mix2Seq). The empty vector was also validated by Sanger-sequencing.

FLAG-TEV-ProteinA$_2$ (FTP) tagging plasmid. For FLAG-TEV-ProteinA$_2$ tagging the template plasmid pBS1539-FTP was generated *via* site-directed mutagenesis from pBS1539-HTP (11), which was used for His$_6$-TEV-ProteinA$_2$ tagging. For site-directed mutagenesis, pBS1539-HTP was amplified with Phusion high fidelity polymerase, excluding the His$_6$ sequence, using 5′-phosphorylated primers, which included the sequence encoding the FLAG peptide as overhangs. Ligation, transformation, and validation were performed as described above.

Readthrough-reporter. The coding sequence of GFP was amplified from GFP-TEV-R12-RFP construct (pGTRR) (1, 16) with a forward primer including an XbaI restriction site. The reverse primer was used to manipulate the identity of the C-terminal GFP codon, either to keep the native lysine (AAA) codon or to mutate it to an 'AAG' codon. Furthermore, a stop codon

(UAA) was introduced, followed by a glycine spacer (GGA) and either 1, 2, or no adenosine nucleotides to construct three different vectors, each with the 3x-FLAG-tag in a different one of the three reading frames downstream of the stop codon. The PCR products were purified by agarose gel electrophoresis followed by DNA fragment extraction and used for a second amplification with the same forward primer and a second reverse primer to complete the 3x-FLAG sequence and insert an aspartic acid spacer (GAT) to avoid a lysine (AAG) codon in front of the 3x-FLAG stop codon (UAA). This reverse primer also includes another XbaI digestion site. The remaining cloning was performed as described for the construction of the New1 overexpression vector. As a control construct, the C-terminal GFP codon was omitted, and the 3x-FLAG-tag was placed in the same reading frame as the GFP sequence.

Construction of strains

Strains were cultured in YPD, at 30°C and 220 rpm. Gene deletion or tagging, as well as plasmid transformation were performed using the "LiAc/SS Carrier DNA/PEG Method" (17). Genes were replaced by either KanMX (using pFA6a-kanMX (12) or pYM18 (14)) or HphMX cassette (using pyM20 (14)) or the *LEU2* gene (from plasmid YEp181-CUP1-His-Ubi (15)) and validated *via* PCR. C-terminal tagging of genes was performed by amplifying the tagging cassette from pBS1539-HTP or pBS1539-FTP with Phusion high fidelity polymerase and oligonucleotides containing 5′-overhangs which anneal to the 5′- and 3′-region, excluding the stop codon of the corresponding gene of interest. Tagging was validated *via* PCR and Western Blot, probed with peroxidase anti-peroxidase soluble complex antibody (1:2,000) overnight at 4°C in 5% w/v milk PBST.

Western Blot

10% SDS-polyacrylamide gels were run for 15 min at 75 V, followed by 100 V until the ladder was fully resolved. Proteins were transferred onto nitrocellulose membrane for 75 min at 100 V. The quality of the samples and the transfer was checked using Ponceau stain and blocked with 5% w/v milk. Membranes on which Pgk1 was to be quantified were first probed for tubulin as a loading control, incubating overnight at 4°C with anti-alpha Tubulin antibody (1:10,000) and for 2 h at room temperature with anti-Rabbit IgG HRP antibody (1:5,000) in 5% w/v milk PBST. After washing three times 5 min with 1x PBST, membranes were visualized with sufficient ECL solution on the FUSION Pulse TS (Vilber) system. Membranes were then stripped three times for 15 min in total with mild stripping buffer (Supplementary Table S6)

and washed three times each for 5 min, with PBS and PBST. Then, membranes were probed with anti-PGK1 antibody (1:10,000) at 4°C overnight, followed by 2 h incubation with anti-Mouse (GAM)-HRP conjugate antibody (1:20,000) in 5% w/v milk in PBST. Signals were quantified using ImageJ (18) and Pgk1 levels were normalized to Tubulin levels. For visualization of the readthrough reporter, anti-FLAG antibody (1:1,000) or anti-GFP antibody (1:1,000) was used, both followed by secondary anti-Mouse (GAM)-HRP conjugate antibody (1:20,000). Pgk1 was probed as described above.

Northern Blot

For RNA preparation, 150 µL zirconia beads were added per sample, vortexed 30 s at 3,000 rpm and kept on ice for at least 30 s between rounds, in a total of 10 rounds. 150 µL ROTI®C/I was added to each sample, briefly vortexed and kept on ice for 2 min. After centrifugation at 4°C for 10 min at 18,620 xg, the aqueous phase was recovered, followed by a second acidic phenol/chloroform extraction (each 200 µL). RNA was precipitated by adding 1/10 volume 3 M sodium acetate pH 5.2 and 1 volume 2-propanol and samples were kept at -20°C for 1 h. Next, RNA was pelleted at 18,620 xg at 4°C for 20 min, washed twice with 70% v/v ethanol, air-dried for 5 min, dissolved in ultrapure water and RNA concentration was measured with the NanoDrop™ 2000 system. Equal amounts of NorthernMax™-Gly Sample Loading Dye and 0.15 µg/µL additional ethidium bromide were added to the samples and incubated 30 min at 50°C. Up to 10 µg total RNA and 2 µL Riboruler low range ladder were loaded on a 1.4% w/v agarose MOPS gel in the Owl™ A5 system. The gel was run with MOPS buffer pH 7 at 50 V for 18-19 h. RNA was transferred using the capillary-based transfer method with 10x SSC pH 7 onto a nylon membrane. Afterwards, RNA was immobilized by crosslinking twice at 1,200 'µJoules x 100' doses with UV Stratalinker™ 1800. The membrane was blocked with hybridization buffer and 2 mg salmon sperm DNA for 1 h at 42-57°C (Hybrid Mini 38, H. Saur). Oligonucleotides were phosphorylated with γ-^{32}P-ATP (1.6 pmol oligonucleotide, 1x reaction buffer A, 1.6 pmol γ-^{32}P-ATP, 0.5 U/µL T4 PNK) for 30 min at 37°C. EDTA pH 8.0 was added to stop the reaction at a final concentration of 24 mM and heat inactivation was performed at 75°C for 10 min, without further purification. Blots were probed with 5'-^{32}P-phosphorylated oligonucleotides and fresh hybridization buffer overnight. The membrane was washed three times (up to 45 min) with washing solution, and a storage phosphor screen was exposed to the membrane and visualized with the Typhoon FLA9500

system. If required, membranes were stripped twice for 1 h with 0.5% w/v SDS at 65°C, blocked and re-probed as described before. Signals were quantified with ImageJ.

Supplementary Text S1

Readthrough reporter assay reveals no increase in stop codon readthrough upon NEW1 deletion. To assess potential readthrough in the absence of New1, we designed a reporter, employing GFP with a native terminal 'AAA' (strongly affected) or 'AAG' (not affected) codon followed by a 'UAA' stop codon, a glycine G(GGA) spacer to avoid any potential influence of the stop codon context that might otherwise cause variations (19, 20) between the different reading frame constructs and a 3xFLAG-tag, either in-frame, in the +1 or in the -1 frame (Supplementary Figure S4). The levels of FLAG (and hence readthrough in specific reading frames) were determined by Western blot and compared to a positive control without a stop codon. In our assay, we did not detect significant levels (all samples <0.5% of control, according to FLAG signals) of stop codon readthrough. Even more importantly, we did not observe any differences between wildtype and *new1Δ*.

mRNAs with increased stop codon readthrough upon NEW1 deletion do not exhibit stronger 3′-UTR enrichment of Hel2 than other strongly affected mRNAs. While writing our initial manuscript, a report was published (3) which showed that readthrough may occur in the absence of New1, but only under very specific conditions, where the affected C-terminal codon is followed by the weak stop codon 'UGA'. This weak stop codon had indeed already been shown to be underrepresented in the subset of queuing-prone mRNAs(2). Other combinations did not allow for marked stop codon readthrough in the dual luciferase assay employed in that study, validating the findings from our reporter assay, in which the affected codon was followed by the strong stop codon 'UAA', and followed by the nucleotide 'G', generating a strong termination context (19, 20). To exclude that the 3′-UTR enrichment of Hel2 was observed solely due to mRNAs in which the strongly affected codon is followed by the weak 'UGA' stop codon and a nucleotide 'C', we compared 3′-UTR enrichment in *new1Δ* over wildtype for this subset (20 mRNAs) to the subset where the strongly affected codon is followed by the stronger stop codons 'UAA' or 'UAG' and/or a +1 nucleotide 'not C' (154 mRNAs for 'UGA, not C'; 60 mRNAs for 'not UGA, C'; and 434 mRNAs for 'not UGA, not C'). 3′-UTR enrichment was observed for all subgroups, although in the 'UGA, C' subgroup, it was only detectable at 20°C, but not 30°C (Supplementary Figure S5). Here, it is possible that a certain level of stop codon readthrough on such mRNAs might reduce the number of ribosome collisions, and hence Hel2 recruitment. We conclude that stop codon readthrough does not appear as a likely cause for the majority of Hel2 binding seen in 3′-UTRs.

Published 5PSeq and RiboSeq data do not reveal significant stop codon readthrough or re-initiation in 3′-UTRs of strongly affected mRNAs. However, this does not fully exclude the possibility that ribosomes might re-initiate translation in the 3′-UTR, as previously observed (21), and that resulting translation problems within the 3′-UTR can lead to Hel2 recruitment. Kasari et al. (2) previously tested for 3′-UTR coverage and 3-nt periodicity in the 3′-UTRs of queuing-prone mRNAs and did not observe any convincing periodicity either in a metagene analysis focusing on the 100 most queuing-prone mRNAs in their dataset, or in single mRNA analyses of *BMH2* and *YNL247W (CRS1)*. We re-analysed those data and also found increased ribosome coverage of 3′-UTRs in strongly affected mRNAs, and to some extent in mildly affected mRNAs when New1 was lacking, in RiboSeq data (Supplementary Figure S6, but not 5PSeq data (Supplementary Figure S8). However, we also did not detect any 3-nt periodicity in the metagene plots (Supplementary Figure S6). Yet, these data resemble data from previous studies under conditions that led to stop codon readthrough [Wangen & Green, Schuller], so these data also do not support the opposite. To exclude that different mRNAs might be decoded in different reading frames, we also analyzed several highly expressed mRNAs from the strongly affected group, separately and found no convincing 3-nt periodicity in *ADH1*, *PGK1*, *NOP1* (Supplementary Figure S7), and *GPM1*, *RPS5* or *TEF4* (not shown). Here, the position of ribosome peaks (P-sites) in New1-lacking strains also did not coincide with positions of stop codons or potential stalling sequences. Some of the examples (*PGK1* and *NOP1*) did not even contain a methionine codon upstream of ribosome peaks, so that re-initiation should not be possible here. Interestingly, in contrast to RiboSeq data, published 5PSeq data (3) did not reveal any increase in ribosome coverage in the 3′-UTR of strongly affected mRNAs for New1-lacking strains (Supplementary Figure S8).

In light of this, although we still cannot fully exclude the possibility that stop codon readthrough or re-initiation occur and are responsible for at least part of the Hel2 recruitment, we favour the hypothesis that Hel2 crosslinking in 3′-UTRs in our system is mainly driven by Hel2 binding to collided ribosomes at the stop codon, and making contacts with downstream 3′-UTR.

Additional hints towards Hel2 bound to collided ribosomes at stop codons making contacts with downstream portions of 3′-UTRs. This is supported by two additional findings: (i) Our re-analysis of 5PSeq data (Fig 2D) shows that, in a major population of strongly affected mRNAs, the region

upstream of stop codons would be covered by collided ribosomes (up to pentasomes). Therefore, this part of the mRNA would not be accessible to Hel2 for simultaneous interaction while bound to ribosomes. Only Hel2 molecules bound to the first (terminating/stalled) or last ribosome in the collision would be expected to be able to simultaneously bind mRNA. This is because mRNA between colliding ribosomes is generally inaccessible to non-ribosomal proteins, as exemplified by RNase resistance exploited in disome- or trisome sequencing approaches (21–25). Since the position of the last ribosome in the collision will vary due to the heterogeneity of queues, crosslinking to mRNA upstream of the queue would lead to disperse signals, while the first ribosome in the collision is always the terminating ribosome, possibly allowing a stronger enrichment of crosslinks in the 3′-UTR. (ii) Based on our previous work (1) one of the interaction sites of Hel2 with the ribosomal small subunit is localized close to the mRNA exit channel. Mapping of crosslink sites onto a collided disome structure (Supplementary Figure S9) suggests that Hel2 could simultaneously interact with ribosomes and with mRNA downstream of the mRNA exit channel, whereas simultaneous interactions with mRNA upstream of the Hel2-bound ribosome are less well supported by our data. This suggests that, for several reasons, Hel2 will be more likely to interact with mRNA in the 3′-UTR, as compared to mRNA in the gene body, while bound to ribosomes collided during termination. In addition, this is consistent with our finding that 3′-UTR binding is similar to CDS binding in non-affected mRNAs, somewhat stronger than CDS binding in mildly affected mRNAs, which exhibit short collisions, and much stronger in strongly affected mRNAs, exhibiting longer collisions.

Supplementary Text S2

UV crosslinking and analysis of cDNA reveals New1 interactions with transfer, ribosomal, and messenger RNA

Finally, to address how New1 might protect mRNA from C-terminal ribosome collisions, we performed CRAC using strains in which New1 was (HTP) tagged, and in which collision sensor Hel2 was either present or absent. We compared these data to complementary CRAC data for Hel2 in presence and absence of New1. Here, we found that New1 binds mRNA, rRNA, and tRNA, consistent with a role in translation (elongation and/or termination) (Supplementary Table S12).

New1 interacts with tRNA. The class of New1-bound RNAs that displays the highest difference to Hel2-bound ones is tRNA, representing a substantial ~14 – ~20% of total (non-collapsed) reads in New1-CRAC libraries. Here, we found tR(CCU), the tRNA that decodes the queue-inducing codon 'AGG', to be highly bound, representing between ~0.5 and ~8% of total CRAC reads, and between ~6 and ~38% of total tRNA reads. Despite the apparent inter-replicate variability, this tRNA species represented one of the most bound tRNA species in all New1 CRAC libraries (Supplementary Table S13). Surprisingly, however, tRNAs that decode other strongly affected codons 'AAA' (tK(UUU)) or 'CGU' (tR(ACG/ICG)), were less represented in the New1 CRAC libraries, compared to some other tRNAs decoding various non-affected codons. Structure-wise, it is unclear how New1 might contact tRNAs, and if this happens while New1 is associated with ribosomes. Considering the flexible N- (140 amino acids missing) and C-termini (84 amino acids missing) that have not been resolved in the published cryo-EM structure (2), it might be possible for them to reach tRNAs within translating or stalled ribosomes. However, our current data do not allow for more detailed conclusions.

New1 interacts with the ribosomal small and large subunits and influences ribosomal interactions of collision sensor Hel2. One of the major crosslinked rRNA species was 18S rRNA, in both New1 and Hel2. We used data on single nucleotide micro-deletions, which are frequently induced by nucleotide-amino acid crosslinks (11) to pinpoint sites of direct interaction. In the 18S rRNA, one major (U1361) and two minor (U1491; U493/C495) crosslink sites were observed for New1, where the latter was less prominent. These crosslink sites coincide with the ones previously reported for Hel2 (1), though with a changed preference:

New1 binds more strongly at one of Hel2's minor binding sites, based on the number of reads mapping to the respective positions (Supplementary Fig. S25A). New1's major crosslink site, U1361, is one of the minor crosslink sites observed for Hel2, whereas U1491 is Hel2's major crosslink site. In the published cryo-EM structure of a New1-bound ribosome, this position is in contact with New1 (Supplementary Fig. S25B). By contrast, Hel2's binding at its major crosslink site U1491 was markedly increased in the absence of New1, whereas binding at its minor crosslink site U1361 was relatively decreased (also at 30°C, data not shown). This could be due to the increase in ribosome queuing and collisions observed in the absence of New1 (2, 3). It is also possible that *new1Δ*-induced queues represent a different structure from that of standard collided ribosomes, or that other factors bind these complexes, leading to a different binding geometry of Hel2 to those complexes. Additional crosslinks were observed for New1 and Hel2 in 5S, 5.8S and 25S rRNA, however, compared to 18S crosslink sites, they appeared minor (Supplementary Fig. S25A, left). One crosslink site stood out though: a crosslink site at position U33 of 5S rRNA (Supplementary Fig. S25A, right). Although the 5'-half of 5S rRNA has low read coverage, U33 was the second highest deletion site of this RNA species, being deleted in >10% of reads extending beyond this point. This suggests the position to be a prominent interaction site. Like New1's major 18S rRNA crosslink site, U33 of 5S rRNA clearly makes contact with New1 in the published cryo-EM structure (Supplementary Fig. S25B). This indicates that the structure resolved by cryo-EM, which was determined *ex vivo*, is also relevant in living yeast cells. Our additionally observed 18S rRNA crosslink sites may also be compatible with the cryo-EM structure and could be bound simultaneously, considering that large parts of the New1 N- and C-terminus were not resolved by cryo-EM and are predicted to be highly flexible (Supplementary Fig. S25B). Both, the N- and C-terminus contain positively charged Lys and Arg, as well as aromatic residues, which can mediate RNA binding. As additional evidence for this hypothesis, a crosslinking and mass spectrometry-based technique named identification of RNA-associated peptides (iRAP) (8) determined an RNA binding site at position Arg1170, close to the C-terminus (Supplementary Fig. S25B), which is not visualized by cryo-EM. This position is predicted by alphafold (AF-Q08972-F1) (7) to be in an alpha helix, but is linked to the core of New1 by a long, disordered region. We therefore assume that this position could reach the more distant 18S rRNA crosslink sites.

As an alternative hypothesis, the minor 18S rRNA crosslink sites, but also 5.8S and 25S rRNA crosslink sites we determined could be due to additional binding modes of New1, e.g., during recruitment, potentially in different conformations, which were not captured by cryo-EM (2, 3), so far. The similarities between RNA binding sites of Hel2 and New1 also suggest that binding of these two factors is likely mutually exclusive. Binding of New1 to (stalled or collided) ribosomes might thereby physically block Hel2 from ubiquitinating slowly terminating ribosomes. Its absence, on the other hand, might enable Hel2 activity, thereby terminally stalling such ribosomes, causing or aggravating ribosome queues, and also triggering downstream responses like NGD.

New1 interacts with mRNAs with a preference for 3′-termini, independent of the C-terminal codon. The most-bound RNA species for New1 was mRNA (Supplementary Table S12). Like Hel2, New1 was bound across the mRNA (Supplementary Fig. S26), consistent with a general role in translation, either as an active translation elongation factor, like its homologue eEF3 (27), or sampling ribosomes in different translation states, as recently suggested (3), possibly without exhibiting translation elongation factor activity itself. The fact that overexpression of New1 was able to rescue, to some extent, the growth defect caused by repressed transcription of *YEF3* (2) supports a direct role in translation, which can be exerted at least when necessary. In addition to binding across coding sequences, New1 exhibited a major binding peak in 3′-UTRs. This binding pattern is similar to what we found for Hel2 in the absence of New1, on strongly affected mRNAs. Given that New1, like Hel2, binds to the 18S rRNA close to the mRNA entry channel, we hypothesize that binding to 3′-UTRs could be due to the presence of New1 at ribosomes upstream of or at stop codons, possibly facilitated by the protein's flexible N- and C-termini. However, contrary to Hel2 binding in the absence of New1, New1's binding to 3′-UTRs did not depend on the identity of the C-terminal codon (Supplementary Fig. S26), suggesting New1 is able to associate with terminating ribosomes independently of the C-terminal codon (and tRNA bound within the ribosome). New1 binding to mRNAs was also not altered when comparing CRAC data in presence and absence of Hel2.

In conclusion, our crosslinking data show rRNA interactions of New1 which are in line with published cryo-EM data (2, 3), as well as additional interactions with rRNA, mRNA and tRNA which have not been described before, and which suggest that New1 is not exclusively engaging terminating ribosomes on queuing-prone mRNA C-termini, but ribosomes at all

positions of all mRNAs. This would be in line with structural data showing New1 bound to ribosomes in different translation states (3).

Supplementary References

1. Winz,M.L., Peil,L., Turowski,T.W., Rappsilber,J. and Tollervey,D. (2019) Molecular interactions between Hel2 and RNA supporting ribosome-associated quality control. *Nat Commun*, **10**, 563.

2. Kasari,V., Pochopien,A.A., Margus,T., Murina,V., Turnbull,K., Zhou,Y., Nissan,T., Graf,M., Nováček,J., Atkinson,G.C., *et al.* (2019) A role for the Saccharomyces cerevisiae ABCF protein New1 in translation termination/recycling. *Nucleic Acids Res*, **47**, 8807–8820.

3. Turnbull,K., Paternoga,H., von der Weth,E., Egorov,A.A., Pochopien,A.A., Zhang,Y., Nersisyan,L., Margus,T., Johansson,M.J.O., Pelechano,V., *et al.* (2024) The ABCF ATPase New1 resolves translation termination defects associated with specific tRNAArg and tRNALys isoacceptors in the P site. *Nucleic Acids Res*, **52**, 12005–12020.

4. Ikeuchi,K., Tesina,P., Matsuo,Y., Sugiyama,T., Cheng,J., Saeki,Y., Tanaka,K., Becker,T., Beckmann,R. and Inada,T. (2019) Collided ribosomes form a unique structural interface to induce Hel2-driven quality control pathways. *EMBO J*, **38**.

5. Kertesz,M., Wan,Y., Mazor,E., Rinn,J.L., Nutter,R.C., Chang,H.Y. and Segal,E. (2010) Genome-wide measurement of RNA secondary structure in yeast. *Nature*, **467**, 103–107.

6. Crooks,G.E., Hon,G., Chandonia,J.-M. and Brenner,S.E. (2004) WebLogo: A Sequence Logo Generator. *Genome Res*, **14**, 1188–1190.

7. Jumper,J., Evans,R., Pritzel,A., Green,T., Figurnov,M., Ronneberger,O., Tunyasuvunakool,K., Bates,R., Žídek,A., Potapenko,A., *et al.* (2021) Highly accurate protein structure prediction with AlphaFold. *Nature*, **596**, 583–589.

8. Peil,L., Waghmare,S., Fischer,L., Spitzer,M., Tollervey,D. and Rappsilber,J. (2018) Identification of RNA-associated peptides, iRAP, defines precise sites of protein-RNA interaction. *bioRXiv* doi: 10.1101/456111, 29 October, 2018, pre-print: not peer-reviewed.

9. Brachmann,C.B., Davies,A., Cost,G.J., Caputo,E., Li,J., Hieter,P. and Boeke,J.D. (1998) Designer deletion strains derived from Saccharomyces cerevisiae S288C: a useful set of strains and plasmids for PCR-mediated gene disruption and other applications. *Yeast*, **14**, 115–32.

10. Kostova,K.K., Hickey,K.L., Osuna,B.A., Hussmann,J.A., Frost,A., Weinberg,D.E. and Weissman,J.S. (2017) CAT-tailing as a fail-safe mechanism for efficient degradation of stalled nascent polypeptides. *Science*, **357**, 414–417.

11. Granneman,S., Kudla,G., Petfalski,E. and Tollervey,D. (2009) Identification of protein binding sites on U3 snoRNA and pre-rRNA by UV cross-linking and high-throughput analysis of cDNAs. *Proc Natl Acad Sci U S A*, **106**, 9613–8.

12. Bähler,J., Wu,J.-Q., Longtine,M.S., Shah,N.G., Mckenzie III,A., Steever,A.B., Wach,A., Philippsen,P. and Pringle,J.R. (1998) Heterologous modules for efficient and versatile PCR-based gene targeting in Schizosaccharomyces pombe. *Yeast*, **14**, 943–951.

13. Sitron,C.S., Park,J.H. and Brandman,O. (2017) Asc1, Hel2, and Slh1 couple translation arrest to nascent chain degradation. *RNA*, **23**, 798–810.

14. Janke,C., Magiera,M.M., Rathfelder,N., Taxis,C., Reber,S., Maekawa,H., Moreno-Borchart,A., Doenges,G., Schwob,E., Schiebel,E., *et al.* (2004) A versatile toolbox for PCR-based tagging of yeast genes: new fluorescent proteins, more markers and promoter substitution cassettes. *Yeast*, **21**, 947–962.

15. Davies,A.A. and Ulrich,H.D. (2012) Detection of PCNA Modifications in Saccharomyces cerevisiae. In Bjergbæk,L. (ed), *DNA Repair Protocols*. Humana Press, Totowa, NJ, pp. 543–567.

16. Sitron,C.S., Park,J.H. and Brandman,O. (2017) Asc1, Hel2, and Slh1 couple translation arrest to nascent chain degradation. *RNA*, **23**, 798–810.

17. Gietz,R.D. (2014) Yeast Transformation by the LiAc/SS Carrier DNA/PEG Method. In Smith,J.S., Burke,D.J. (eds), *Yeast Genetics: Methods and Protocols*. Springer, New York, NY, pp. 1–12.

18. Schneider,C.A., Rasband,W.S. and Eliceiri,K.W. (2012) NIH Image to ImageJ: 25 years of image analysis. *Nat Methods*, **9**, 671–5.

19. Bonetti,B., Fu,L., Moon,J. and Bedwell,D.M. (1995) The Efficiency of Translation Termination is Determined by a Synergistic Interplay Between Upstream and Downstream Sequences in *Saccharomyces cerevisiae*. *J Mol Biol*, **251**, 334–345.

20. Cridge,A.G., Crowe-McAuliffe,C., Mathew,S.F. and Tate,W.P. (2018) Eukaryotic translational termination efficiency is influenced by the 3' nucleotides within the ribosomal mRNA channel. *Nucleic Acids Res*, **46**, 1927–1944.

21. Meydan,S. and Guydosh,N.R. (2020) Disome and Trisome Profiling Reveal Genome-wide Targets of Ribosome Quality Control. *Mol Cell*, **79**, 588-602.e6.

22. Guydosh,N.R. and Green,R. (2014) Dom34 rescues ribosomes in 3' untranslated regions. *Cell*, **156**, 950–62.

23. Arpat,A.B., Liechti,A., Matos,M.D., Dreos,R., Janich,P. and Gatfield,D. (2020) Transcriptome-wide sites of collided ribosomes reveal principles of translational pausing. *Genome Res*, **30**, 985–999.

24. Han,P., Shichino,Y., Schneider-Poetsch,T., Mito,M., Hashimoto,S., Udagawa,T., Kohno,K., Yoshida,M., Mishima,Y., Inada,T., *et al.* (2020) Genome-wide Survey of Ribosome Collision. *Cell Rep*, **31**, 107610.

25. Zhao,T., Chen,Y.-M., Li,Y., Wang,J., Chen,S., Gao,N. and Qian,W. (2021) Disome-seq reveals widespread ribosome collisions that promote cotranslational protein folding. *Genome Biol*, **22**, 16.

26. Li,Z., Lee,I., Moradi,E., Hung,N.-J., Johnson,A.W. and Marcotte,E.M. (2009) Rational Extension of the Ribosome Biogenesis Pathway Using Network-Guided Genetics. *PLoS Biol*, **7**, e1000213.

27. Ranjan,N., Pochopien,A.A., Chih-Chien Wu,C., Beckert,B., Blanchet,S., Green,R., V Rodnina,M. and Wilson,D.N. (2021) Yeast translation elongation factor eEF3 promotes late stages of tRNA translocation. *EMBO J*, **40**, e106449.
